# The multicomponent approach to *N*-methyl peptides: total synthesis of antibacterial (–)-viridic acid and analogues

**DOI:** 10.3762/bjoc.8.234

**Published:** 2012-11-28

**Authors:** Ricardo A W Neves Filho, Sebastian Stark, Bernhard Westermann, Ludger A Wessjohann

**Affiliations:** 1 Department of Bioorganic Chemistry, Leibniz Institute of Plant Biochemistry, Weinberg 3, 06120 Halle (Saale), Germany, Tel: +49 345 5582 1301, Fax: +49 345 5582 1309 (Address for correspondence); 2 Martin-Luther-University Halle-Wittenberg, Institute of Organic Chemistry, Kurt-Mothes-Str. 2, 06120 Halle (Saale), Germany

**Keywords:** antibiotic, anticancer, Gram negative bacteria, natural product, peptide coupling, peptides, peptoid, toxin, Ugi reaction

## Abstract

Two syntheses of natural viridic acid, an unusual triply *N*-methylated peptide with two anthranilate units, are presented. The first one is based on peptide-coupling strategies and affords the optically active natural product in 20% overall yield over six steps. A more economical approach with only four steps leads to the similarly active racemate by utilizing a Ugi four-component reaction (Ugi-4CR) as the key transformation. A small library of viridic acid analogues is readily available to provide first SAR insight. The biological activities of the natural product and its derivatives against the Gram-negative bacterium *Aliivibrio fischeri* were evaluated.

## Introduction

Viridic acid (**1**) is a tetrapeptide produced by several fungi of the genus *Penicillium*, including *P. viridicatum*, *P. nordicum*, and *P*. *aurantiogriseum* among others [1–4]. It was first isolated from the basic fraction of the chloroform/methanol extract of *P. viridicatum* Westling [5], and it was assumed to be responsible for the toxicity of this extract due to its metal-chelating properties [6]. Later, this (putative) mycotoxin was also found in cultures of *P. nordicum* cultivated on cheese agar medium. The crude extracts from these cultures displayed pronounced cytotoxicity in a MTT assay on an undisclosed cell line [7]. Albeit that these two reports on the toxicity of extracts containing constituent **1** were very intriguing, no further biological screening of the pure substance has been performed to date. The connection between compound **1** and the bioactivity of the extract containing it is thus purely correlative, i.e., speculative. No causal relationship between the compound and the MTT results is proven.

The structure of compound **1** was determined as the peptide N(Me)_2_Ant-Gly-(N-Me)Val-Ant (Ant = anthranilic acid) in 1986, based on a series of degradation experiments, NMR, and IR measurements as well as a first total synthesis [5]. Thus, it was revealed that **1** belongs to the small group of natural peptides that contain anthranilic acid residues in the peptidic backbone [8–10]. Furthermore, to the best of our knowledge, viridic acid is unique in its N-terminus, which bears a *N*,*N*-dimethyl anthranilic amide moiety of still unknown biosynthetic origin. The previously reported synthetic strategy toward **1** was based upon a series of peptide couplings employing DCC reagent [5]. The necessity of difficult-to-perform peptide couplings with phenyl carboxylates and *N*-methylated amino groups demanded harsher than usual conditions, upon which the desired viridic acid (**1**) was obtained in just 5% overall yield.

**Scheme 1:**
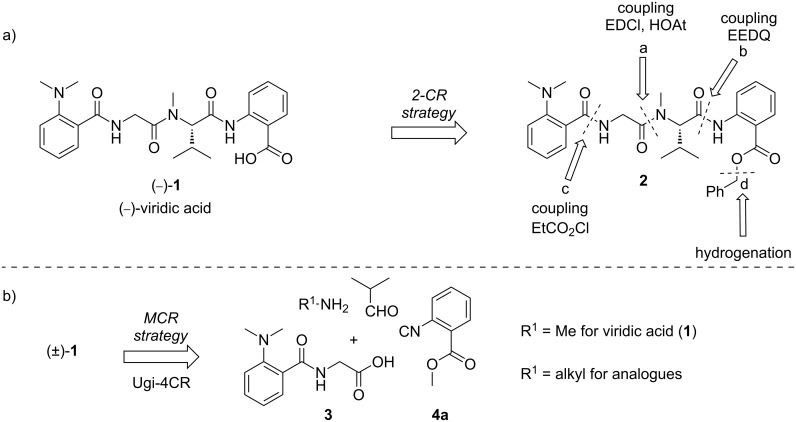
Retrosynthetic strategies for (−)-viridic acid (**1**) and analogues by (a) a bidirectional peptide-coupling sequence, and (b) a novel MCR approach.

The lack of sufficient amounts of isolated materials from natural sources did not allow for reliable bioactivity tests, and the demand for higher quantities and derivatives of viridic acid (**1**), required the development of a more efficient and milder approach. In this endeavor we envisioned two routes. The first one uses improved peptide-coupling protocols, leading to the natural enantiomer (Scheme 1) [11]. The blueprint of the synthesis was planned as a bidirectional sequence from the least- to the most-reactive amine (NMe-Val < H-Ant-OBn < H-Gly) to yield the protected tetrapeptide **2** (Scheme 1a). Alternatively, a multicomponent (MCR) approach based on a Ugi four-component reaction (Ugi-4CR) of dipeptide **3**, isobutyraldehyde, methylamine and isonitrile **4** as the key transformation was envisioned to yield racemic viridic acid (±)-**1** (Scheme 1b) [12]. Besides furnishing the desired natural product in only four steps, the MCR approach allows a ready access to analogues endowed with a proteolysis-resistant peptoid moiety [13]. Recently, we demonstrated that chemically more stable peptoid analogues of tubulysins, named tubugis, present cytotoxicity against cancer cell lines comparable to the native natural product [14]. Thus, it was hoped that some viridic acid analogues readily accessible by MCR may also display enhanced biological activity or at least stability.

## Results and Discussion

**Scheme 2:**
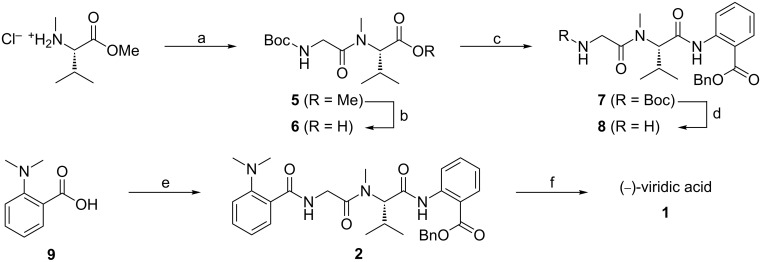
Reactions and conditions: (a) Boc-Gly-OH, EDCI, HOAt, TEA, CH_2_Cl_2_, rt, 20 h, 73%. (b) LiOH, THF/H_2_O (1:1), rt, 3 h, 97%. (c) Benzyl anthranilate, EEDQ, CHCl_3_, rt, 20 h, 51%. (d) TFA, CH_2_Cl_2_, rt, 5 h. quant. (e) **8**, NMM, ethyl chloroformate, CHCl_3_, rt, 20 h, 85%. (f) H_2_, 10% Pd/C, MeOH, rt, 16 h, 92%.

The 2-CR approach based on the peptide coupling of Boc-Gly and NMe-Val-OMe in the presence of EDCl and HOAt gave the central dipeptide **5** in 73% yield (Scheme 2) [15].The use of even more activating coupling reagents, such as HATU and BOP, was also investigated and resulted in increased formation of side products [11]. After saponification of intermediate **5** to dipeptide **6**, the latter was coupled with benzyl anthranilate. This reaction was particularly challenging due to the very poor reactivity of this combination [10]. All attempts to perform this coupling with carbodiimides, HBTU, HATU, and PyBroP failed or resulted in very low conversions. The addition of a catalytic amount of DMAP to the carbodiimide-mediated reactions improved the conversions, but resulted in severe racemization. The best result was obtained by employing *N*-ethoxycarbonyl-2-ethoxy-1,2-dihydroquinoline (EEDQ) as coupling reagent, which gave the optically active tripeptide **7** in 51% yield [16–17]. Intermediate **7** was converted into amine **8** under acidic conditions, and coupled directly to *N*,*N*-dimethylanthranilic acid (**9**). It was already reported that carbodiimide-mediated couplings involving aromatic carboxylic acids such as **9** can result in the formation of *N*-acylurea adducts through competitive N–O rearrangement [18–19]. In order to overcome this problem the mixed-carbonate method was used [20]. The reaction resulted in the desired optically active key intermediate **2** in 85% yield. Its hydrogenation afforded the desired (−)-viridic acid (**1**) in quantitative conversion and >92% isolated yield, i.e., in 20% overall yield. Analytical data such as the HRMS, ^1^H NMR, melting point, and optical rotation of synthetic compound **1** are consistent with the data reported for the natural substance (Scheme 2) [5].

**Scheme 3:**
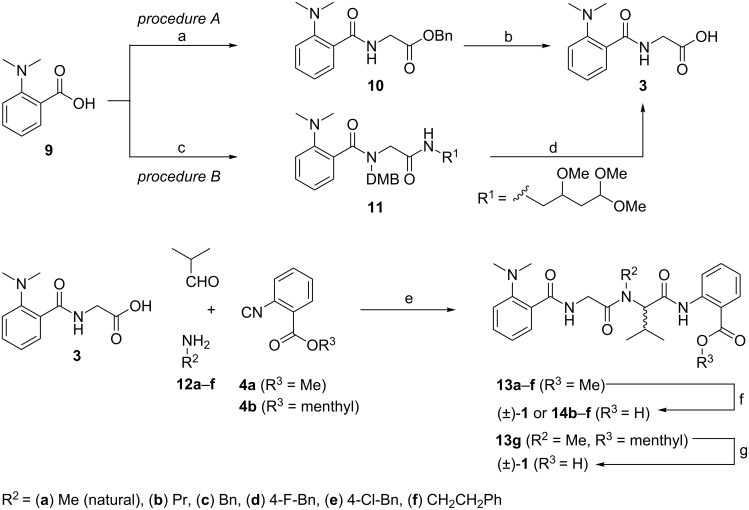
Reactions and conditions: (a) H-Gly-OBn·HCl, NMM, ethyl chloroformate, CHCl_3_, 18 h, rt, 88%. (b) H_2_, 10% Pd/C, MeOH, rt, 16 h, 98%. (c) 2,4-Dimethoxybenzylamine, formaldehyde, IPB, MeOH, rt, 18 h, 35%. (d) TFA, CH_2_Cl_2_, 0 °C to rt, 48 h, then LiOH, THF/H_2_O (1:1), rt, 3 h, 21% (over two steps). (e) MeOH, rt, 20 h, 51–70%. (f) LiOH, THF/H_2_O, rt, 8 h, 81–91%. (g) KOH, MeOH/H_2_O (3:1), rt, 8 h, 70%.

In order to more rapidly access derivatives for biological activity screens, we decided to investigate the suitability of a MCR approach utilizing the Ugi reaction. Due to the character of the Ugi-4CR, the racemate of viridic acid and congeners is easily available, and assaying with (±)-**1** can give an estimation of the relevance or effect of the configuration of the asymmetric center on the biological activity. With this goal in mind, the Ugi-4CR between methylamine, isobutyraldehyde, dipeptide **3**, and the anthranilate-derived isonitrile **4a** was planned (Scheme 1) [21]. For the synthesis of the N-terminal dipeptide **3**, amino acid **9** was coupled with benzyl glycinate in the presence of ethyl chloroformate to give **10** in 88% yield. This intermediate was hydrogenated to afford the dipeptide acid **3** quantitatively. An alternative multicomponent approach to dipeptide **3** requires the use of ammonia or an ammonia equivalent, such as 2,4-dimethoxybenzylamine (DMB-NH_2_), as amino component, formaldehyde as oxo-component, and a convertible isonitrile [22–25]. Although many convertible isonitriles are reported in the literature [24,26], the recently developed 4-isocyanopermethylbutane-1,1,3-triol (IPB) was chosen due to its ease of preparation, better reactivity, and mild conversion conditions [27]. The Ugi-4CR involving carboxylic acid **9**, DMB-NH_2_, formaldehyde and IPB resulted in intermediate **11** in 35% yield. A tandem DMB group cleavage/pyrrole-formation sequence under acidic conditions followed by saponification afforded the desired carboxylic acid **3** in 21% yield over the two steps (Scheme 3). The MCR approach to building block **3** with two convertible components gives lower yields compared to the classical amide formation, but it carries the diversity-generating ability inherent in Ugi-4CRs, and the potential to synthesize derivatives where classical methods are less suitable.

The key Ugi-4CR combining isobutyraldehyde, methylamine (**12a**), dipeptidic carboxylate **3** and anthranilic isonitrile **4a**, to form the final tetrapeptide backbone was performed by using standard protocols, with imine preformation in methanol, to give **13a** in 51% yield. Finally, saponification of **13a** afforded the racemic viridic acid (±)-**1** in 83% yield. Attempts to improve the MCR yield with conventional or microwave heating, or by employing trifluoroethanol or DMF as solvents resulted in poor conversions, with competitive Passerini reaction in the latter case [28–29]. With the general process in place, the MCR approach was employed to generate a library of synthetic derivatives of **1** with the hope of gaining a first glimpse of structure–activity relationships (SAR), and to give hints for further applications, as for example for attachment points for probe design and experiments [30–31]. Thus, methylamine was substituted by **12b**–**f** in the key Ugi-4CR to yield the intermediates **13b**–**f** (55–70% yields) [16], which afforded the desired peptoid analogues **14b**–**f** after saponification. Compared to simple peptides, N-alkylated ones (peptoid moieties) allow different low-energy conformations, and contrary to common belief they are more restricted in conformational space [32]. Moreover, they commonly possess a higher lipophilicity and protease stability, and this combination seems to improve their antibiotic properties (Scheme 3) [13].

Since the MCR results in racemates, we decided to investigate the applicability of a chiral auxiliary MCR approach for the asymmetric synthesis of viridic acid (**1**). The Ugi-4CR is not specifically prone to asymmetric induction, but at least some auxiliaries are known to result in preferential formation of a diastereoisomer [33–34]. Therefore, the isonitrile **4b** was synthesized from menthyl anthranilate. The Ugi-4CR of **4b** in analogy to the reaction of **4a** with methylamine (**12a**) gave the desired peptoid–peptide adduct **13g** in 47% yield, albeit as a 1:1 diastereomeric mixture. Unfortunately, even a separation of the epimers failed by using thin- or thick-layer or conventional column chromatography or HPLC, under varied conditions of different column types, methods, mobile-phase compositions, etc. This is in accordance with earlier results, where also no or only negligible diastereoselection could be achieved [35–36]. Saponification of the menthyl ester **13g** afforded the racemic viridic acid (**1**) again in 70% yield (Scheme 3) [37].

To our knowledge no biological screening of pure (−)-viridic acid or its analogues has been performed, and due to the potential of natural peptides and peptoids as antibacterial agents [13,38–47], it was decided to investigate their activity against the Gram-negative bacterium *Aliivibrio fischeri* [48]. Compounds (−)-**1** and (±)-**1** were the most active ones with IC_50_ values of 45.0 ± 4.4 and 38.4 ± 5.8 μM, respectively. In this test system, derivatives **14b**–**f** displayed IC_50_ values above 100 μM and can be considered as inactive. Although (−)-viridic acid (**1**) was isolated thirty years ago, this is the first unambiguous report concerning its biological activity. Based upon the results presented above, it seems that the configuration of the stereogenic center has almost no influence on the antibacterial effect of **1**. The lack of activity of the derivatives **14b**–**f** is difficult to rationalize without knowing the target, but it demonstrates that the size of the group attached to the nitrogen of the Val residue has a clear influence. This fact suggests that **1** does not act just by engaging bacterial membranes as most antibacterial peptides do [49], but that it may bind to a specific target.

## Conclusion

These results highlight the usefulness of the Ugi-4CR for the diversity-oriented synthesis of natural *N*-methyl peptides, such as viridic acid and its derivatives. Considering the attractiveness of the anthranilic acid moiety as a promising building block for drug-like molecules and the diverse properties exhibited by natural products containing it [8,50], further biological trials involving such components are currently being pursued. The advantages of the MCR protocol are speed, variability, insensitivity to steric crowding, safe peptoid-moiety formation, and access to equally distributed stereoisomers (which can be a disadvantage though, once the most active isomer is identified).

The improved classical approach gave the natural product in much lower overall yield and more steps but, after careful choice of conditions, in optically pure form. A set of N-alkylated derivatives were screened against *Aliivibrio fischeri*, but only the (*N*-methylated) natural product displayed noteworthy activity of ca. 40 μM IC_50_, independent of stereochemistry.

## Supporting Information

File 1 Complete experimental procedures and characterization.

File 2 Figures of ^1^H and ^13^C NMR spectra.
