# Supplementary material for: The multicomponent approach to N-methyl peptides: total synthesis of antibacterial (–)-viridic acid and analogues
Source: Beilstein J Org Chem. 2012 Nov 28;8:2085–90. doi: 10.3762/bjoc.8.234 (PMC3511043; doi:10.3762/bjoc.8.234)
Supplement: File 1 — Complete experimental procedures and characterization. [file Beilstein_J_Org_Chem-08-2085-s001.pdf]

Supporting Information File 1

for

The multicomponent approach to *N*-methyl peptides: total synthesis of antibacterial (–)-viridic acid and analogues

Ricardo A. W. Neves Filho[1], Sebastian Stark [1,2], Bernhard Westermann[1,2] and Ludger A. Wessjohann[*1,2]

Address: [1]Department of Bioorganic Chemistry, Leibniz Institute of Plant Biochemistry, Weinberg 3, 06120 Halle (Saale), Germany, Tel: +49 345 5582 1301, Fax: +49 345 5582 1309 (Address for correspondence) and [2]Martin-Luther-University Halle-Wittenberg, Institute of Organic Chemistry, Kurt-Mothes-Str. 2, 06120 Halle (Saale), Germany

Email: Ludger A. Wessjohann - wessjohann@ipb-halle.de.

* Corresponding author:

Complete experimental procedures and characterization

General remarks

All commercially available chemicals were used without further purification. Isocyanopermethylbutane-1,1,3-triol (IPB) was prepared according to the reported procedure [1]. Dichloromethane and THF were dried before use by following conventional procedures. HPLC grade methanol was used in Ugi-4CR reactions. Analytical thin-layer chromatography (TLC) was performed by using silica gel 60 F254 aluminum sheets and the visualization of the spots was done under UV light (254 nm) or by charring with a solution of ninhydrin 0.2% in *n*-butanol containing 1% of acetic acid. Flash column chromatography was performed on silica gel (0.040–0.063 mm). Melting points are uncorrected. ^{1}H and ^{13}C NMR spectra were recorded in solution at 22 °C at 400 MHz and 100 MHz, respectively. Chemical shifts (δ) are reported in ppm relative to the TMS (^{1}H NMR) or to a solvent signal (^{13}C NMR spectra). HRMS spectra were obtained from a Bruker Apex III Fourier transform ion cyclotron resonance (FT-ICR) mass spectrometer equipped with an Infinity™ cell, a 7.0 Tesla superconducting magnet, an RF-only hexapole ion guide and an external electrospray ion source (off axis spray).

Preparation of benzyl anthranilate [2]

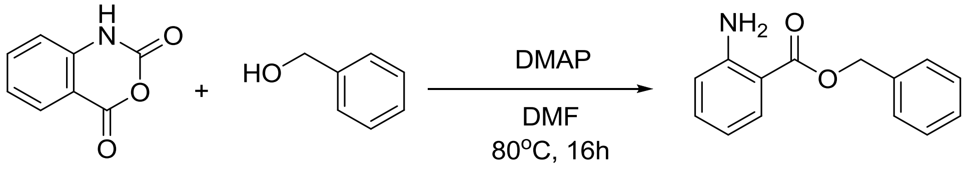

To a stirred solution of isatoic anhydride (16.3 g, 100 mmol) and DMAP (1.2 g, 10 mmol) in DMF (100 mL), benzyl alcohol (12.4 mL 120 mmol) was added

under N_2 atmosphere. The mixture was stirred at 80 °C for 16 h. The reaction was cooled to room temperature and partitioned between water (500 mL) and ethyl acetate (500 mL). The aqueous phase was further extracted with ethyl acetate (2 × 100 mL). The organic layers were combined, washed with brine (2 × 100 mL) and dried over sodium sulfate. The solvent was evaporated under reduced pressure. The crude material was re-crystallized from hot hexanes to afford 18.4 g of the desired product as white crystals.

Yield: 81%; ^{1}H NMR ($CDCl_3$) δ = 5.32 (s, 2H), 5.72 (br, 2H), 6.60–6.67 (m, 2H), 7.21 (td, *J* = 7.7, 1.4 Hz, 1H), 7.32–7.47 (m, 5H), 7.94 (dd, *J* = 7.7, 1.4 Hz, 1H).

Preparation of (*S*)-methyl 3-methyl-2-(methylamino)butanoate hydrochloride

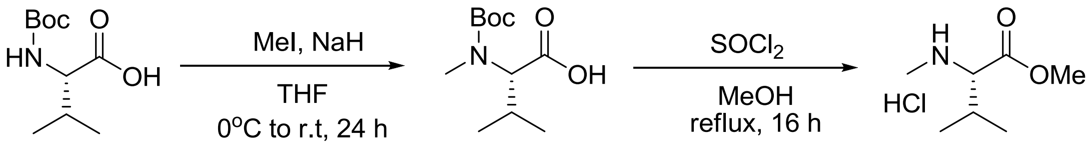

(*S*)-2-(*tert*-Butoxycarbonyl(methyl)amino)-3-methylbutanoic acid [3]

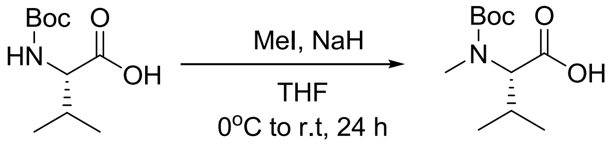

To a stirred solution of N-Boc-L-Val-OH (3.0 g, 14.0 mmol) and methyl iodide (20.0 g, 8.8 mL, 140 mmol) in anhydrous THF (100 mL) at 0 °C was added sodium hydride (60% dispersion in mineral oil; 3.0 g, 138 mmol) in portions of 1.0 g each 10 min). The mixture was stirred at room temperature for 24 h under

N_2 atmosphere. The reaction was cooled to 0 °C and was carefully quenched by adding water (20 mL). The THF was evaporated under reduced pressure. The remaining content was diluted with water (300 mL) and washed with diethyl ether (4 × 50 mL). The aqueous phase was acidified to pH 3.0 with a saturated $NaHSO_4$ solution and extracted with ethyl acetate (200 mL). The organic layer was washed with $Na_2S_2O_5$ aqueous solution (30% w/w, 50 mL) and brine (50 mL) and was dried over Na_2SO_4. The solvent was evaporated under reduced pressure to afford 3.1 g of a colorless oil, which was used in the next step without further purification.

Yield: 73%. $[\alpha]_D^{22}$ −71.5 (*c* 2.94, $CHCl_3$) (lit. [4]: $[\alpha]_D^{20}$ −85.0 (*c* 0.5, EtOH)) 4]; ^{1}H NMR ($CDCl_3$) δ = 0.85 (d, *J* = 6.8 Hz, 3H), 0.96 (d, *J* = 6.8 Hz, 3H), 1.41(s, 9H), 2.21 (m, 1H), 2.81 (s, 3H), 4.05 (br,1H).

(*S*)-Methyl 3-methyl-2-(methylamino)butanoate hydrochloride

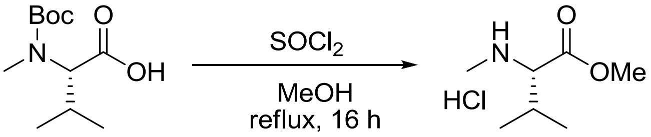

To a solution of Boc-N-L-Me-Val (1.2 g, 5 mmol) in dry methanol (100 mL) at 0 °C was added thionyl chloride (5.9 g, 3.7 mL, 50 mmol) dropwise. The mixture was stirred under reflux for 16 h. Afterwards the solvent was removed under reduced pressure. Toluene (30 mL) was added and then evaporated under reduced pressure. This operation was repeated twice to afford a light yellow oil (0.9 g), which crystallizes spontaneously after drying for 24 h in an auto-vacuum pump. This product was used in the next step without further purification.

Yield: 99%; $[\alpha]_D^{22}$ +29.4 (*c* 1.94, $CHCl_3$);^{1}H NMR (CD_3OD) δ = 1.04 (m, 3H), 1.11 (m, 3H), 2.33 (m, 1H), 2.73 (s, 3H), 3.18 and 3.94 (d, *J* = 4.0 Hz, 3H), 3.87 (s, 3H).

Preparation of methyl 2-isocyanobenzoate (4a) [5].

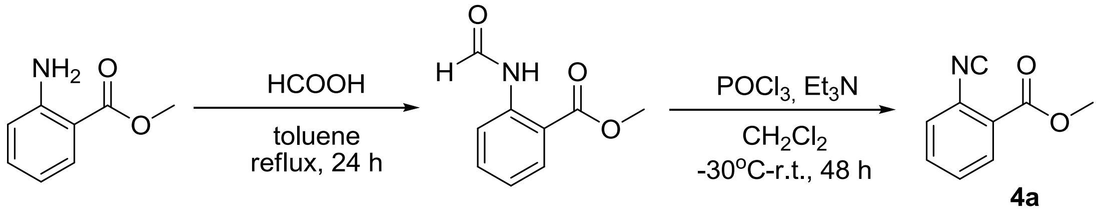

4a

Methyl 2-formamidobenzoate

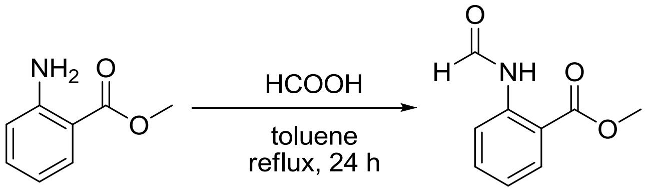

In a 500 mL round-bottom flask equipped with a Dean-Stark trap and reflux condenser were added methyl anthranilate (15.1 g, 100 mmol), formic acid 98% (50 mL) and toluene (150 mL). The mixture was stirred under reflux for 24 h. After this period another amount of formic acid 98% (50 mL) was added and heating under reflux continued for further 36 h. The reaction mixture was cooled to room temperature, and the solvent was evaporated to dryness under reduced pressure to afford a solid material which was used in the next step without further purification.

Methyl 2-isocyanobenzoate (**4a**)

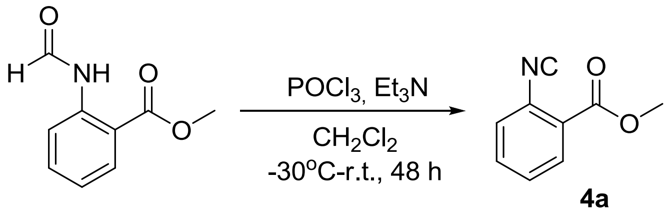

A solution of methyl 2-formamidobenzoate (9.0 g, 50 mmol) and triethylamine (17.6 ml, 125 mmol) in anhydrous dichloromethane (512 mL) was cooled to −20 °C. Subsequently, phosphoryl chloride (6.42 mL, 70 mmol) was added, while the temperature was maintained between −30 °C and −20 °C. After complete addition the reaction was stirred for 30 min at −30 °C and afterwards warmed to room temperature. The mixture was stirred for 48 h at ambient temperature before it was cooled to 0 °C and quenched with a concentrated aqueous solution of $NaHCO_3$ (300 mL). The resulting emulsion was vigorously stirred for ten minutes at 0 °C before the layers were separated. Afterwards the organic phase was washed with sodium chloride solution (5% w/w, 2 × 100 mL) and brine (100 mL) was and then dried over Na_2SO_4 and filtered. After evaporation of the solvents in vacuo the residue was purified by column chromatography (hexane/ethyl acetate 9:1) and **4a** (6.52 g) was isolated as a light-red oil.

Yield: 81%; ^{1}H NMR ($CDCl_3$) δ = 3.98 (s, 3H), 7.47–7.51 (m, 2H), 7.58 (t, *J* = 7.6 Hz, 1H), 8.02 (dd,*J* = 7.6, 2.0 Hz, 1H); ^{13}C NMR ($CDCl_3$) δ = 52.6, 125.4, 126.9, 128.8, 129.1, 137.2, 133.0, 164.4, 169.4.

Methyl (*S*)-2-(2-(*tert*-butoxycarbonylamino)-*N*-methylacetamido)-3-methylbutan-oate (**5**) [6]

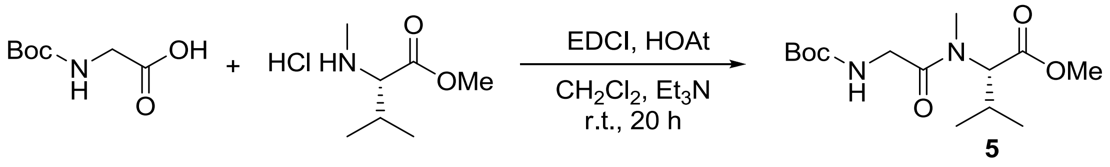

To a solution of Boc-Gly-OH (1.05 g, 6.0 mmol) in dichloromethane (10 mL) were added NMe-Val-OMe·HCl (0.91 g, 5 mmol), EDCI (1.15 g, 6 mmol), HOAt (12 mL, 0.5 M solution in DMF, 6.0 mmol) and triethylamine (1.0 mL, 7.0 mmol). After stirring for 20 h, the solvent was removed under reduced pressure. The crude material was purified by silica-gel column chromatography using a gradient of (2:8 → 5:5) ethyl acetate:hexanes as eluents to afford 1.17 g of **5** as a colorless oil.

Yield: 73%; $[\alpha]_D^{22}$ −62.0 (*c* 1.0, $CHCl_3$); ^1H NMR ($CDCl_3$) δ = 0.76 and 0.81 (d, *J* = 7.2 Hz, 3H), 0.89 (m, 3H), 1.35 (s, 9H), 2.13 (m, 1H), 2.81 and 2.89 (s, 3H), 3.61 and 3.64 (s, 3H), 3.93 and 3.99 (s, 2H), 4.80 (d, *J* = 10.8 Hz, 1H), 5.55 (bs, 1H); ^{13}C NMR ($CDCl_3$) δ = 18.1, 18.4, 18.9, 19.0, 26.9, 27.8, 29.6, 42.1, 51.4, 51.7, 61.2, 64.1, 78.9, 84.3, 155.2, 168.5, 169.0, 169.4, 170.6.

(*S*)-2-(2-(*tert*-Butoxycarbonylamino)-*N*-methylacetamido)-3-methylbutanoic acid (**6**)

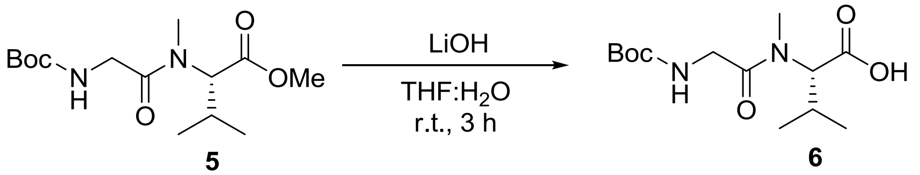

To a solution of **5** (0.6 g, 2.0 mmol) in a mixture of THF (4 mL) and water (4 mL) was added $LiOH \cdot H_2O$ (0.16 g, 4.0 mmol) in one portion. After stirring for 3 h, the mixture was transferred to a separatory funnel. The solution was acidified to pH 3.0 by using saturated $NaHSO_4$ solution, and then brine (30 mL) was added. The contents were extracted with EtOAc (3 × 20 mL). The organic layer was separated and dried over Na_2SO_4 and the solvent was removed under reduced pressure to afford 0.56 g of a colorless oil, which was used in the next step without further purification.

Yield: 97%; $[\alpha]_D^{22}$ −66.1 (*c* 1.0, $CHCl_3$); ^{1}H NMR ($CDCl_3$) δ = 0.78 (d, *J* = 7.2 Hz, 3H), 0.97 (d, *J* = 7.2 Hz, 3H), 1.35 (s, 9H), 2.18 (m, 1H), 2.85 and 2.90 (s, 3H), 3.96 (m, 2H), 4.77 (d, *J* = 10.0 Hz, 3H), 5.74 (bs, 1H), 10.48 (bs, 1H); ^{13}C NMR ($CDCl_3$) δ = 18.4, 18.8, 19.3, 19.5, 27.2, 28.1, 30.5, 42.3, 60.3, 62.0, 64.5, 79.8, 84.5, 156.0, 169.4, 169.9, 171.6, 173.0; HRMS (*m*/*z*): $[M + H]^+$ calcd for $C_{13}H_{24}N_2O_5$, 289.1763; found, 289.1759.

(*S*)-Benzyl 2-(2-(2-(*tert*-butoxycarbonylamino)-*N*-methylacetamido)-3-methyl-butanamido)benzoate (**7**)

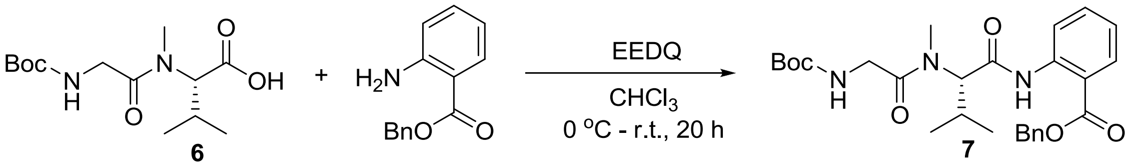

To a solution of **6** (0.52 g, 1.8 mmol) and benzyl anthranilate (0.46 g, 2.0 mmol) in dry chloroform (20 mL) at 0 °C, EEDQ (1.35 g, 5.5 mmol) in dry chloroform (10 mL) was added. The contents were stirred at 0 °C for 1 h and then for a

further 20 h at room temperature. The solvent was removed under reduced pressure and dichloromethane (100 mL) was added. This solution was transferred to a separatory funnel and washed with cold HCl (0.5 M, 3 × 50 mL) and brine (2 × 50 mL). The organic phase was dried over Na_2SO_4, the solvent was removed under reduced pressure, and the crude material was purified by column chromatography by using a gradient of (0:1 → 1:9) ethyl acetate:dichloromethane as eluents to afford 0.46 g of **7** as a colorless oil.

Yield: 51%; $[\alpha]_D^{22}$ −16.22 (*c* 1.0, $CHCl_3$); ^{1}H NMR ($CDCl_3$) δ = 0.81 (d, *J* = 7.2 Hz, 3H), 0.97 (d, *J* = 7.2 Hz, 3H), 1.35 (s, 9H), 2.30 (m, 1H), 2.87 (s, 3H), 3.96 (m, 2H), 4.83 (d, *J* = 10.8 Hz, 1H), 5.28 (s, 2H), 5.63 (bs, 1H), 6.98 (td, *J* = 8.0, 1.2 Hz, 1H), 7.25–7.35 (m, 5H), 7.41 (td, *J* = 8.0, 1.2 Hz, 1H), 7.97 (dd, *J* = 8.0, 1.6 Hz, 1H), 8.58 (d, *J* = 8.0 Hz, 1H), 11.16 (s, 1H); ^{13}C NMR ($CDCl_3$) δ = 18.4, 19.6, 26.1, 28.0, 29.5, 42.3, 63.8, 66.8, 79.1, 115.2, 120.2, 122.6, 128.0, 128.2, 128.3, 130.6, 134.2, 135.1, 140.6, 155.4, 167.3, 168.3, 169.3; HRMS (*m*/*z*): $[M + Na]^+$ calcd for $C_{27}H_{35}N_3O_6Na$, 520.2424; found, 520.2401.

(*S*)-Benzyl 2-(2-(2-amino-*N*-methylacetamido)-3-methylbutanamido)benzoate 2,2,2-trifluoroacetate (**8**)

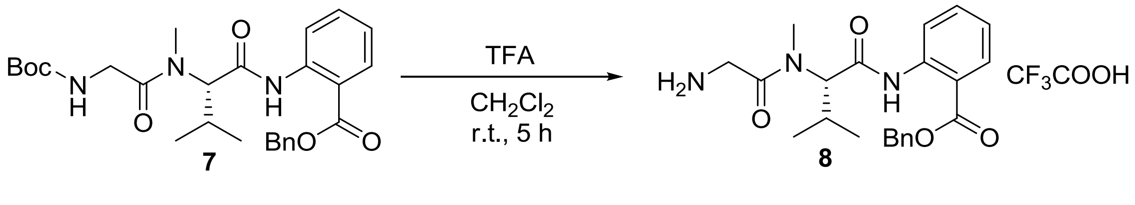

To a solution of **7** (0.4 g, 0.8 mmol) in dichloromethane (15 mL), trifluoroacetic acid (1 mL) was added and the mixture was stirred for 5 h. The end of the

reaction was confirmed by ESIMS analysis. The solvent was removed under reduced pressure. To the crude material was added toluene (20 mL) and the contents were concentrated under reduced pressure to dryness. This operation was repeated two times in order to remove remaining amounts of TFA. The crude product was used in the next step without further purification.

(*S*)-Benzyl 2-(2-(2-(2-(dimethylamino)benzamido)-*N*-methylacetamido)-3-methylbutanamido)benzoate (**2**)

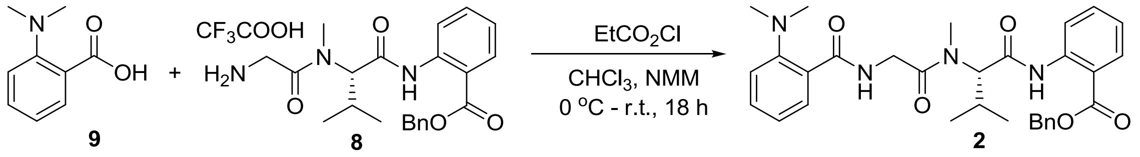

To a solution of 2-(dimethylamino)benzoic acid (**9**) (0.17 g, 1.0 mmol) in chloroform (10 mL) at 0 °C was added *N*-methylmorpholine (0.15 g, 0.16 mL, 1.5 mmol). After stirring for 30 min under N_2 atmosphere, ethyl chloroformate (0.16 g, 0.14 mL, 1.5 mmol) was added while stirring was continued for a further 45 min, after which, a solution of **8** (0.31 g, 0.8 mmol) and *N*-methylmorpholine (0.15 g, 0.16 mL, 1.5 mmol) in chloroform (10 mL) was added. After stirring for another 30 min, the cooling bath was removed and the contents were stirred at room temperature for 20 h. The solvent was removed under reduced pressure and the crude material was purified by silica-gel column chromatography using a gradient of (2:8 → 5:5) ethyl acetate:hexanes as eluent to afford 0.37 g of **2** as a colorless oil.

Yield: 85%; $[\alpha]_D^{22}$ −38.40 (c 1.0, $CHCl_3$); ^1H NMR ($CDCl_3$) δ = 0.88 (d, J = 7.2 Hz, 3H), 1.06 (d, J = 7.2 Hz, 3H), 2.37 (m, 1H), 2.77 (s, 6H), 2.95 (s, 3H), 4.39 (t, J = 4.0 Hz , 2H), 4.98 (d, J = 10.8 Hz, 1H), 5.27 (s, 2H), 7.03 (td, J = 8.0, 1.2 Hz, 1H), 7.26–7.39 (m, 12H), 7.47 (td, J = 8.0, 1.2 Hz, 1H), 8.02 (dd, J = 8.0, 1.6 Hz, 1H), 8.59 (d, J = 8.0 Hz, 1H), 8.64 (d, J = 8.0 Hz, 1H), 10.56 (bs, 1H), 11.23 and 11.53 (s, 1H); ^{13}C NMR ($CDCl_3$) δ = 18.5, 19.8, 26.2, 29.9, 42.3, 45.1, 63.7, 66.7, 115.3, 119.7, 120.4, 122.7, 123.7, 126.8, 128.0, 128.2, 128.3, 128.4, 130.8, 131.0, 131.8, 134.4, 135.14, 140.8, 166.3, 167.4, 168.5, 169.5. HRMS (m/z): $[M + Na]^+$ calcd for $C_{31}H_{36}N_4O_5Na$, 567.2583; found, 567.2577.

(−)-Viridic acid, [(S)-2-(2-(2-(2-(dimethylamino)benzamido)-N-methylacetamido)-3-methylbutanamido)benzoic acid] (**1**)

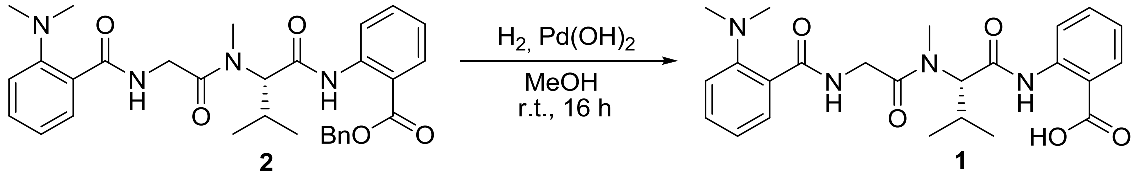

2 **1**

To a stirred solution of compound **2** (0.36 g, 0.7 mmol) in MeOH (10 mL) was added Pd/C (36 mg, 10% w/w). The reaction vessel was evacuated, purged with hydrogen and kept under a H_2 atmosphere (1 atm). The suspension was stirred for 16 h at room temperature. After filtration over Celite, the solvent was removed under reduced pressure to yield a white solid, which was further purified by silica-gel column chromatography using a gradient of (1:9 → 7:3) methanol:dichloromethane as eluent to afford 0.29 g of **1** as a colorless solid.

Yield: 92%; $[\alpha]_D^{22}$ −36.4 (*c* 1.0, $CHCl_3$) (lit. [7]: $[\alpha]_D^{19}$ −69.7 (*c* 1.0, $CHCl_3$) [7]); ^{1}H NMR (DMSO-d_6) δ = 0.84 (d, *J* = 6.8 Hz, 3H), 1.01 (d, *J* = 6.8 Hz, 3H), 2.27 (m, 1H), 2.70 (s, 6H), 3.06 (s, 3H), 4.35 (d, *J* = 4.4 Hz, 2H), 4.76 (d, *J* = 10.8 Hz, 1H), 6.99 (m, 1H), 7.11 (m, 1H), 7.26 (m, 1H), 7.32 (m, 1H), 7.43 (m, 1H), 7.86 (dd, *J* = 8.0, 1.2 Hz, 1H), 8.02 (dd, *J* = 8.0, 1.6 Hz, 1H), 8.46 (t, *J* = 8.0 Hz, 1H), 9.86 (t, *J* = 4.4 Hz, 1H); ^{13}C NMR ($CDCl_3$) δ = 19.1, 20.2, 26.9, 30.6, 41.5, 44.7, 63.6, 118.7, 119.4, 121.8, 122.5, 123.8, 126.6, 130.6, 131.8, 140.3, 152.3, 166.1, 168.4, 169.4, 171.1; HRMS (*m/z*): $[M + Na]^+$ calcd for $C_{24}H_{30}N_4O_5Na$, 477.2114; found, 477.2120.

2-(2-(Dimethylamino)benzamido)acetic acid (**3**)

Procedure A: Starting from anthranilate **9**.

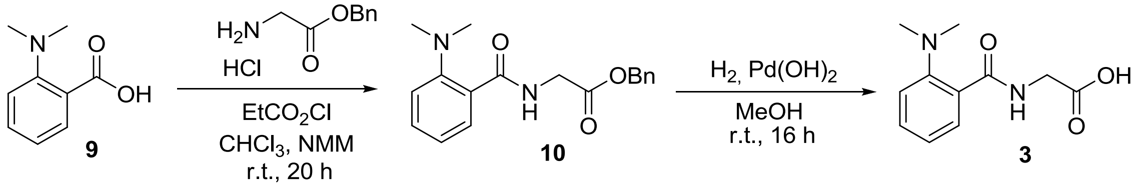

Benzyl 2-(2-(dimethylamino)benzamido)acetate (**10**)

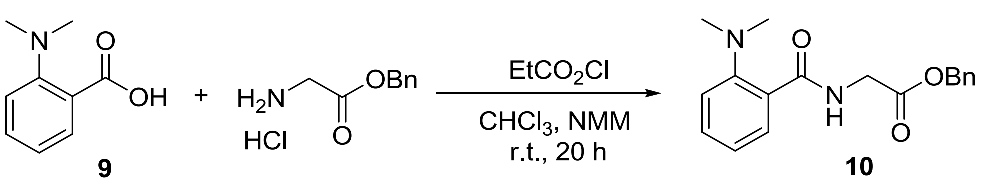

To a solution of 2-(dimethylamino)benzoic acid (3.3 g, 20 mmol) in chloroform (200 mL) at 0 °C and was added *N*-methylmorpholine (3.0 g, 3.2 mL, 30 mmol). This mixture was stirred for 30 min under N_2 atmosphere. Ethyl chloroformate

(3.2 g, 2.8 mL, 30 mmol) was added followed by a further 45 min of stirring. Afterwards, a solution of glycine benzylester hydrochloride (5.4 g, 25 mmol) and *N*-methylmorpholine (3.0 g, 3.2 mL, 30 mmol) in chloroform (100 mL) was added. After stirring for 30 min, the cooling bath was removed and the contents were stirred at room temperature for 20 h. The solvent was removed under reduced pressure and the crude material was purified by silica-gel column chromatography using a gradient of (2:8 → 5:5) ethyl acetate:hexanes as eluents to afford 5.49 g of **10** a colorless oil.

Yield: 88%; ^{1}H NMR ($CDCl_3$) δ = 2.67 (s, 6H), 4.28 (d, *J* = 5.2 Hz, 2H), 5.16 (s, 2H), 7.13 (t, *J* = 7.6 Hz, 1H), 7.18 (d, *J* = 7.6 Hz, 1H), 7.26–7.40 (m, 6H), 8.13 (dd, *J* = 7.6, 1.6 Hz, 1H), 10.44 (br, 1H); ^{13}C NMR ($CDCl_3$) δ = 41.4, 45.0, 66.6, 119.8, 123.9, 126.4, 127.9, 128.0, 128.1, 128.2, 131.0, 131.9, 135.0, 166.2, 169.7; HRMS (*m*/*z*): $[M + Na]^+$ calcd for $C_{18}H_{20}N_2O_3Na$, 335.1372; found, 335.1365.

2-(2-(Dimethylamino)benzamido)acetic acid (**3**)

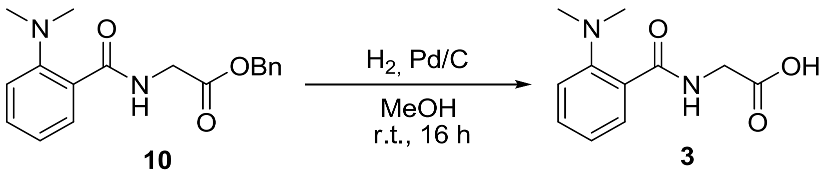

To a stirred solution of compound **10** (5.0 g, 16 mmol) in MeOH (100 mL) was added Pd/C (0.5 g, 10% w/w). The reaction vessel was evacuated, purged with hydrogen and kept under H_2 atmosphere (1 atm). The suspension was stirred for 16 h at room temperature. After filtration over Celite, the solvent was

removed under reduced pressure to yield an oily product **3**, which was used in the next step without further purification.

Yield: 98%; ^{1}H NMR ($CDCl_3$) δ = 2.76 (s, 6H), 4.29 (s, 2H), 7.20 (m, 2H), 7.43 (t, *J* = 7.6 Hz, 1H), 7.6 (dd, *J* = 7.6, 2.0 Hz, 1H), 10.2 (bs, 1H), 10.8 (bs, 1H); ^{13}C NMR ($CDCl_3$) δ = 42.1, 45.5, 76.6, 77.0, 77.4, 120.3, 124.7, 126.2, 131.3, 132.6, 152.8, 167.3, 173.4; HRMS (*m/z*): $[M + Na]^+$ calcd for $C_{11}H_{14}N_2O_3Na$, 245.0902; found, 245.0897.

Procedure B:

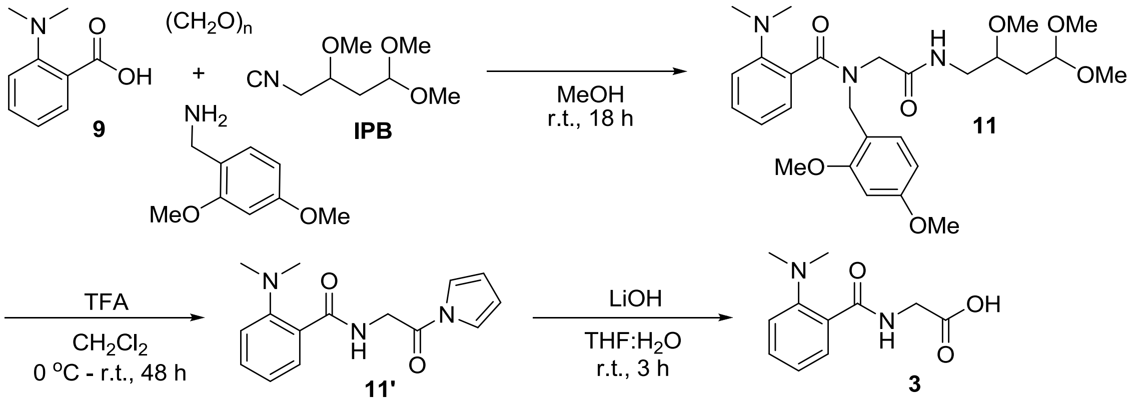

N-(2,4-Dimethoxybenzyl)-2-(dimethylamino)-*N*-(2-oxo-2-((2,4,4-trimethoxy-butyl)amino)ethyl)benzamide (**11**)

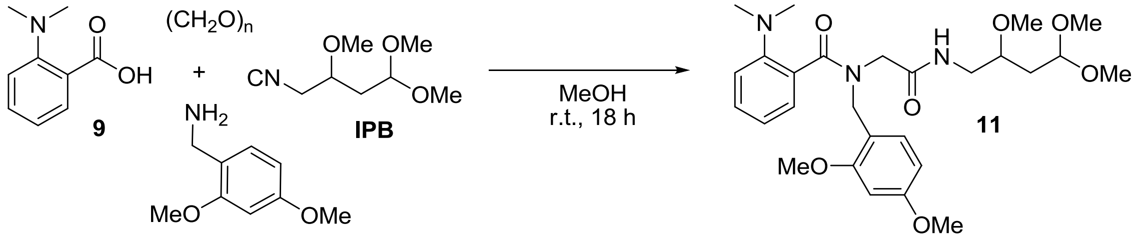

To a solution of 2,4-dimethoxybenzylamine (167 mg, 150 µL, 1.00 mmol) in methanol (10 mL) was added paraformaldehyde (50 mg, 1.67 mmol). This

suspension was stirred at room temperature for 2 h before 2-(dimethylamino)benzoic acid (165 mg, 1.00 mmol) and IPB (173 mg, 1.00 mmol) were added subsequently. After stirring for 18 h the solvent was removed in vacuo. The crude residue was purified by column chromatography (dichloromethane/ethyl acetate 2:3) to give **11** (180 mg) as a colorless oil.

Yield: 35%; ^{1}H NMR ($CDCl_3$): δ = 1.58–1.89 (m, 2H), 2.73, 2.81 (2s, 6H), 3.28–3.54 (m, 10H), 3.56–3.85 (m, 8H), 4.07–4.38 (2m, 2H), 4.48–4.93 (4m, 4H), 6.34–6.50 (2m, 2H), 6.61–6.88 (3m, 1H), 6.96–7.43 (5m, 4H); ^{13}C NMR ($CDCl_3$): δ = 29.6, 34.5, 35.0, 35.2, 41.2, 42.1, 42.7, 42.8, 43.1, 44.2, 44.3, 45.1, 45.1, 47.6, 47.7, 47.8, 52.1, 52.6, 52.9, 53.0, 55.0, 55.2, 56.9, 57.2, 57.4, 57.6, 76.0, 76.5, 77.1, 98.1, 98.3, 101.7, 101.9, 104.0, 104.2, 115.9, 116.7, 117.9, 118.8, 118.9, 122.3, 123.4, 123.5, 127.9, 128.3, 130.1, 130.1, 130.2, 130.2, 131.3, 131.5, 149.2, 150.4, 158.4, 158.7, 160.5, 160.7, 168.8, 169.0, 169.2, 171.0, 172.0, 172.1, 172.3; HRMS (*m*/*z*): $[M + H]^+$ calcd for $C_{27}H_{39}N_3O_7$, 518.2861; found 518.2861.

2-(Dimethylamino)-*N*-(2-oxo-2-(1*H*-pyrrol-1-yl)ethyl)benzamide (**11'**)

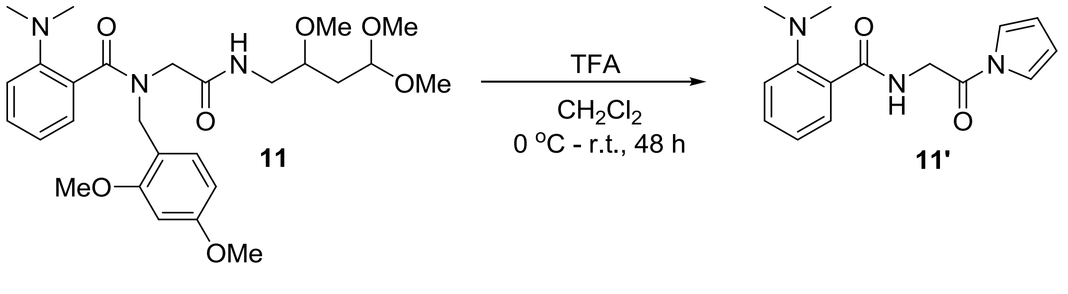

A solution of **11** (70 mg, 135 µmol) in dichloromethane (2.5 mL) was treated with trifluoroacetic acid (920 mg, 620 µL, 9.72 mmol) and the mixture was stirred at room temperature for 48 h. The deeply violet solution was treated with

saturated $NaHCO_3$ solution until the color disappeared completely. The resulting mixture was extracted with dichloromethane (6 × 60 mL) and the combined organic extracts were dried over Na_2SO_4. After filtration and evaporation of the solvent under reduced pressure the crude residue was purified by isocratic silica-gel column chromatography using (2:8) ethyl acetate:hexanes as eluents to give **11’** (15 mg) as a colorless solid.

Yield: 41%; ^{1}H NMR ($CDCl_3$, 400 MHz): δ = 2.81 (s, 6H), 4.87 (d, J = 4.9 Hz), 6.34–6.37 (m, 2H), 7.19–7.33 (2m, 2H), 7.38 (brs, 2H), 7.47 (ddd, J = 1.7, 7.4, 8.0 Hz, 1H), 8.20 (dd, J = 1.7, 7.8 Hz, 1H), 11.00 (br s, 1H); ^{13}C NMR ($CDCl_3$): 42.9, 45.5, 113.9, 118.7, 120.4, 124.5, 126.7, 131.4, 132.4, 153.1, 166.5, 166.9 ppm; HRMS (m/z): $[M + H]^+$ calcd for $C_{15}H_{17}N_3O_2$, 272.1394; found, 272.1396.

2-(2-(Dimethylamino)benzamido)acetic acid (**3**)

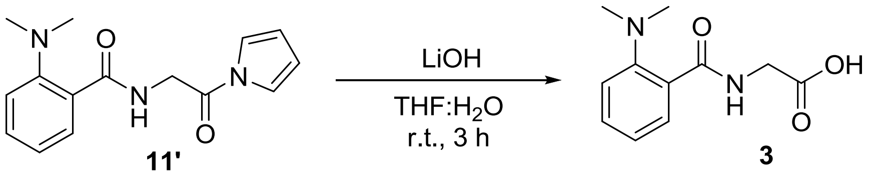

To a solution of **11’** (14.5 mg, 53.4 µmol) in a mixture of tetrahydrofurane/water 3:1 was added lithium hydroxide monohydrate (5.6 mg, 133.5 µmol). After strirring at room temperature for 3 h, the reaction mixture was evaporated to dryness. The crude residue was dissolved in brine (50 mL) and the pH was brought to pH 4–5 by dropwise addition of hydrochloric acid (2 M). The aqueous solution was extracted with dichloromethane (8 × 25 mL) and the combined organic layers were dried over Na_2SO_4. After filtration the solvent was removed in vacuo to yield **3** (6 mg) as a colorless, amorphous solid. Yield: 51%.

General procedure for the synthesis of viridic ester derivatives 13a–f by Ugi four-component reactions

To a stirred solution of an amine **12a**–**f** (1.1 mmol) in MeOH (5.0 mL), isobutyraldehyde (0.08 g, 0.1 mL, 1.1 mmol) was added and, after 1 h, followed by carboxylic acid **3** (0.22 g, 1.0 mmol) and isonitrile **4a** (0.16 g, 1.0 mmol). The mixture was stirred for 20 h, then the solvent was removed under reduced pressure and the crude material was purified by silica-gel column chromatography to afford the desired products. The details for the purification of each compound are given below.

Methyl 2-(2-(2-(2-(dimethylamino)benzamido)-*N*-methylacetamido)-3-methyl-butanamido)benzoate (**13a**)

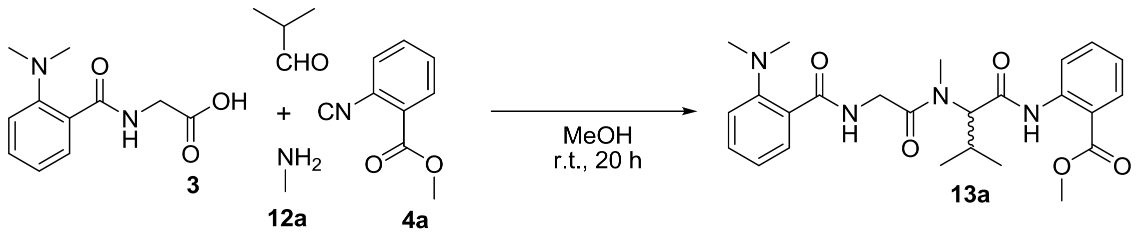

Purified by isocratic silica-gel column chromatography using (2:8) ethyl acetate:hexanes as eluents. Yield: 51%; ^{1}H NMR ($CDCl_3$) δ = 0.93 (d, *J* = 6.8 Hz, 3H), 1.10 (d, *J* = 6.8 Hz, 3H), 2.42 (m, 1H), 2.86 (s, 6H), 3.06 (s, 3H), 3.88 (s, 3H), 4.42 (d, *J* = 4.4 Hz, 2H), 4.99 (d, *J* = 10.8 Hz, 1H), 7.10 (m, 1H), 7.19 (m, 1H), 7.28 (m, 1H), 7.44 (m, 1H), 7.52 (m, 1H), 8.02 (dd, *J* = 8.0, 1.2 Hz, 1H), 8.06 (dd, *J* = 8.0, 1.6 Hz, 1H), 8.67 (d, *J* = 8.0 Hz, 1H), 10.45 (br, 1H),

11.28 (br, 1H); ^{13}C NMR ($CDCl_3$) δ = 18.7, 19.9, 26.3, 30.0, 42.3, 45.3, 52.3, 63.8, 104.9, 115.5, 119.9, 120.5, 122.8, 124.2, 127.1, 130.9, 131.1, 131.9, 134.4, 140.8, 166.5, 168.2, 168.6, 169.6.

Methyl 2-(2-(2-(2-(dimethylamino)benzamido)-*N*-propylacetamido)-3-methyl-butanamido)benzoate (**13b**)

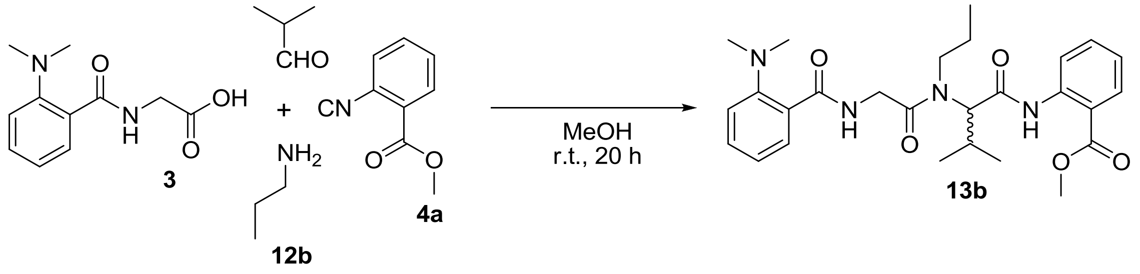

Purified by isocratic silica gel column chromatography using (2:8) ethyl acetate:hexanes as eluents. Yield: 55%; ^{1}H NMR ($CDCl_3$) δ = 0.86 (m, 6H), 1.01 (m, 3H), 1.06 (m, 2H), 2.43 (m, 1H), 2.75 (s, 6H), 3.26 (m, 2H), 3.79 (s, 3H), 3.87 (m, 2H), 4.4 (t, J = 4.8 Hz, 2H), 4.83 (d, J = 10.2 Hz, 1H), 7.01–7.17 (m, 3H), 7.34 (td, J = 8.0, 1.2 Hz, 1H), 7.47 (td, J = 8.0, 1.2 Hz, 1H), 7.97 (m,1H), 8.05 (dd, J = 8.0, 1.2 Hz, 1H), 8.58 (d, J = 8.0 Hz, 1H), 10.55 (bs,1H), 11.18 (s, 1H); ^{13}C NMR ($CDCl_3$) δ = 11.5, 18.8, 20.2, 22.9, 26.3, 27.3, 42.0, 45.2, 46.3, 52.3, 64.6, 67.7, 115.7, 119.8, 120.7, 122.8, 123.7, 126.9, 130.9, 131.2, 131.9, 134.4, 140.8, 153.0, 166.4, 167.9, 169.1, 169.8; HRMS (*m*/*z*): $[M + H]^+$ calcd for $C_{27}H_{36}N_4O_5$, 497.2764; found, 497.2758.

Methyl 2-(2-(*N*-benzyl-2-(2-(dimethylamino)benzamido)acetamido)-3-methyl-butanamido)benzoate (**13c**)

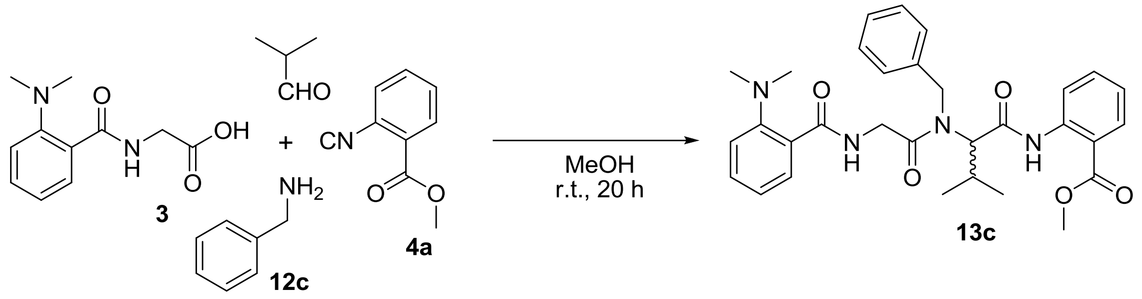

Purified by isocratic silica-gel column chromatography using (2:8) ethyl acetate:hexanes as eluents. Yield: 61%; ^{1}H NMR ($CDCl_3$) δ = 0.93 (m, 3H), 1.04 (m, 3H), 2.53 (m, 1H), 2.82 (s, 6H), 3.82–4.81 (m, 7H), 5.06 (d, *J* = 10.8 Hz, 1H), 6.86–7.42 (m, 10H), 8.00 (m, 2H), 8.27 (d, *J* = 8.4 Hz, 1H) 10.52 (bs, 1H), 11.28 (s, H); ^{13}C NMR ($CDCl_3$) δ = 18.8, 19.8, 42.6, 45.4, 47.4, 52.4, 64.8, 115.7, 120.75, 122.9, 126.1, 127.3, 127.4, 127.8, 127.4, 128.0, 128.7, 130.8, 131.2, 132.0, 134.3, 136.3, 140.6, 166.4, 168.2, 168.3, 170.8; HRMS (*m*/*z*): $[M + Na]^+$ calcd for $C_{31}H_{36}N_4O_5Na$, 567.2583; found, 567.2580.

Methyl 2-(2-(2-(2-(dimethylamino)benzamido)-*N*-(4-fluorobenzyl)acetamido)-3-methylbutanamido)benzoate (**13d**)

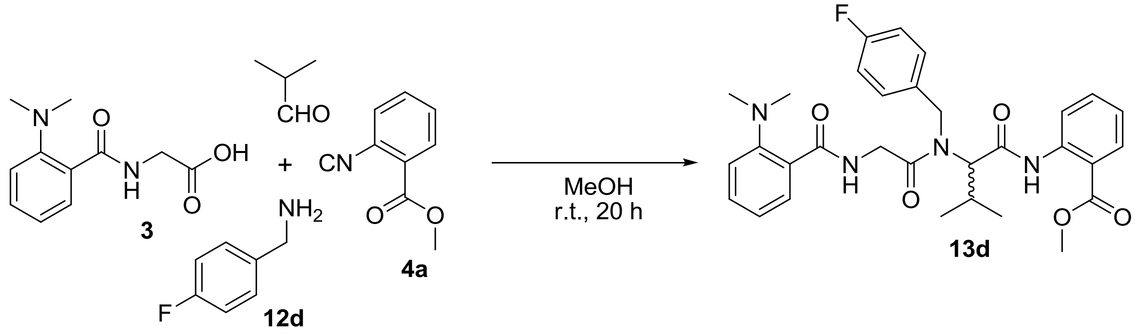

Purified by isocratic silica-gel column chromatography using (2:8) ethyl acetate:hexanes as eluents. Yield: 58%; ^{1}H NMR ($CDCl_3$) δ = 0.94 (m, 3H), 1.07 (m, 3H), 2.57 (m, 1H), 2.80 (s, 6H), 3.82–4.53 (m, 5H), 4.74 (m, 2H), 5.07 (d, *J* = 10.8 Hz, 1H), 6.65 (t, *J* = 8.8 Hz, 1H), 6.83 (t, *J* = 8.8 Hz, 1H), 7.04–7.23 (m, 5H), 7.39–7.47 (m, 2H), 8.02 (dd, *J* = 8.0, 1.2 Hz, 1H), 8.08 (dd, *J* = 8.0, 1.2 Hz, 1H), 8.30 (m, 1H), 10.60 (bs, 1H), 11.30 (bs, 1H); ^{13}C NMR ($CDCl_3$) δ = 18.7, 19.8, 20.2, 26.8, 29.7, 42.5, 45.3, 46.7, 52.4, 64.6, 67.6, 114.7, 115.0, 115.7, 119.8, 120.1, 120.6, 123.0, 123.8, 127.0, 127.8, 129.9, 130.6, 130.9, 131.4, 132.0, 133.1, 134.4, 140.5, 153.0, 160.7, 163.2, 166.5, 168.2, 170.7; HRMS (*m*/*z*): $[M + Na]^+$ calcd for $C_{31}H_{35}FN_4O_5Na$, 585.2489; found, 585.2483.

Methyl 2-(2-(*N*-(4-chlorobenzyl)-2-(2-(dimethylamino)benzamido)acetamido)-3-methylbutanamido)benzoate (**13e**)

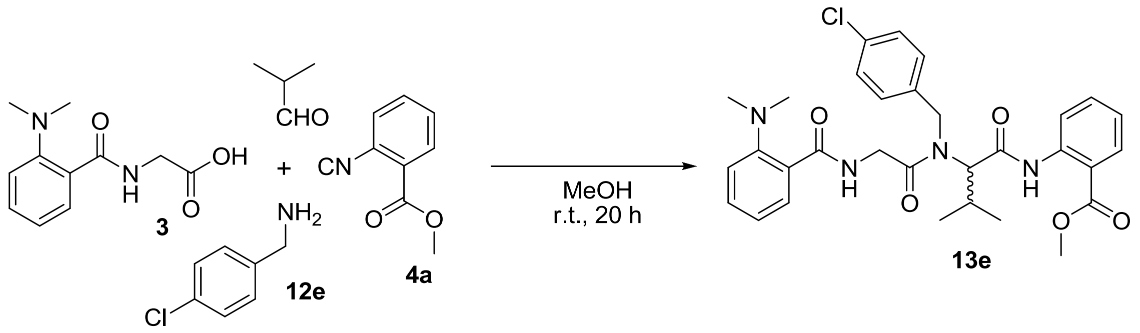

Purified by isocratic silica-gel column chromatography using (2:8) ethyl acetate:hexanes as eluents. Yield: 70%; ^{1}H NMR ($CDCl_3$) δ = 0.95 (m, 3H), 1.06 (m, 3H), 2.57 (m, 1H), 2.80 (s, 6H), 3.77–5.11 (m, 8H), 6.91 (d, *J* = 8.4 Hz, 1H), 7.04–7.25 (m, 6H), 7.38–7.47 (m, 2H), 8.04 (dd, *J* = 8.0, 1.2 Hz, 1H), 8.08 (dd, *J* = 8.0, 1.2 Hz, 1H), 8.27 (d, *J* = 8.4 Hz, 1H), 10.61 (bt, *J* = 4.4 Hz, 1H), 11.31 (s, 1H); ^{13}C NMR ($CDCl_3$) δ = 18.6, 19.7, 20.2, 26.7, 29.7, 42.5, 45.3,

45.31, 46.7, 52.4, 61.0, 64.5, 67.5, 115.0, 115.6, 119.8, 120.5, 123.0, 123.9, 126.9, 127.4, 127.9, 128.0, 129.5, 130.8, 131.3, 132.0, 132.5, 134.4, 134.5, 135.1, 140.4, 152.9, 166.4, 168.1, 169.8, 170.6; HRMS (*m*/*z*): $[M + H]^+$ calcd for $C_{31}H_{35}ClN_4O_5$, 579.2374; found, 579.2368.

Methyl 2-(2-(2-(2-(dimethylamino)benzamido)-*N*-phenethylacetamido)-3-methylbutanamido)benzoate (**13f**)

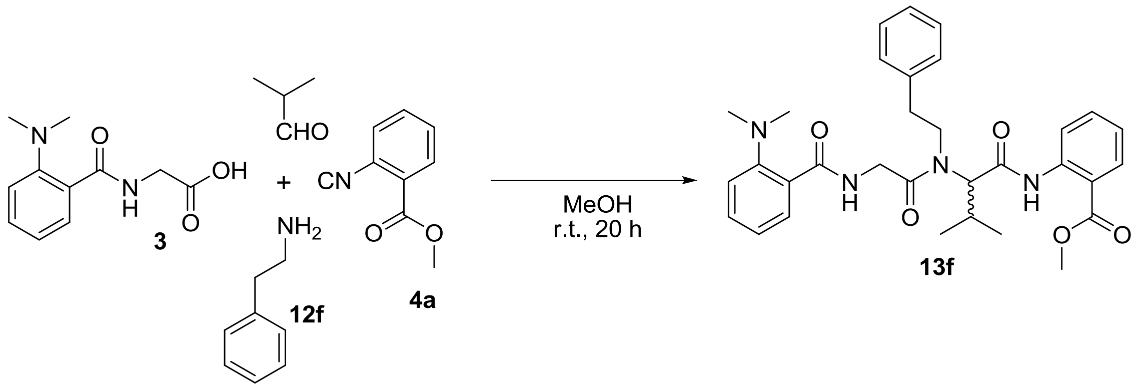

Purified by isocratic silica-gel column chromatography using (2:8) ethyl acetate:hexanes as eluents. Yield: 52%; ^{1}H NMR ($CDCl_3$) δ = 0.92 (m, 3H), 1.09 (m, 3H), 2.54 (m, 1H), 2.86 (s, 6H), 2.90 (m, 2H), 3.59 (m, 2H), 3.85 (s, 3H), 4.51 (m, 2H), 4.97 (d, *J* = 10.8 Hz, 1H), 7.04–7.27 (m, 8H), 7.40 (td, *J* = 8.0, 1.2 Hz, 1H), 7.52 (td, *J* = 8.0, 1.2 Hz, 1H), 8.00 (m, 1H), 8.14 (m, 1H), 8.71 (d, *J* = 8.0 Hz, 1H) 10.66 (t, *J* = 4.4 Hz, 1H), 11.33 (s, 1H); ^{13}C NMR ($CDCl_3$) δ = 18.6, 20.1, 26.4, 36.0, 42.0, 45.3, 46.2, 52.3, 64.4, 67.7, 115.7, 120.3, 120.7, 122.9, 123.2, 123.9, 126.9, 128.4, 128.4, 128.8, 130.9, 131.0, 132.0, 134.5, 137.8, 139.2, 140.8, 153.1, 166.5, 168.1, 169.2, 169.9; HRMS (*m*/*z*): $[M + Na]^+$ calcd for $C_{32}H_{38}N_4O_5Na$, 581.2740; found, 581.2734.

General procedure for the synthesis of racemic viridic acid (±)-1 and higher *N*-alkyl derivatives 14b–f

To a stirred solution of compound **13a–f** (0.4 mmol) in THF:H_2O (6:4; 10 mL), LiOH·H_2O (43 mg, 1.0 mmol) was added, then after 8 h water (15 mL), and the resulting mixture was carefully acidified with aqueous citric acid solution (30% w/w) to pH 7.0. The resulting suspension was saturated with brine (50 mL) and was extracted with ethyl acetate (4 × 20 mL). The organic layer was dried over Na_2SO_4 and distilled off under reduced pressure. The details for purification of each compound are given below.

(±)-Viridic acid, [2-(2-(2-(2-(dimethylamino)benzamido)-*N*-methylacetamido)-3-methylbutanamido)benzoic acid] (**1**)

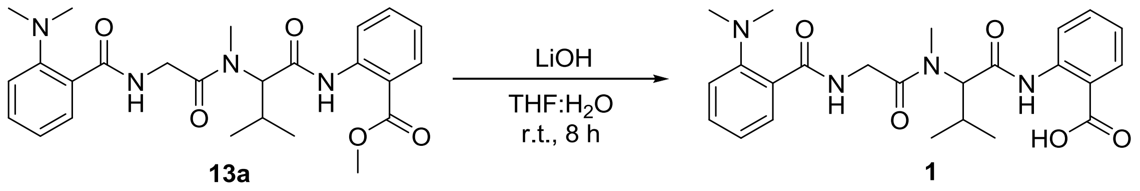

Yield: 83%; ^{1}H NMR and ^{13}C NMR spectra were identical to compound (−)-**1** described above.

2-(2-(2-(2-(Dimethylamino)benzamido)-*N*-propylacetamido)-3-methylbutan-amido)benzoic acid (**14b**)

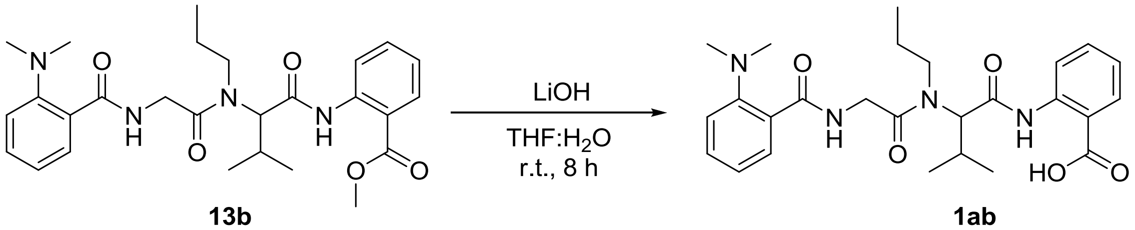

13b **1ab**

Purified by silica-gel column chromatography using a gradient of (99:1 → 95:5) dichloromethane:methanol as eluent. Yield: 83%; ^{1}H NMR (DMSO-d_6) δ = 0.73 (t, J = 7.6 Hz, 3H), 0.82–1.00 (m, 6H), 1.53 (m, 2H), 2.34 (m, 1H), 2.69 and 2.72 (s, 6H), 3.33 (m, 2H), 3.88–4.62 (m, 3H), 7.00–7.48 (m, 5H), 7.78–7.93 (m, 1H), 8.02 (m, 1H), 8.46 (m, 1H), 9.76 (bt, J = 5.2 Hz, 1H), 10.08 (bs, 1H); ^{13}C NMR (DMSO-d_6) δ = 11.3, 11.4, 18.2, 18.9, 19.5, 20.4, 20.6, 22.5, 26.5, 26.9, 41.2, 41.6, 44.6, 44.8, 46.1, 64.4, 66.9, 118.5, 119.0, 119.2, 119.6, 126.6, 130.5, 130.8, 131.2, 131.7, 131.9, 140.4, 152.2, 152.4, 166.0, 166.2, 167.1, 168.35, 168.5, 169.3, 170.6, 170.8; HRMS (*m*/*z*): $[M + H]^+$ calcd for $C_{26}H_{34}N_4O_5$, 483.2607; found, 483.2602.

2-(2-(*N*-Benzyl-2-(2-(dimethylamino)benzamido)acetamido)-3-methylbutan-amido)benzoic acid (**14c**)

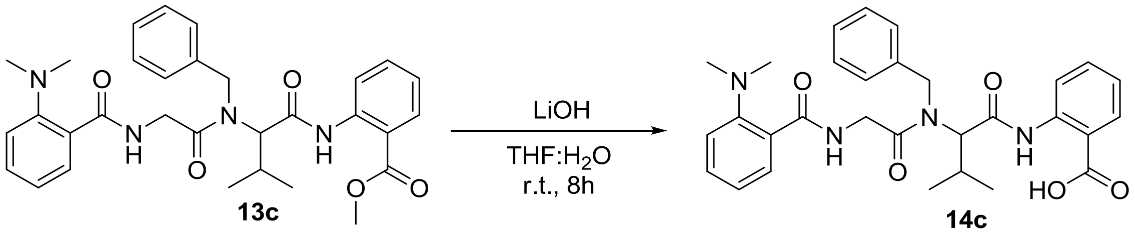

13c **14c**

Purified by silica-gel column chromatography using a gradient of (99:1 → 95:5) dichloromethane:methanol as eluents. Yield: 88%; ^{1}H NMR (DMSO-d_6) δ = 0.84–1.00 (m, 6H), 2.35 (m, 1H), 2.68 and 2.71 (s, 6H), 3.74–4.86 (m, 5H), 7.00–7.46 (m, 10H), 7.72 and 7.89 (d, *J* = 7.2 Hz, 1H), 8.02 (m, 1H), 8.21 (m, 1H), 9.70 (bs, 1H), 9.98 (bs, 1H); ^{13}C NMR (DMSO-d_6) δ = 18.8, 19.2, 19.9, 20.2, 27.3, 27.7, 42.3, 44.9, 45.1, 46.0, 47.1, 55.4, 64.7, 67.8, 119.2, 119.6, 119.8, 122.6, 127.1, 127.2, 127.4, 128.1, 128.9, 130.8, 131.1, 131.5, 132.1. 138.3, 138.5, 140.6, 152.5, 152.7, 166.6, 167.1, 168.2, 169.9, 170.7, 171.4; HRMS (*m*/*z*): [M + Na]$^+$ calcd for $C_{30}H_{34}N_4O_5Na$, 553.2427; found, 553.2421.

2-(2-(2-(2-(Dimethylamino)benzamido)-*N*-(4-fluorobenzyl)acetamido)-3-methyl-butanamido)benzoic acid (**14d**)

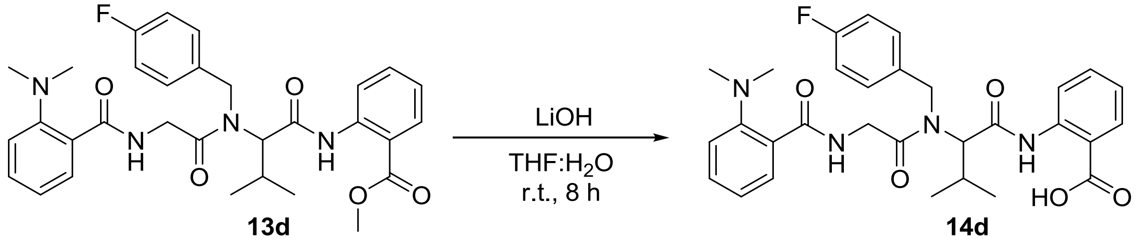

Purified by silica-gel column chromatography using a gradient of (99:1 → 95:5) dichloromethane:methanol as eluents. Yield: 90%; ^{1}H NMR (DMSO-d_6) δ = 0.83–1.00 (m, 6H), 2.34 (m, 1H), 2.69 and 2.72 (s, 6H), 3.76–4.85 (m, 5H), 6.82 (t, *J* = 9.2 Hz, 1H) 6.95–7.27 (m, 7H), 7.39 (m, 1H), 7.72 and 7.89 (dd, *J* = 8.0, 1.6 Hz, 1H), 7.92 (m, 1H), 8.16 (m, 1H), 9.70 (bt, *J* = 4.8 Hz, 1H), 9.98 (bs, 1H); ^{13}C NMR (DMSO-d_6) δ = 18.3, 18.8, 19.5, 19.8, 26.7, 27.2, 41.7, 44.5, 44.6, 64.3, 67.3, 114.2, 114.4, 115.0, 115.2, 118.4, 118.9, 119.1, 119.4, 122.0, 122.2, 122.4, 126.6, 128.0, 129.0, 130.4, 130.7, 131.1, 131.6, 131.8, 134.1, 140.0, 141.1, 152.1, 152.3, 159.5, 159.8, 161.9, 162.2, 166.3, 166.6, 167.7, 169.5,

170.2, 170.6, 171.1; HRMS (*m*/*z*): [M + Na]$^+$ calcd for $C_{30}H_{33}FN_4O_5Na$, 571.2327; found, 571.2333.

2-(2-(*N*-(4-Chlorobenzyl)-2-(2-(dimethylamino)benzamido)acetamido)-3-methyl-butanamido)benzoic acid (**14e**)

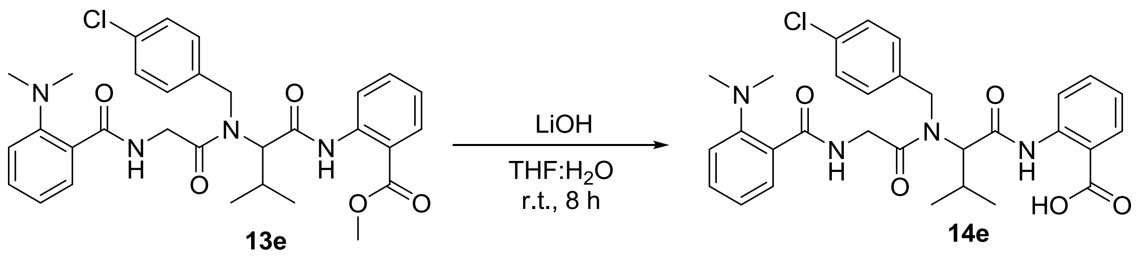

Purified by silica-gel column chromatography using a gradient of (99:1 → 95:5) dichloromethane:methanol as eluents. Yield: 91%; ^{1}H NMR (DMSO-d_6) δ = 0.83–1.00 (m, 6H), 2.34 (m, 1H), 2.69 and 2.71 (s, 6H), 3.75–4.88 (m, 5H), 6.98–7.32 (m, 8H), 7.42 (m, 1H), 7.71 and 7.87 (dd, *J* = 8.0, 1.6 Hz, 1H), 7.98 (m, 1H), 8.12 (m, 1H), 9.70 (bt, *J* = 4.8 Hz, 1H), 9.93 (bs, 1H); ^{13}C NMR (DMSO-d_6) δ = 18.3, 18.7, 19.4, 19.7, 26.7, 27.1, 41.7, 44.5, 46.0, 64.2, 67.0, 119.1, 122.2, 126.7, 127.5, 127.8, 128.3, 128.9, 130.3, 130.8, 131.1, 131.7, 136.9, 140.0, 152.1, 152.2, 166.3, 166.1, 167.6, 169.6, 170.2; HRMS (*m*/*z*): [M + Na]$^+$ calcd for $C_{30}H_{33}ClN_4O_5Na$, 587.2037; found, 587.2032.

2-(2-(2-(2-(Dimethylamino)benzamido)-*N*-phenethylacetamido)-3-methylbutan-amido)benzoic acid (**14f**)

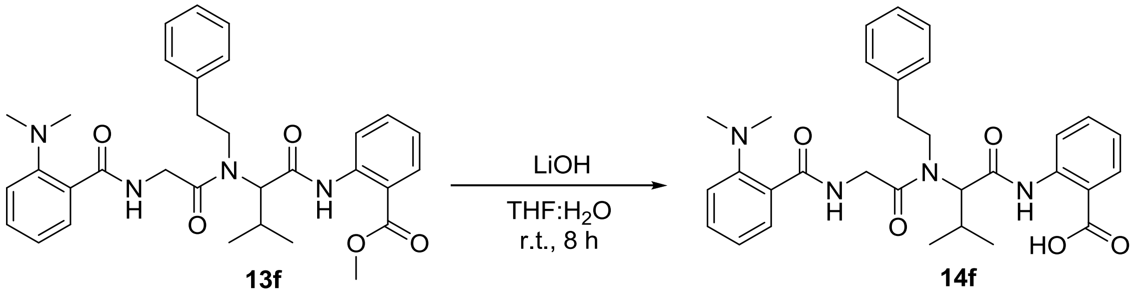

13f **14f**

Purified by silica-gel column chromatography using a gradient of (99:1 → 95:5) dichloromethane:methanol as eluents. Yield: 81%; ^{1}H NMR (DMSO-d_6) δ = 0.83–1.03 (m, 6H), 2.41 (m, 1H), 2.70 and 2.75 (s, 6H), 2.87 (m, 2H), 3.22–4.73 (m, 5H), 7.00–7.49 (m, 10H), 7.42 (m, 1H), 7.82 and 7.93 (dd, *J* = 8.0, 1.6 Hz, 1H), 8.06 (m, 1H), 8.53 (t, *J* = 7.6 Hz, 1H), 9.81 (bs, 1H), 10.01 (bs, 1H); ^{13}C NMR (DMSO-d_6) δ = 18.7, 19.2, 19.8, 20.7, 26.9, 27.4, 33.9, 35.8, 41.7, 42.0, 45.0, 45.2, 46.4, 64.8, 67.2, 118.9, 119.3, 119.7, 119.9, 122.6, 122.8, 123.0, 126.6, 126.9, 127.1, 128.9, 129.0, 129.1, 130.9, 131.2, 131.7, 132.2, 132.3, 138.9, 139.6, 140.8, 152.6, 152.9, 166.7, 167.4, 168.8, 168.9, 169.9, 171.2, 171.4; HRMS (*m*/*z*): $[M + H]^+$ calcd for $C_{31}H_{35}N_4O_5$, 545.2764; found, 545.2758.

(−)-Menthyl 2-isocyanobenzoate (**4b**)

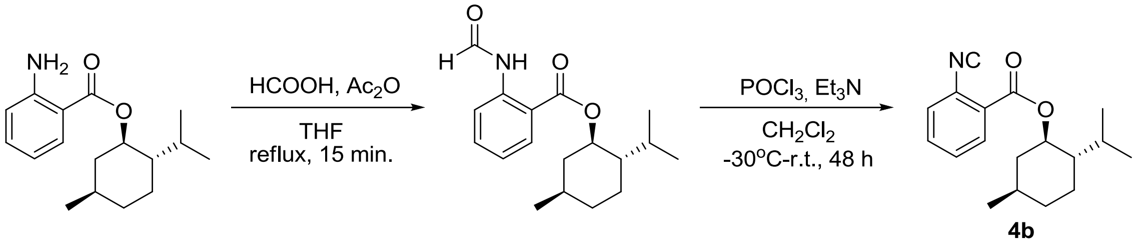

4b

2-Trifluoroacetamidobenzoic acid [8]

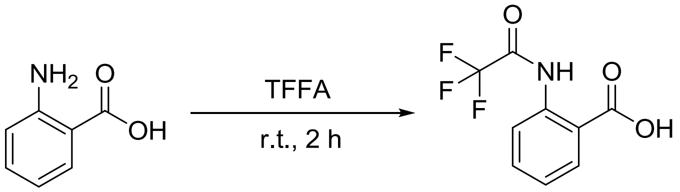

To externally cooled trifluoroacetic anhydride (122.8 mL, 184.8 g, 0.88 mol) was added anthranilic acid (60.5 g, 0.44 mol) in portions over 15 min. The resulting suspension was stirred for 2 h at room temperature. During this time, a clear solution formed, which was poured into a mixture of ice and water (1:1, 400 mL) under vigorous stirring. The formed precipitate was filtered off and washed with ice-cold water (1000 mL) to remove free trifluoroacetic acid. After drying in vacuo, the crude product was recrystallized from a mixture of ethanol (220 mL) and water (260 mL) to give desired compound **1** (90.3 g, 88%) as yellowish needles.

Yield: 88%; ^{1}H NMR (CD_3OD, 400 MHz) δ = 7.23–7.31 (*m*, 1H), 7.58–7.66 (m, 1H), 8.12 (dd, 1H, J = 1.4, 7.9 Hz), 8.54 (d, 1H, J = 8.4 Hz); ^{13}C NMR (CD_3OD, 100 MHz): δ = 117.2 (q, $^{1}J_{C,F}$ = 287.9 Hz), 118.2, 121.3, 125.9, 132.7, 135.6, 140.1, 156.1 (q, $^{2}J_{C,F}$ = 37.3 Hz), 171.3.

(−)-Menthyl 2-aminobenzoate

Procedure A

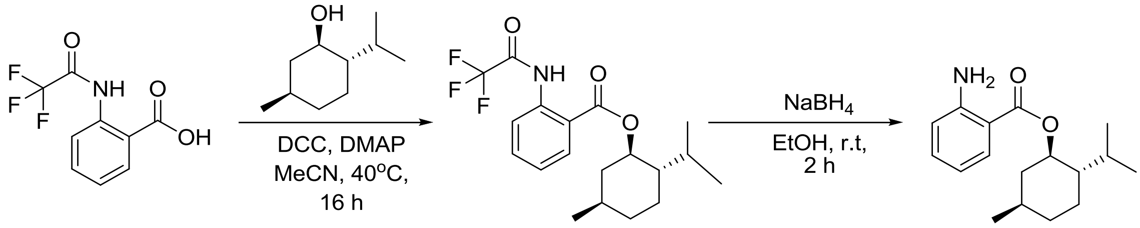

(−)-Menthol (1.56 g, 10.0 mmol), 2-trifluoroacetamidobenzoic acid (4.66 g, 20.0 mmol) and DMAP (122 mg, 1.0 mmol) were dissolved in dry acetonitrile (50 mL) at room temperature. To the resulting solution was added *N*,*N*'-dicyclohexylcarbodiimide (4.13 g, 20.0 mmol). After this addition the mixture was warmed to 40 °C and it was stirred overnight under an inert gas atmosphere. After cooling to room temperature the reaction mixture was filtered and the residue was washed with acetonitrile (250 mL). Afterwards the solvent was completely distilled off under reduced pressure. The crude residue was dissolved in dichloromethane (100 mL) and the resulting organic phase was washed with half concentrated aqueous $NaHCO_3$ solution (100 mL) and subsequently with brine (100 mL). After drying over Na_2SO_4 and filtration the solvent was removed in vacuo to give 5.59 g of an oily product, which was used for the next step without further purification. The crude residue (5.59 g, 10.0 mmol) was dissolved in absolute ethanol (60 mL). To this solution was added sodium borohydride (1.89 g, 50.0 mmol) in portions over 20 min at room temperature. After complete addition of the hydride the mixture was stirred for 2 h at room temperature before it was quenched with a mixture of water (50 mL)

and methanol (5 mL). The resulting solution was evaporated under reduced pressure to approximately 1/10 in volume, then water (100 mL) was added. Subsequently, the aqueous layer was extracted with ethyl acetate (3 × 100 mL). The combined extracts were washed with brine (100 mL) and were dried over Na_2SO_4. After filtration, solvents were removed in vacuo and the crude residue was purified by isocratic silica-gel column chromatography using (95:5) ethyl acetate:hexanes as eluent to give compound **2** (2.21 g) as a colorless oil.

Yield: 80%; $[\alpha]_D^{22}$ −110.2 (*c* 1.12; $CHCl_3$); ^1H NMR ($CDCl_3$) δ = 0.79 (d, *J* = 7.0 Hz, 3H), 0.88–0.95 (m, 7H), 1.03–1.20 (m, 2H), 1.48–1.62 (m, 2H), 1.67–1.77 (m, 2H), 1.92–2.03 (m, 1H), 2.07–2.15 (m, 1H), 4.89 (ddd, *J* = 4.4, 10.9, 10.9 Hz, 1H), 5.74 (br s, 2H), 6.61–6.69 (m, 2H), 7.22–7.29 (m, 1H), 7.84–7.90 (m, 1H); ^{13}C NMR ($CDCl_3$) δ = 16.5, 20.8, 22.1, 23.6, 26.4, 31.5, 34.3, 41.1, 47.2, 74.0, 111.4, 116.2, 116.7, 131.2, 133.8, 150.5, 167.7; HRMS (*m*/*z*): $[M + H]^+$ calcd for $C_{17}H_{26}NO_2$, 276.1964; found, 276.1958.

Procedure B:

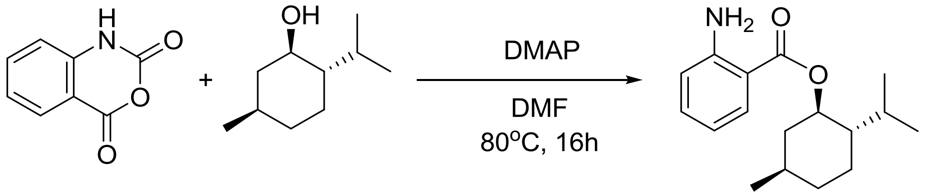

To a stirred solution of isatoic anhydride (1.6 g, 10 mmol) and DMAP (0.12 g, 1 mmol) in DMF (10 mL) was added (−)-menthol (1.87 g, 12 mmol) under N_2 atmosphere. The mixture was stirred at 80 °C for 16 h. The reaction was cooled to room temperature and partitioned between water (50 mL) and ethyl acetate

(50 mL). The aqueous phase was further extracted with ethyl acetate (2 × 20 mL). The organic layers were combined, washed with brine (2 × 50 mL) and dried over Na_2SO_4. The solvent was evaporated under reduced pressure and the crude residue was purified by isocratic silica-gel column chromatography using (5:95) ethyl acetate:hexanes to give the title compound (1.4 g) as a colorless oil. Yield: 51%.

(−)-Menthyl 2-formamidobenzoate

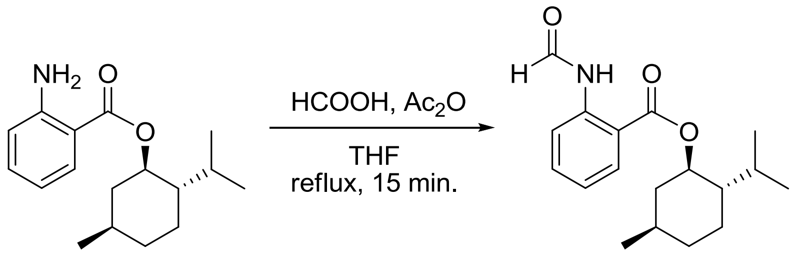

To externally cooled formic acid (4.24 mL, 5.18 g, 112.5 mmol) was added acetic anhydride (7.08 mL, 7.66 g, 75.0 mmol) at room temperature. This mixture was heated to 60 °C for 150 min and afterwards cooled to room temperature again. Subsequently, the preformed anhydride was added dropwise to a solution of compound (−)-menthyl 2-aminobenzoate (2.06 g, 7.5 mmol) in anhydrous tetrahydrofurane (100 mL) under reflux in N_2. After complete addition the reaction was heated under reflux for 15 min, and then all solvents were completely removed under reduced pressure. The residue was dissolved in ethyl acetate (100 mL) and the organic layer was washed with saturated aqueous $NaHCO_3$ solution (2 × 100 mL) and brine (100 mL). The organic phase was dried over Na_2SO_4 and evaporated after filtration. This

procedure gave (−)-menthyl 2-formamidobenzoate (2.27 g) as a yellow oil, which appears on the NMR-timescale as a mixture of s-*cis*/s-*trans* isomers.

Yield: quant; $[\alpha]_D^{22}$ −131.0 (*c* 0.81; $CHCl_3$); ^{1}H NMR ($CDCl_3$): δ = 0.79 (d, *J* = 6.9 Hz, 3H), 0.87–1.03 (m, 7H), 1.05–1.21 (m, 2H), 1.50–1.65 (m, 2H), 1.78–1.82 (m, 2H), 1.86–2.00 (m, 1H), 2.06–2.15 (m, 1H), 4.94 (ddd, *J* = 4.4, 10.9, 10.9 Hz, 1H), 7.10–7.17 (m, 1H), 7.52–7.59 (m, 1H), 8.02–8.08 (m, 1H), 8.48–8.55 (m, 1H), 8.93–9.00, 8.68–8.76 (2m, 1H), 10.59, 11.13 (2s, 1H); ^{13}C NMR ($CDCl_3$): δ = 16.4, 20.7, 22.0, 23.5, 26.5, 31.5, 34.2, 40.8, 47.1, 75.6, 115.7, 121.2, 123.1, 130.8, 134.5, 140.5, 159.5, 167.6; HRMS (*m*/*z*): $[M - H]^-$ calcd for $C_{18}H_{24}NO_3$, 302.1757; found, 302.1762.

(−)-Menthyl 2-isocyanobenzoate (**4b**)

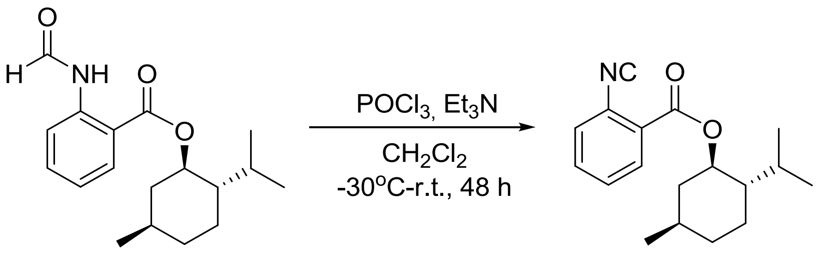

4b

(−)-Menthyl 2-formamidobenzoate (2.22 g, 7.32 mmol) and triethylamine (2.57 mL, 1.85 g, 18.3 mmol) in anhydrous dichloromethane (75 mL) were cooled to −20 °C. Subsequently, phosphoryl chloride (0.94 mL, 1.57 g, 10.25 mmol) was added, while the temperature was maintained between −30 °C and −20 °C. After complete addition, the reaction was stirred for 30 min at −30 °C and then warmed to room temperature. The mixture was stirred for 48 h at ambient temperature. It was quenched with a solution of potassium

hydroxide (5.2 g, 78.8 mmol) in water (110 mL) cooled to 0 °C. The resulting emulsion was vigorously stirred for 10 min at 0 °C before the layers were left to separate. Afterwards the organic phase was washed with diluted (5% w/w) sodium chloride solution (2 × 100 mL) and brine (1 × 100 mL) before it was dried over Na_2SO_4 and filtered. After evaporation of the solvents in vacuo the residue was purified by isocratic silica-gel column chromatography using (1:9) ethyl acetate:hexanes as eluents, and **4b** (1.69 g) was isolated as a light-orange oil.

Yield: 81%; $[\alpha]_D^{22}$ −88.1° (*c* 7.79; $CHCl_3$); ^{1}H NMR ($CDCl_3$) δ = 0.81 (d, *J* = 7.0 Hz, 1H), 0.88–1.00 (m, 7H), 1.06–1.27 (m, 2H), 1.48–1.67 (m, 2H), 1.79–1.68 (m, 2H), 2.07–1.94 (m, 1H), 2.21–2.12 (m, 1H), 5.02 (ddd, *J* = 4.4, 10.9, 10.9 Hz, 1H), 7.51–7.45 (m, 2H), 7.59–7.53 (m, 1H), 8.04–7.99 (m, 1H); ^{13}C NMR ($CDCl_3$): δ 16.2, 20.8, 22.0, 23.3, 26.3, 31.5, 34.1, 40.7, 46.8, 76.3, 125.4, 127.5, 128.9, 129.1, 131.3, 132.8, 163.3, 169.5; HRMS (*m*/*z*): $[M + Na]^+$ calcd for $C_{18}H_{23}NO_2Na$, 308.1621; found, 308.1621.

(−)-Menthyl 2-(2-(2-(2-(dimethylamino)benzamido)-*N*-methylacetamido)-3-methylbutanamido)benzoate (**13g**, mixture of diastereomers)

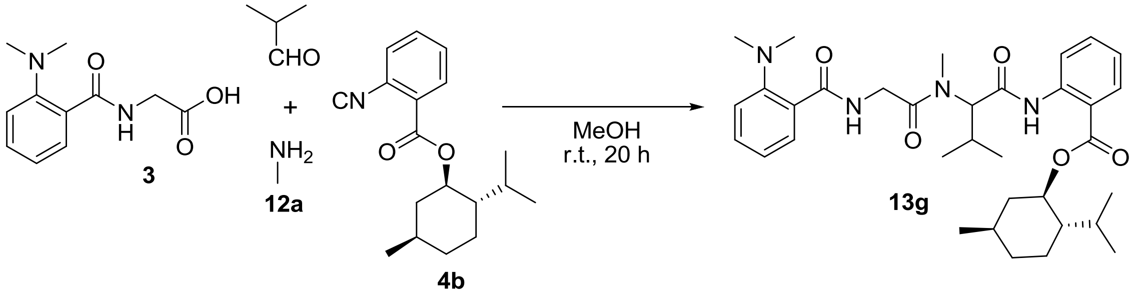

To a stirred solution of methylamine hydrochloride (0.14 g, 1.1 mmol) in MeOH (5.0 mL) were added triethylamine (0.11 g, 0.14 mL, 1.1 mmol) and isobutyraldehyde (0.1 g, 0.13 mL, 1.1 mmol). After stirring for 1 h, compound **3** (0.23 g, 1.0 mmol) and isonitrile **4b** (0.29 g, 1.0 mmol) were added. The reaction mixture was stirred for 20 h. The solvent was removed under reduced pressure, and the crude material was purified by silica-gel column chromatography using a gradient of (2:8 → 5:5) ethyl acetate:hexanes as eluents to afford 0.31 g of **13g** as a yellow oil.

Yield: 52%; ^{1}H NMR ($CDCl_3$) δ = 0.72 (m, 3H), 0.85–0.94 (m, 9H), 1.10 (m, 3H), 1.26–1.51 (m, 4H), 1.67 (m,1H), 1.71 (m, 1H), 2.05 (m, 1H), 2.40 (m, 1H), 2.81 (s, 1H), 3.05 and 3.07 (s, 1H), 4.41 (s, 2H), 4.90 (m, 1H), 5.01 (m, 1H), 7.43 (td, *J* = 8.0, 1.2 Hz, 1H), 7.54 (td, *J* = 8.0, 1.2 Hz, 1H), 8.03 (m, 1H), 8.13 (dd, *J* = 8.0, 1.2 Hz, 1H), 8.64 (d, *J* = 8.0 Hz, 1H), 10.63 (bs, 1H), 11.42 (s, 1H); ^{13}C NMR ($CDCl_3$) δ = 16.4, 18.8, 19.9, 20.7, 22.0, 23.5, 26.4, 26.5, 30.1, 31.4, 34.2, 40.7, 42.4, 45.3, 47.1, 63.8, 75.3, 116.1, 119.7, 120.5, 122.7, 123.7, 126.9, 130.7, 131.3, 131.9, 134.2, 141.0, 153.0, 166.3, 167.3, 168.7, 168.8, 169.5; HRMS (*m*/*z*): $[M + Na]^+$ calcd for $C_{34}H_{48}N_4O_5Na$, 615.3522; found, 615.3519.

(±)-Viridic acid, [2-(2-(2-(2-(dimethylamino)benzamido)-*N*-methylacetamido)-3-methylbutanamido)benzoic acid] (**1**)

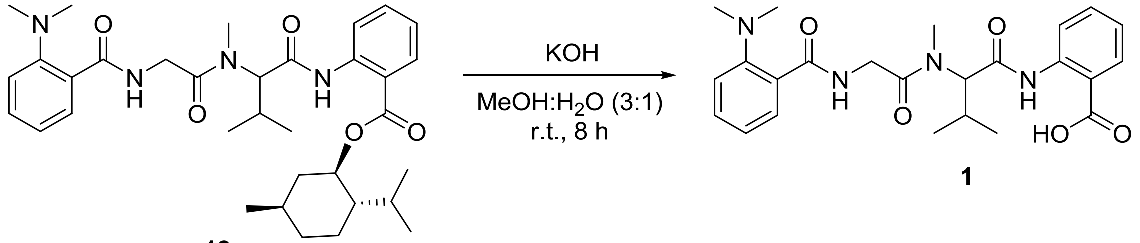

13g

1

To a stirred solution of compound **13g** (0.3 g, 0.5 mmol) in MeOH:H_2O (3:1) (5.0 mL), KOH (60 mg, 1.0 mmol) was added and the contents were stirred for 8 h. After this time water (15 mL) was added, and the resulting mixture was carefully acidified with citric acid solution (30% w/w) to pH 7.0 The resulting suspension was saturated with brine (50 mL) and was extracted with ethyl acetate (4 × 20 mL). The organic layer was dried over Na_2SO_4 and the solvent was removed under reduced pressure. The crude material was purified by silica-gel column chromatography using a gradient of (1:9 → 7:3) methanol:dichloromethane as eluents to afford 0.22 g of (±)-**1** as a colorless crystals.

Yield: 70%; ^{1}H NMR and ^{13}C NMR spectra were identical to compound (−)-**1** described above.

Biological assays

The *Aliivibrio fischeri* assay is based on the measurement of the inhibition of bacterial luminescence against a negative control. Bacteria were pregrown on a saline BOSS medium (3% NaCl w/w) whereby at a certain population density, bacterial luminescence will start. The bacterial suspension was diluted, distributed into 96-well microtiter plates (mtp) and the respective compounds were applied as a concentration series as solutions in DMSO/medium (2% v/v) and mixed. The luminescence of bacteria treated with the respective compounds was measured after 24 h incubation at 23 °C in the dark, in relation to controls of untreated bacteria.

References

[1] Neves Filho, R. A. W.; Stark, S. S.; Morejon, M. C.; Westermann, B.; Wessjohann, L. A. *Tetrahedron Lett.* **2012**, *53*, 5360-5363.

[2] Venuti, M. C. *Synthesis* **1982**, 266-268.

[3] Malkov, A. V.; Stoncius, S.; Mac Dougall, K. N.; Mariani, A.; McGeoch, G. D.; Kocovsky, P. *Tetrahedron* **2006**, *62*, 264-284.

[4] Malkov, A. V.; Vrankova, K.; Cerny, M.; Kocovsky, P. *J. Org. Chem.* **2009**, *74*, 8425-8427.

[5] Kobayashi, K.; Nakashima, T.; Mano, M.; Morikawa, O.; Konishi, H. *Chem. Lett.* **2001**, 602-603.

[6] Mauger, A. B.; Rzeszota, W. J.; Ford, R. A. *Org. Magn. Resonance* **1973**, *5*, 231-234.

[7] Holzapfel, C. W.; Koekemoer, J. M.; Vandyk, M. S. *South Afr. J. Chem.* **1986**, *39*, 75-80.

[8] Barker, D.; Brimble, M. A.; McLeod, M. D. *Tetrahedron* **2004**, *60*, 5953-5963.
