# Supplementary material for: The multicomponent approach to N-methyl peptides: total synthesis of antibacterial (–)-viridic acid and analogues
Source: Beilstein J Org Chem. 2012 Nov 28;8:2085–90. doi: 10.3762/bjoc.8.234 (PMC3511043; doi:10.3762/bjoc.8.234)
Supplement: File 2 — Figures of 1H and 13C NMR spectra. [file Beilstein_J_Org_Chem-08-2085-s002.pdf]

Supporting Information File 2

for

The multicomponent approach to *N*-methyl peptides: total synthesis of antibacterial (–)-viridic acid and analogues

Ricardo A. W. Neves Filho[1], Sebastian Stark[1,2], Bernhard Westermann[1,2] and Ludger A. Wessjohann[*1,2]

Address: [1]Department of Bioorganic Chemistry, Leibniz Institute of Plant Biochemistry, Weinberg 3, 06120 Halle (Saale), Germany, Tel: +49 345 5582 1301, Fax: +49 345 5582 1309 (Address for correspondence) and [2]Martin-Luther-University Halle-Wittenberg, Institute of Organic Chemistry, Kurt-Mothes-Str. 2, 06120 Halle (Saale), Germany

Email: Ludger A. Wessjohann - wessjohann@ipb-halle.de.

* Corresponding author:

Figures of ^{1}H and ^{13}C NMR spectra

Please note that spectra of N-alkyl-amides (peptoids) like Ugi products display double signal sets due to interconvertible isomers with *s-cis* and *s-trans*-amide bonds. Depending on the substitution pattern, solvent and temperature, the equilibrium between these forms is shifted and may lead to broadened or doubled peaks of varied intensity.

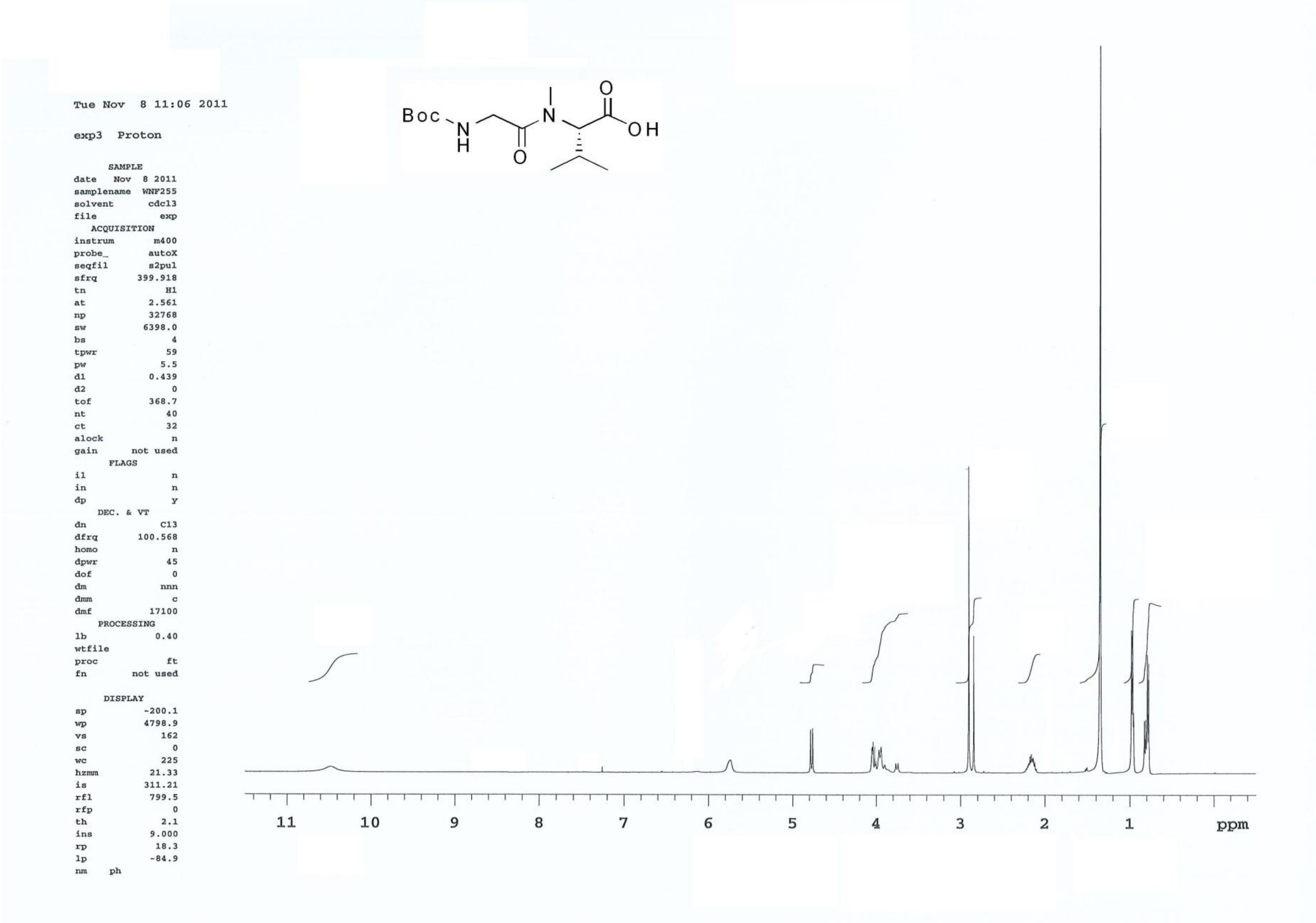

Figure S1: ^{1}H NMR spectrum of compound **6**.

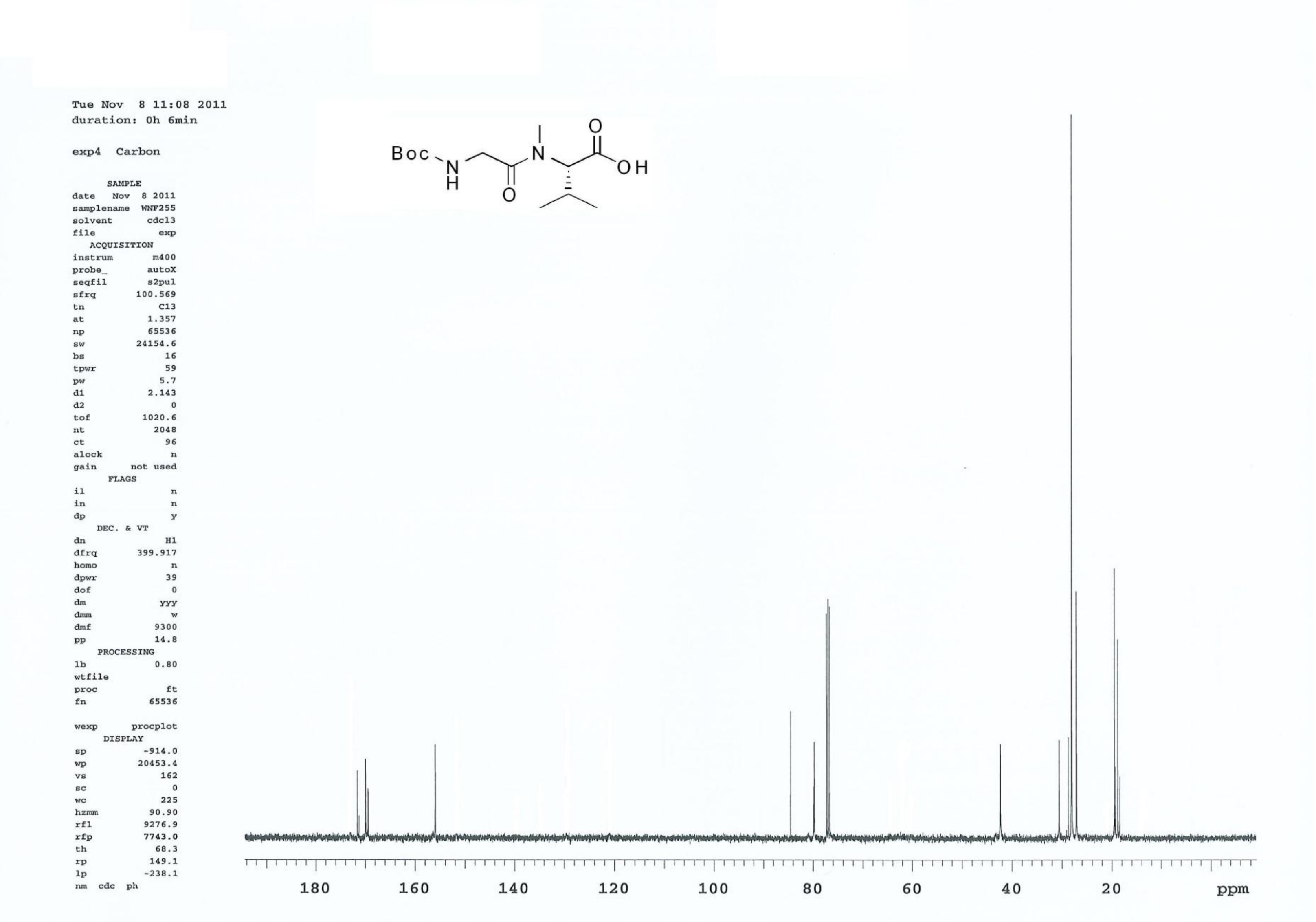

Figure S2: ^{13}C NMR spectrum of compound **6**.

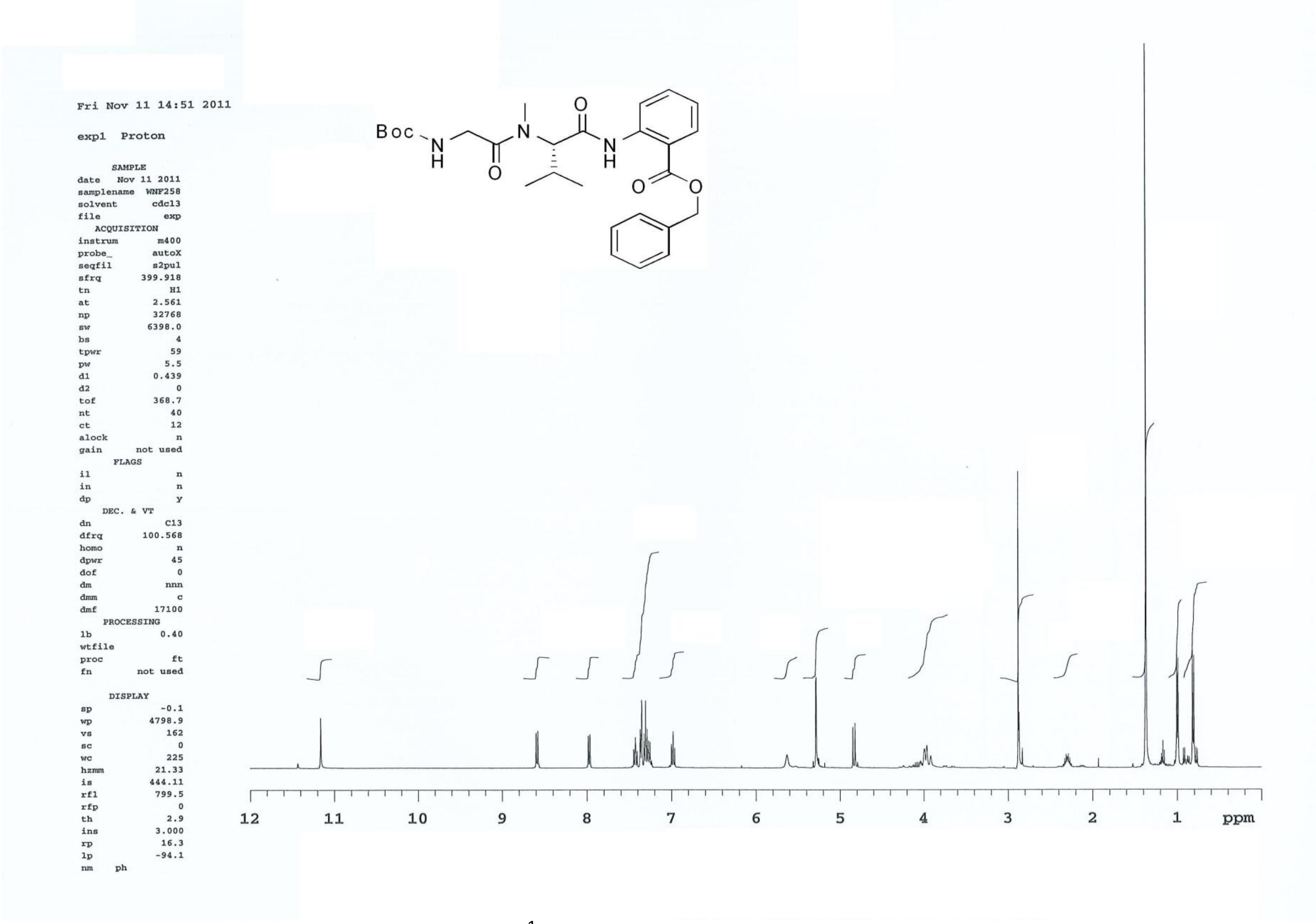

Figure S3: ^{1}H NMR spectrum of compound **7**.

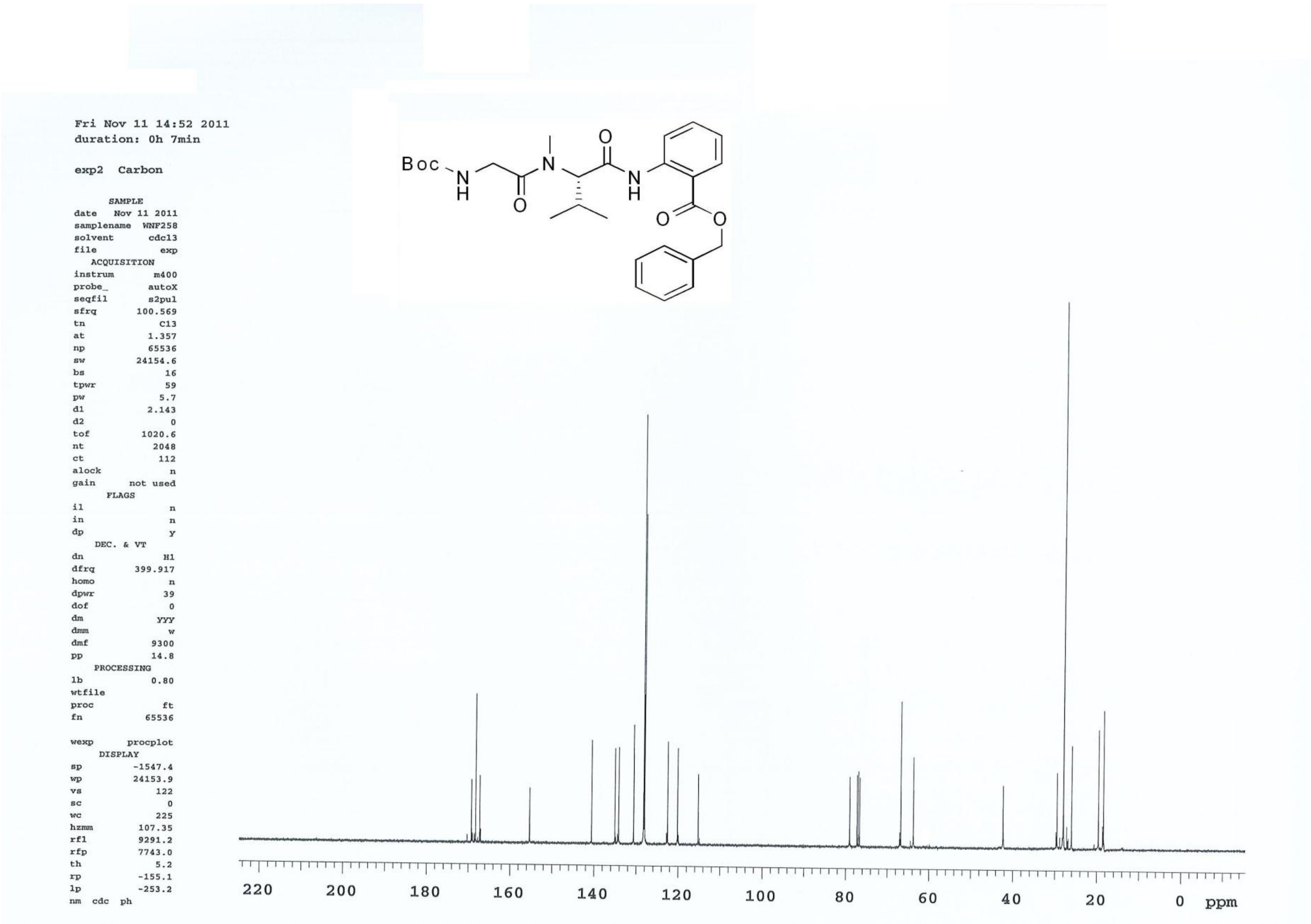

Figure S4: ^{13}C NMR spectrum of compound **7**.

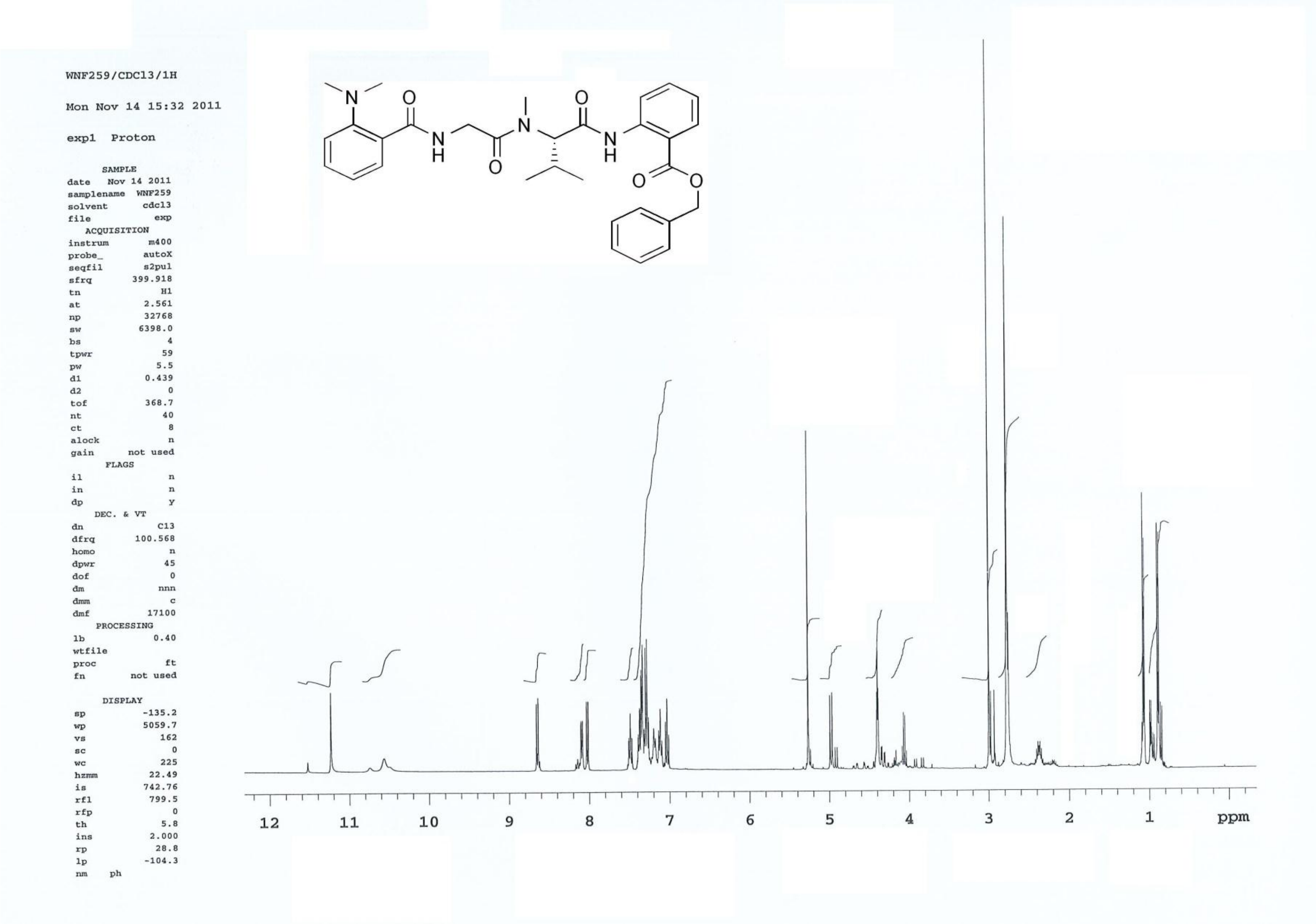

Figure S5: ^{1}H NMR spectrum of compound **2**.

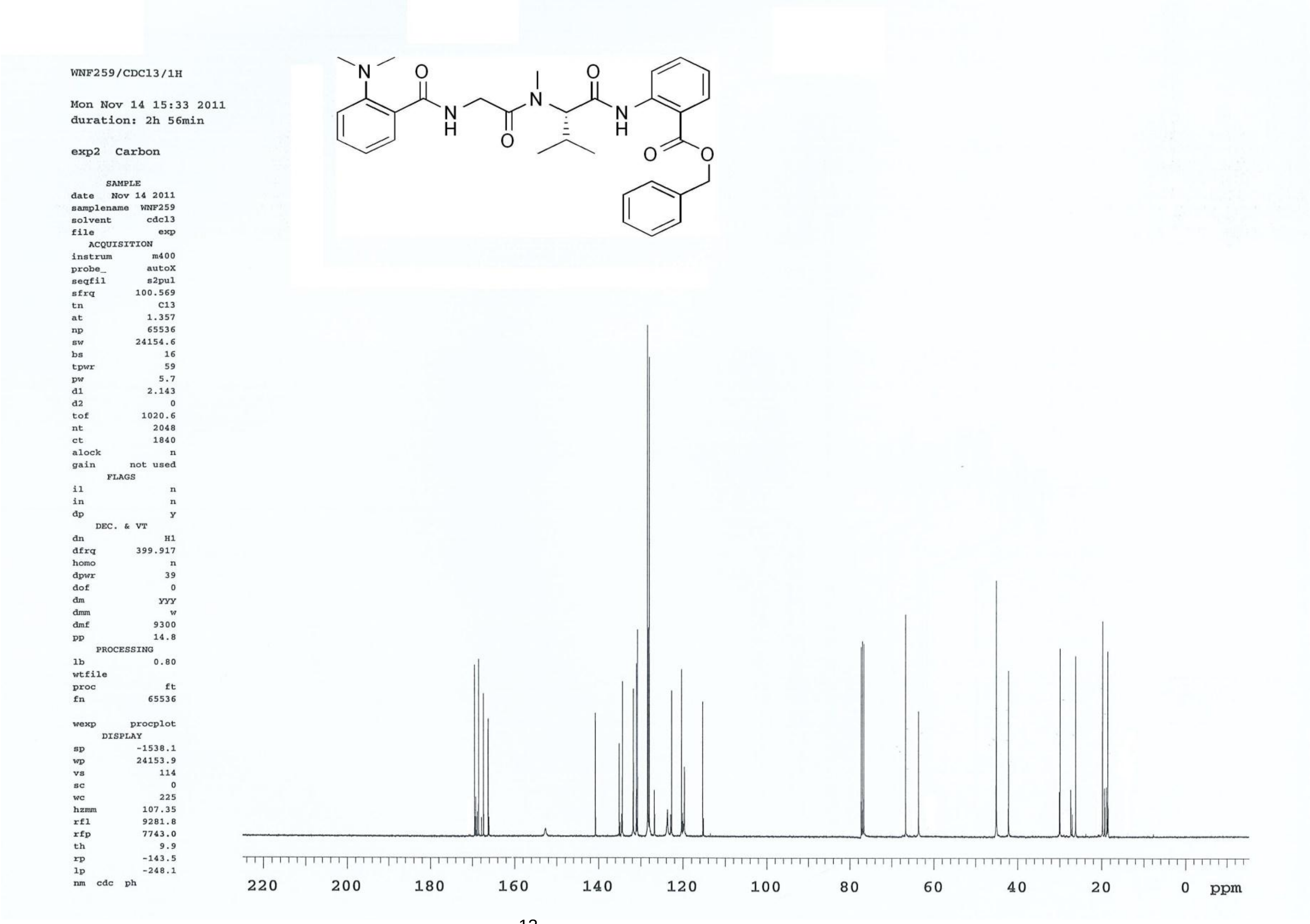

Figure S6: ^{13}C NMR spectrum of compound **2**.

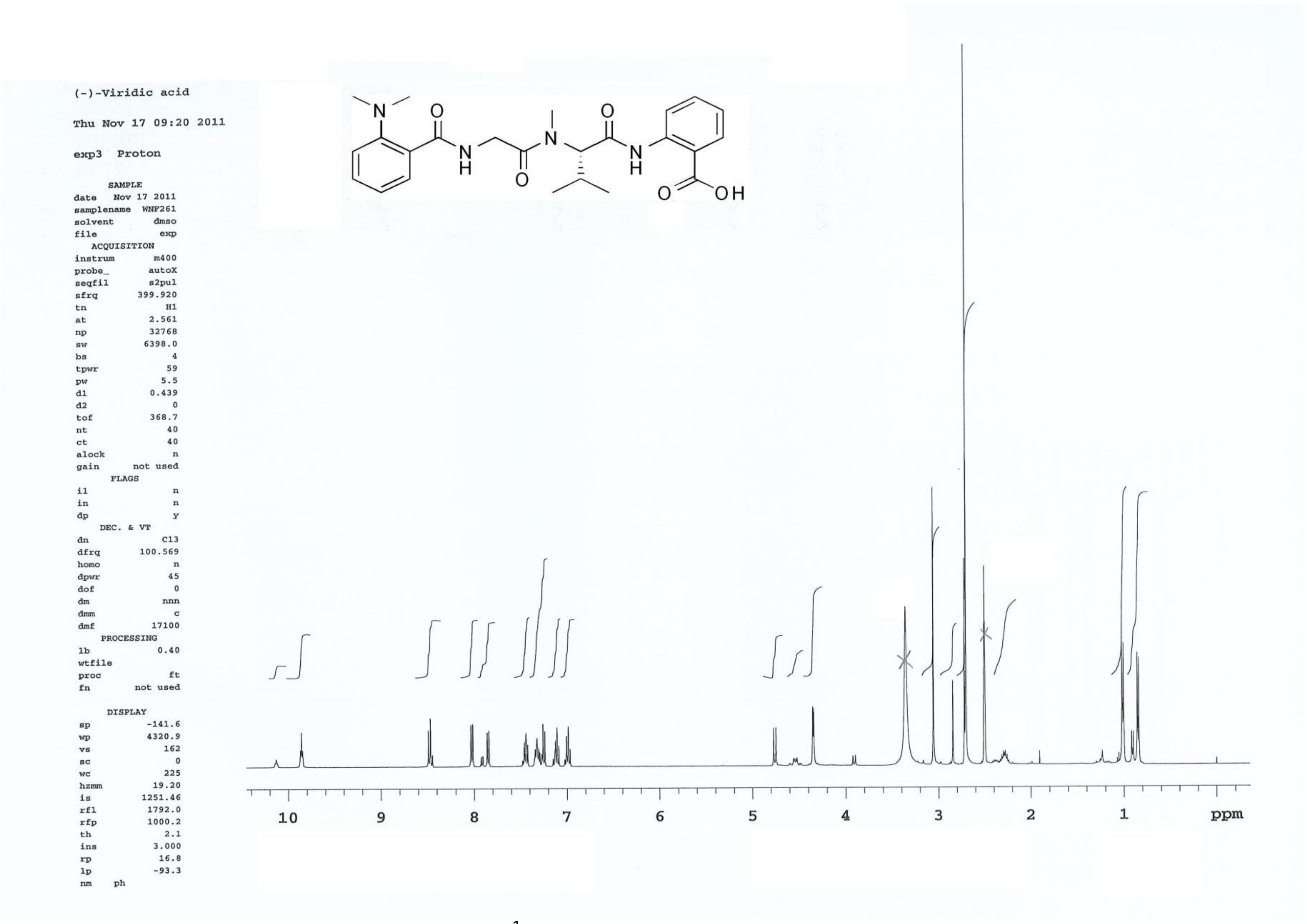

Figure S7: ^{1}H NMR spectrum of viridic acid (**1**).

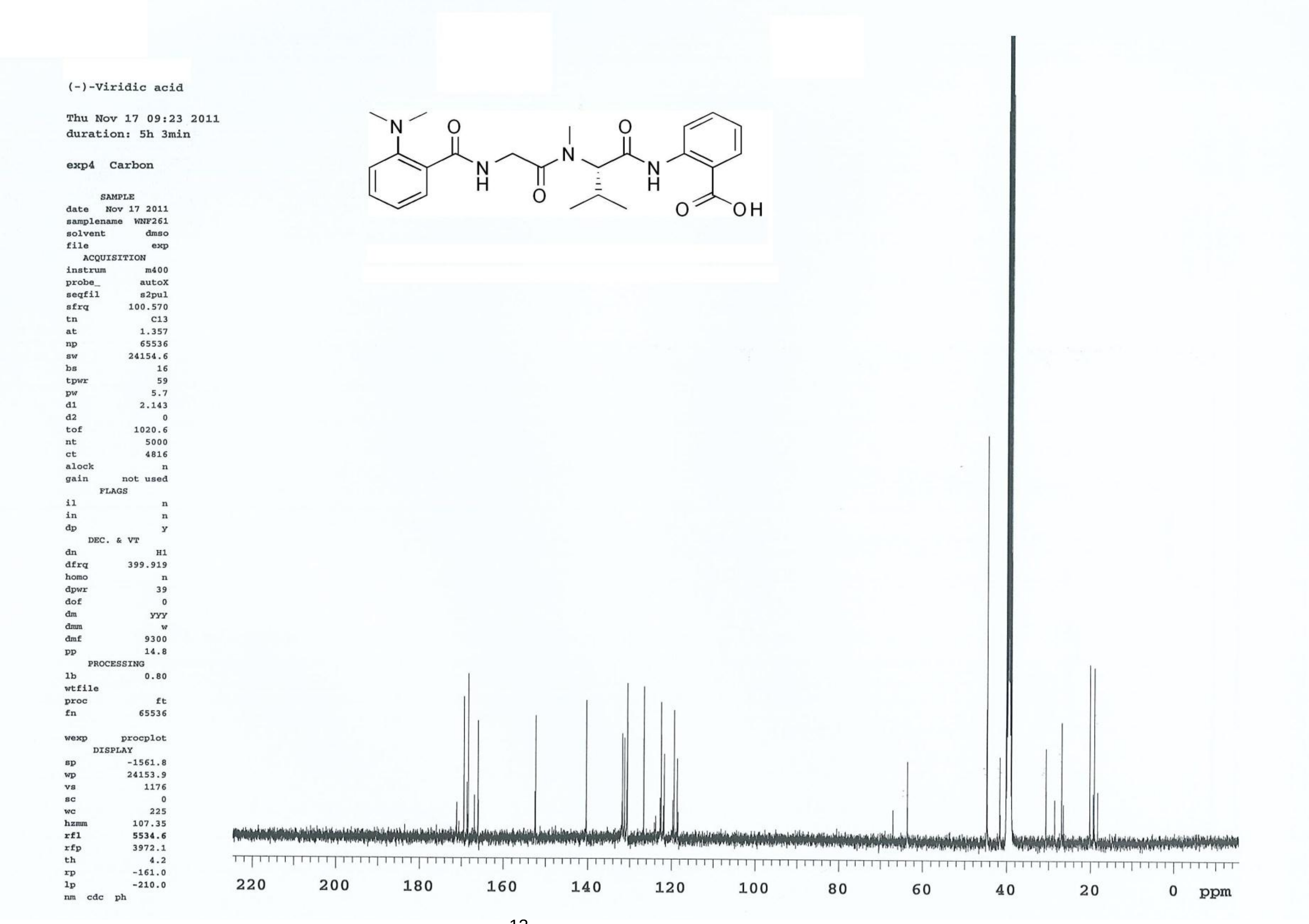

Figure S8: ^{13}C NMR spectrum of viridic acid (**1**).

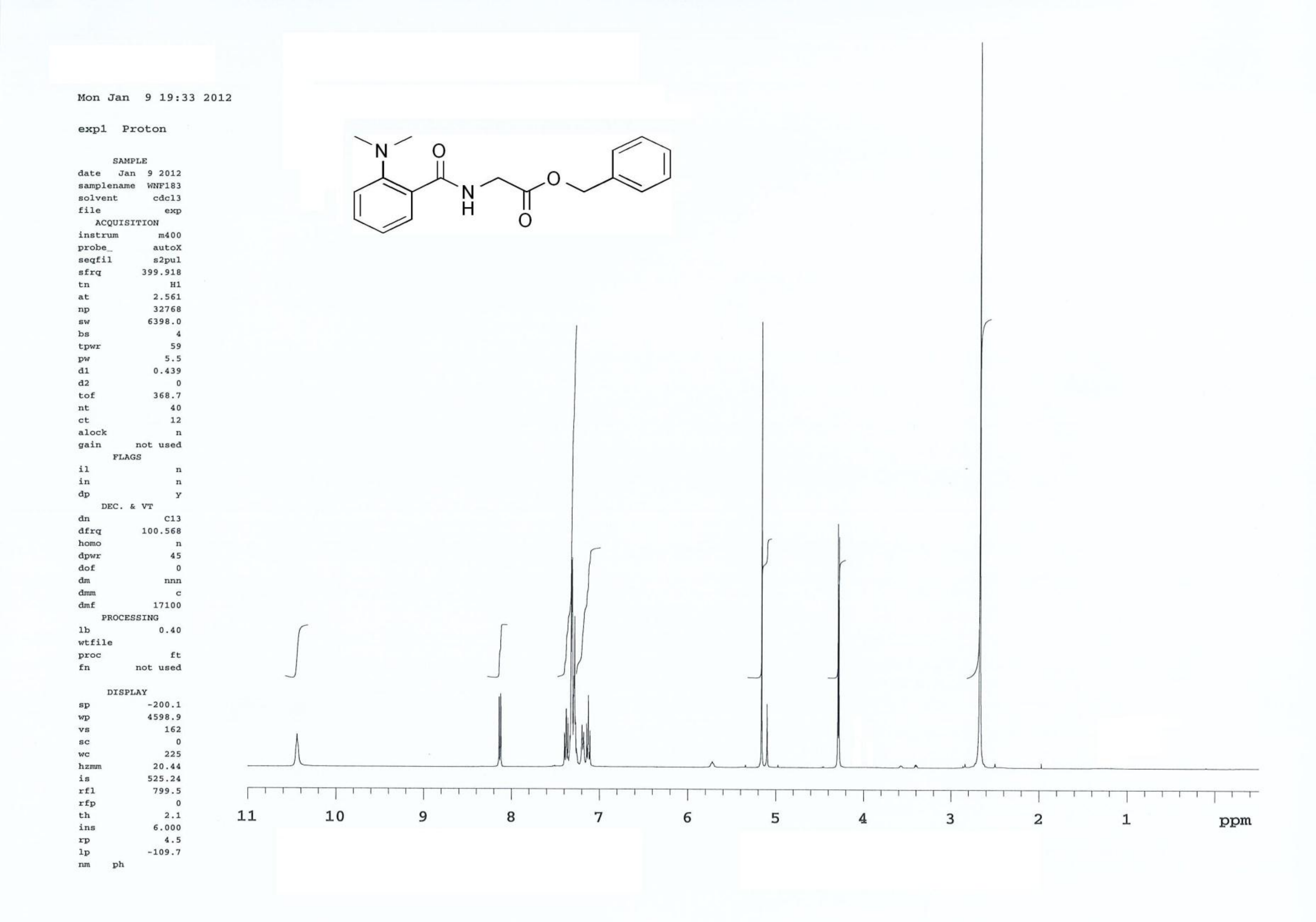

Figure S9: ^{1}H NMR spectrum of compound **10**.

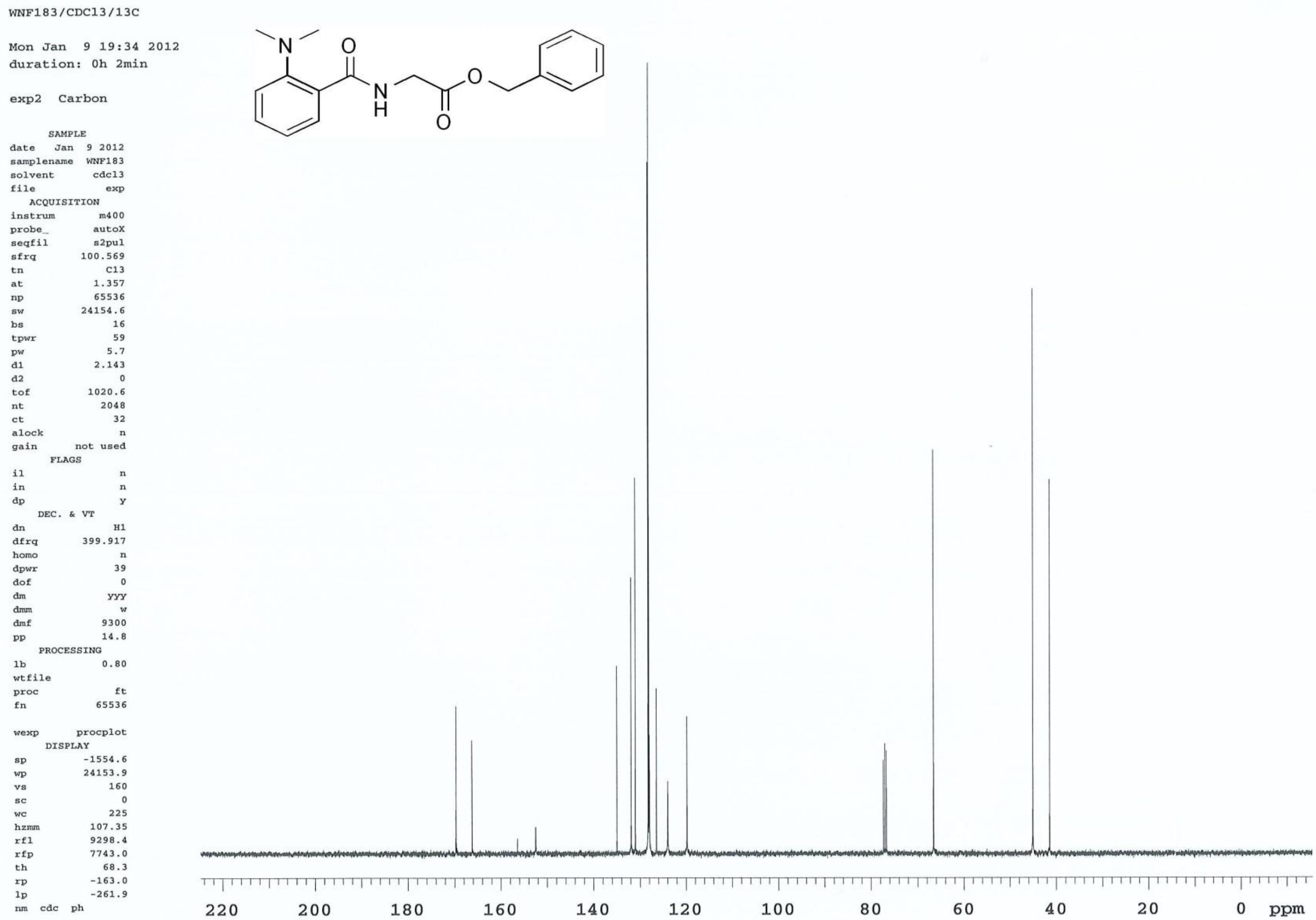

Figure S10: ^{13}C NMR spectrum of compound **10**.

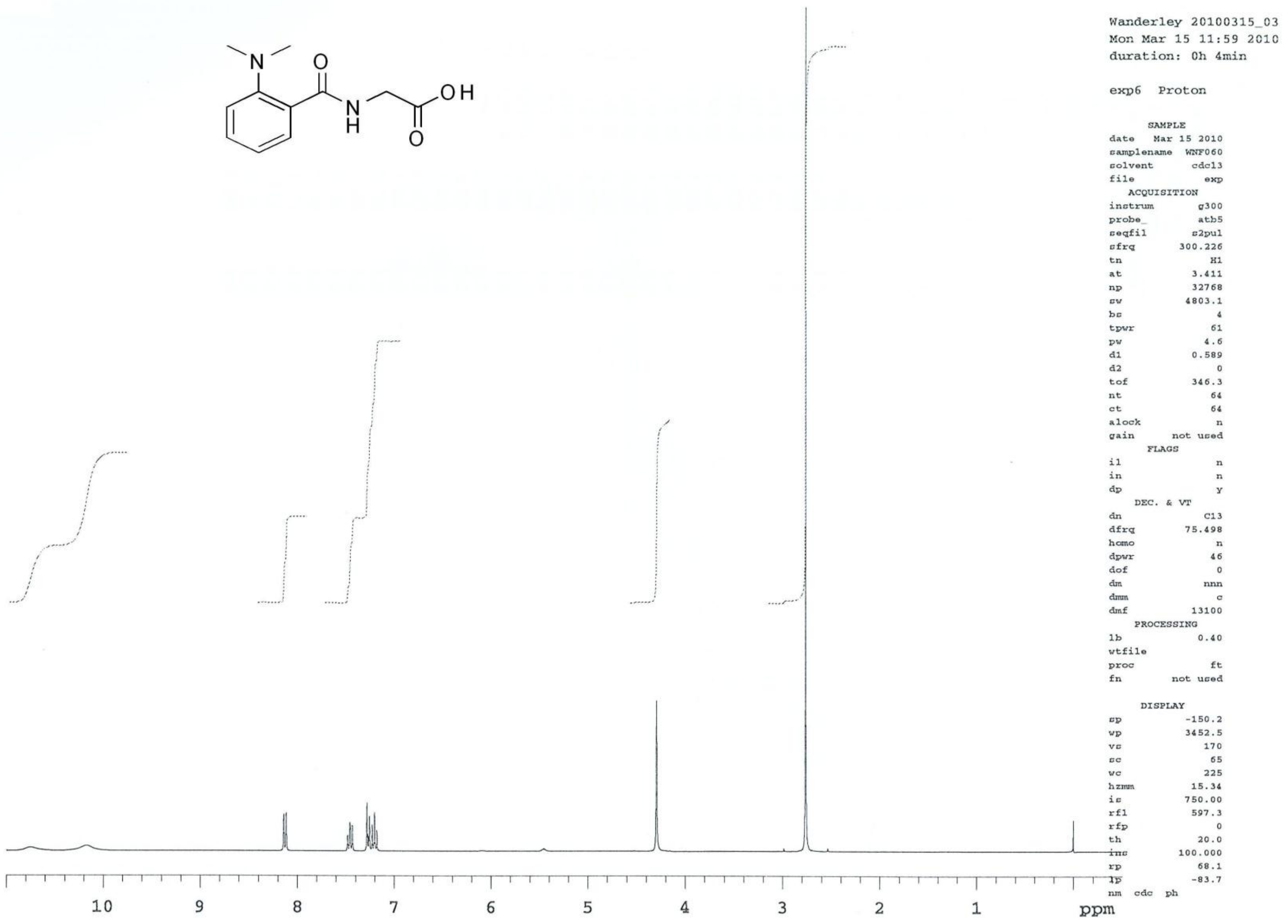

Figure S11: ^{1}H NMR spectrum of compound **3**.

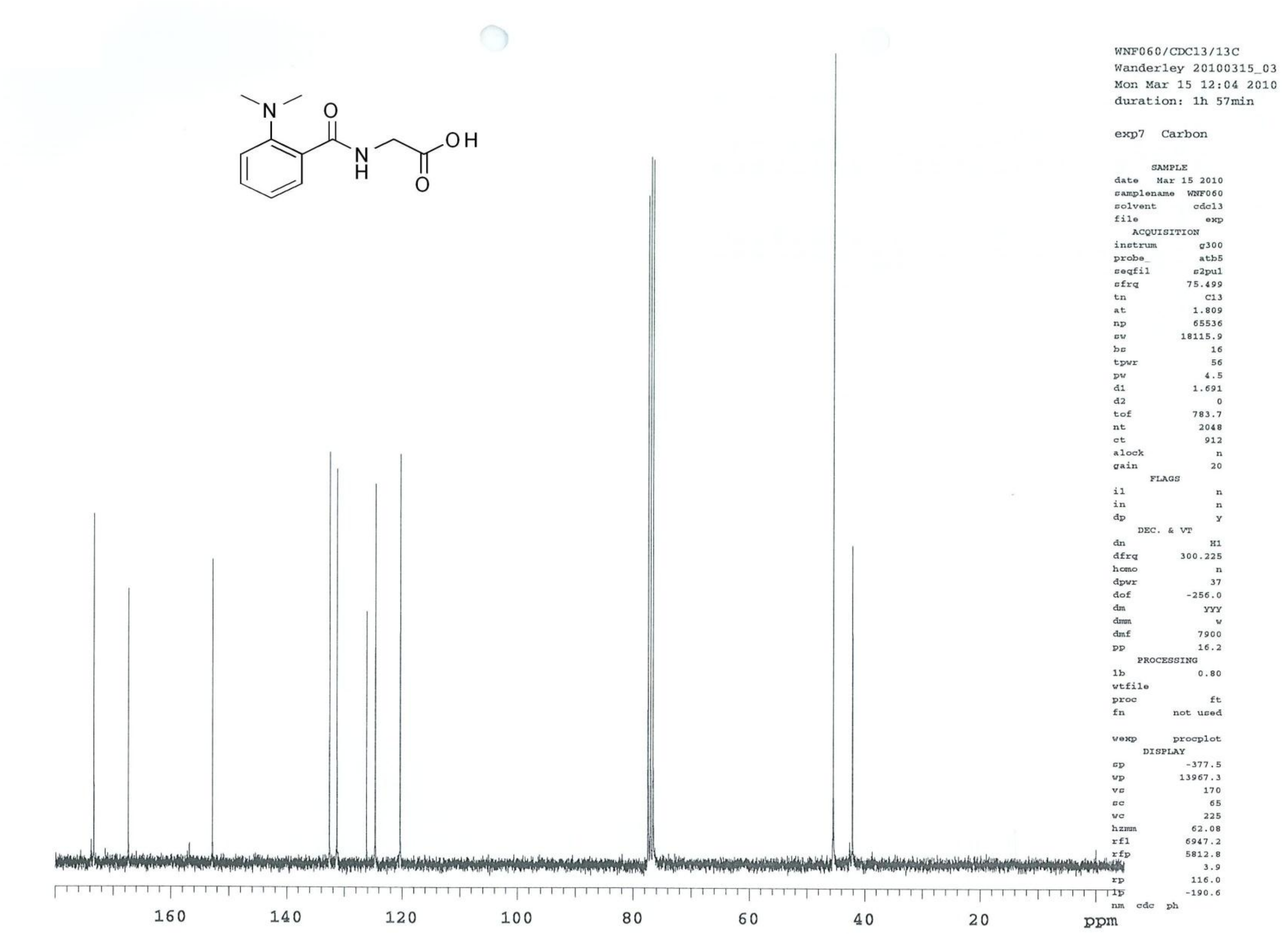

Figure S12: ^{13}C NMR spectrum of compound **3**.

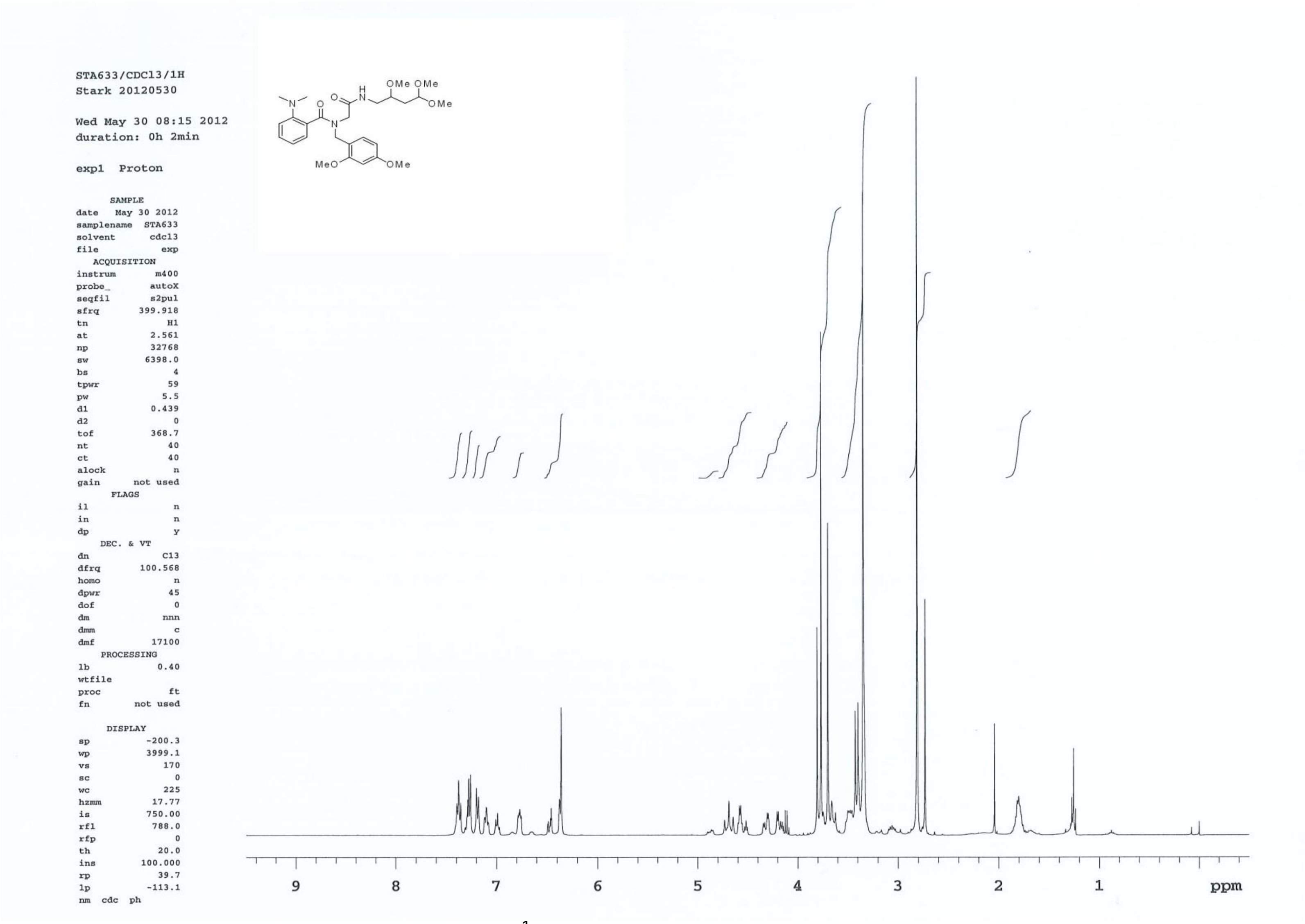

Figure S13: ^{1}H NMR spectrum of compound **11**.

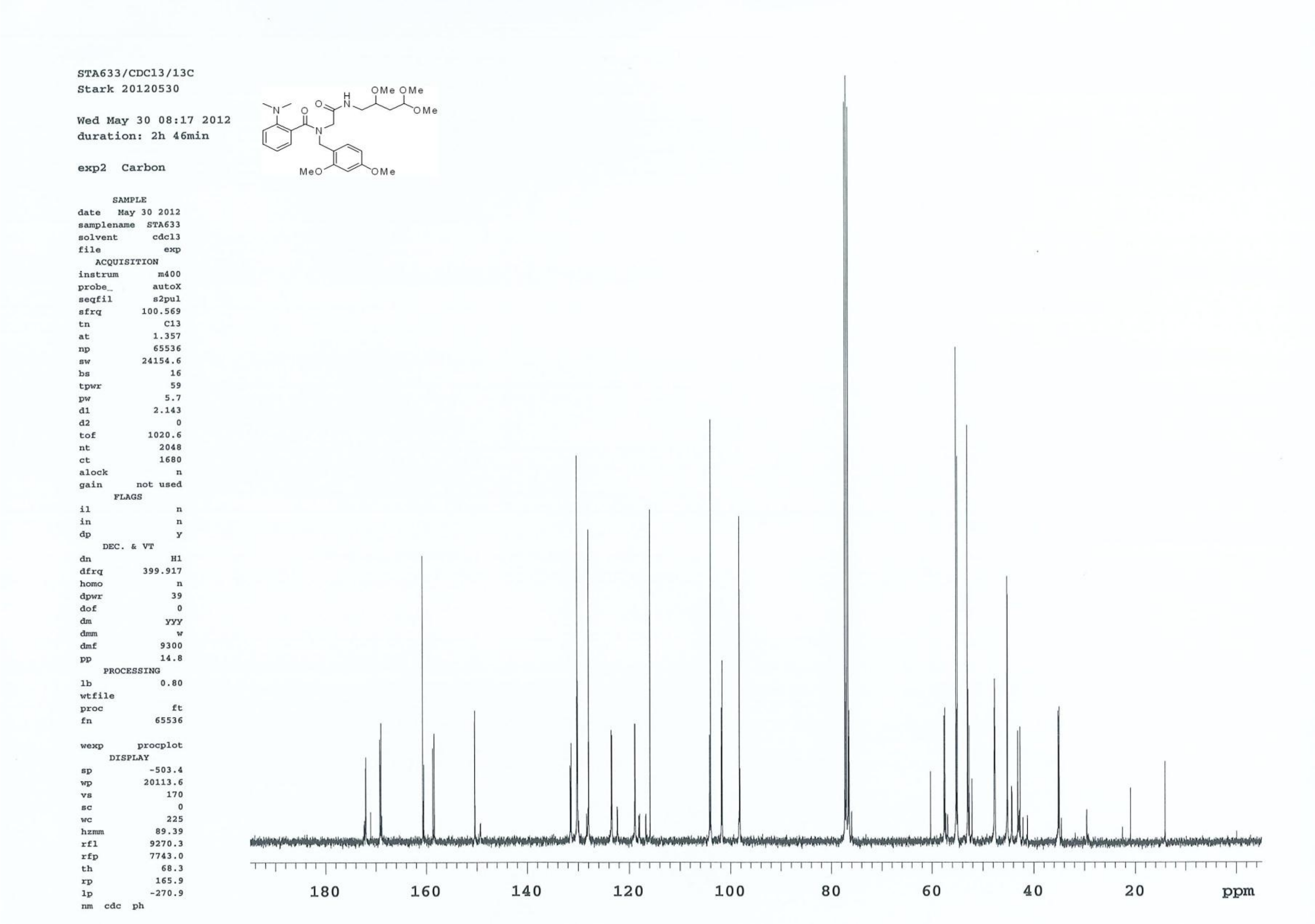

Figure S14: ^{13}C NMR spectrum of compound **11**.

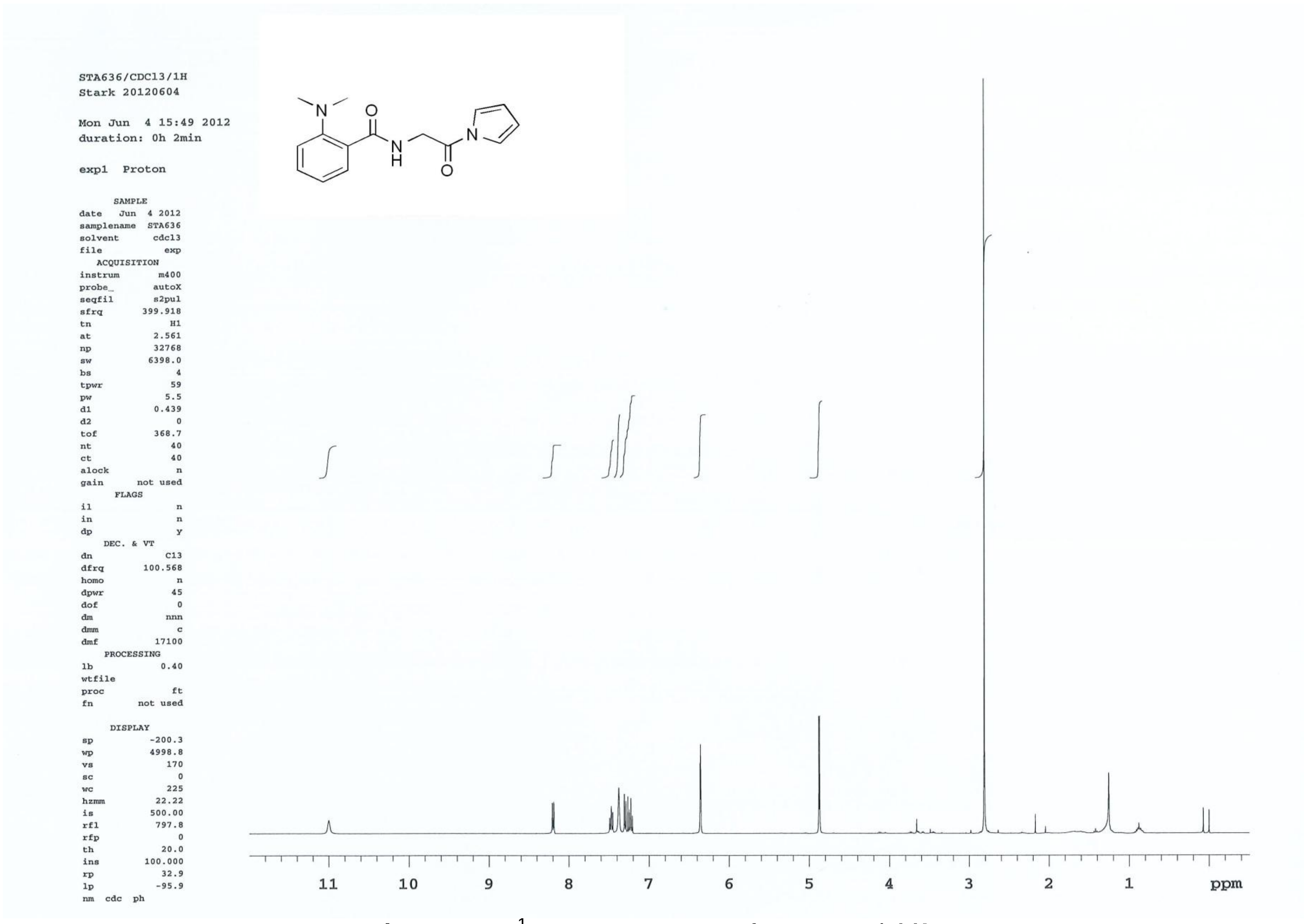

Figure S15: ^{1}H NMR spectrum of compound **11’**.

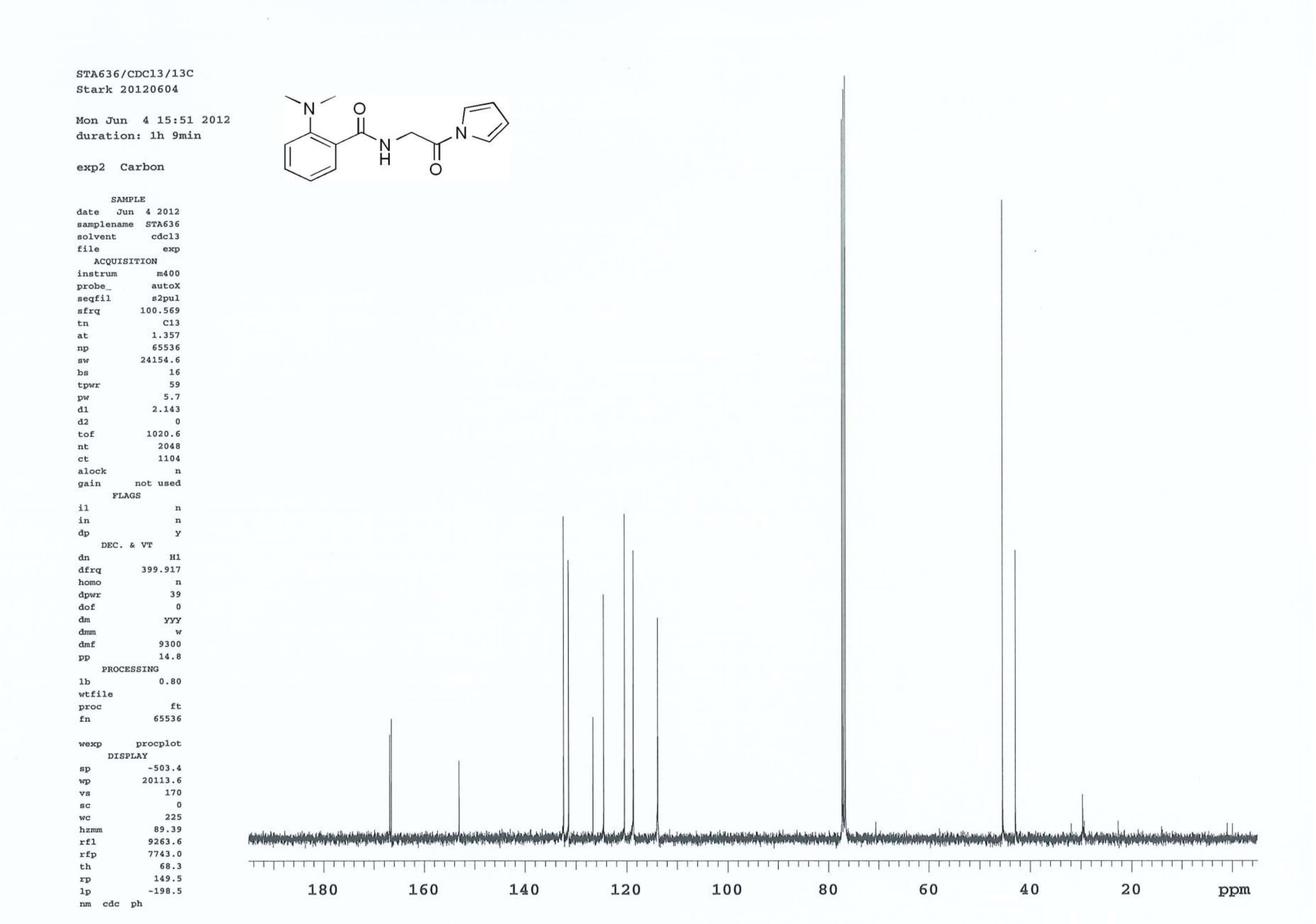

Figure S16: ^{13}C NMR spectrum of compound **11'**.

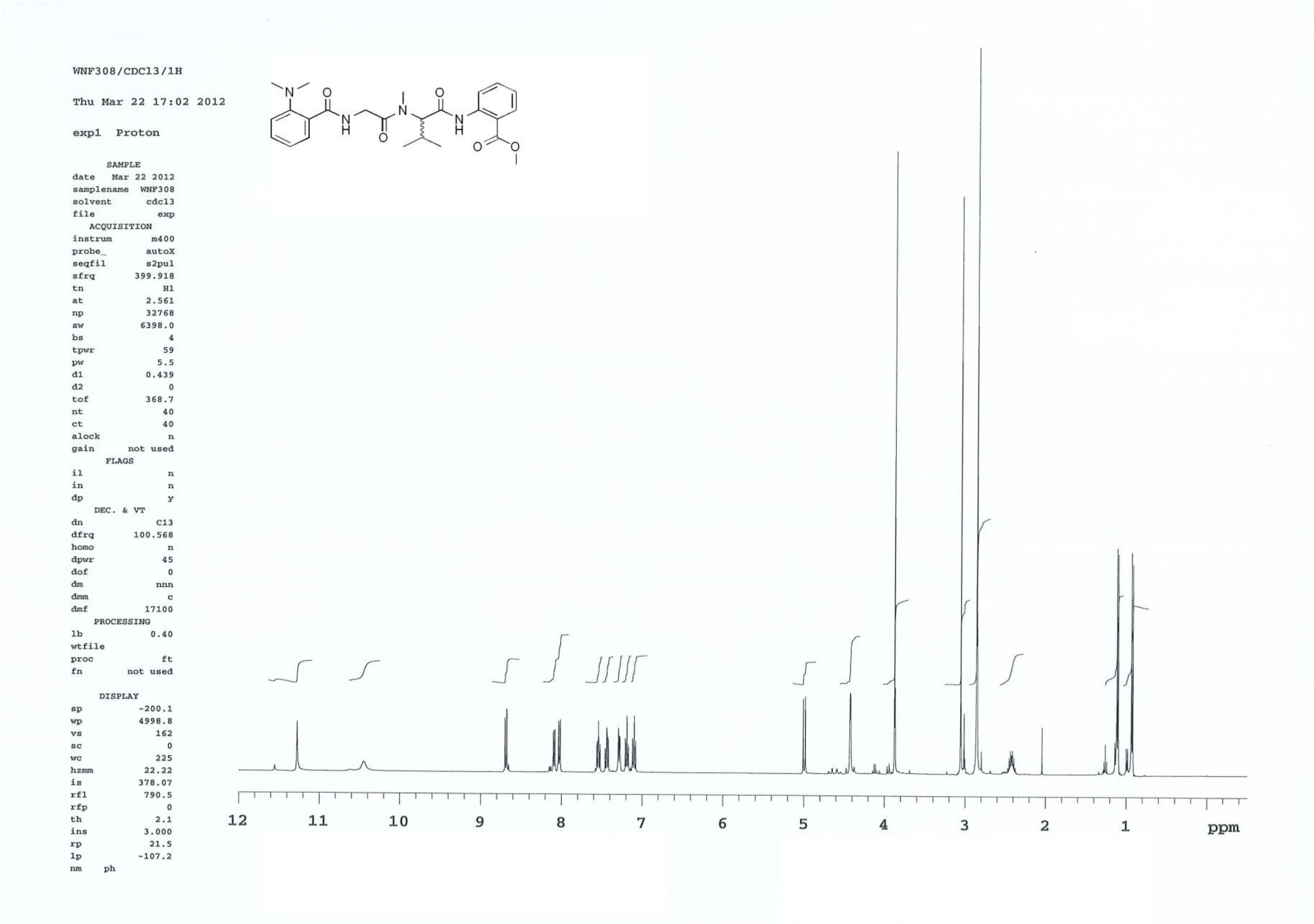

Figure S17: ^{1}H NMR spectrum of compound **13a**.

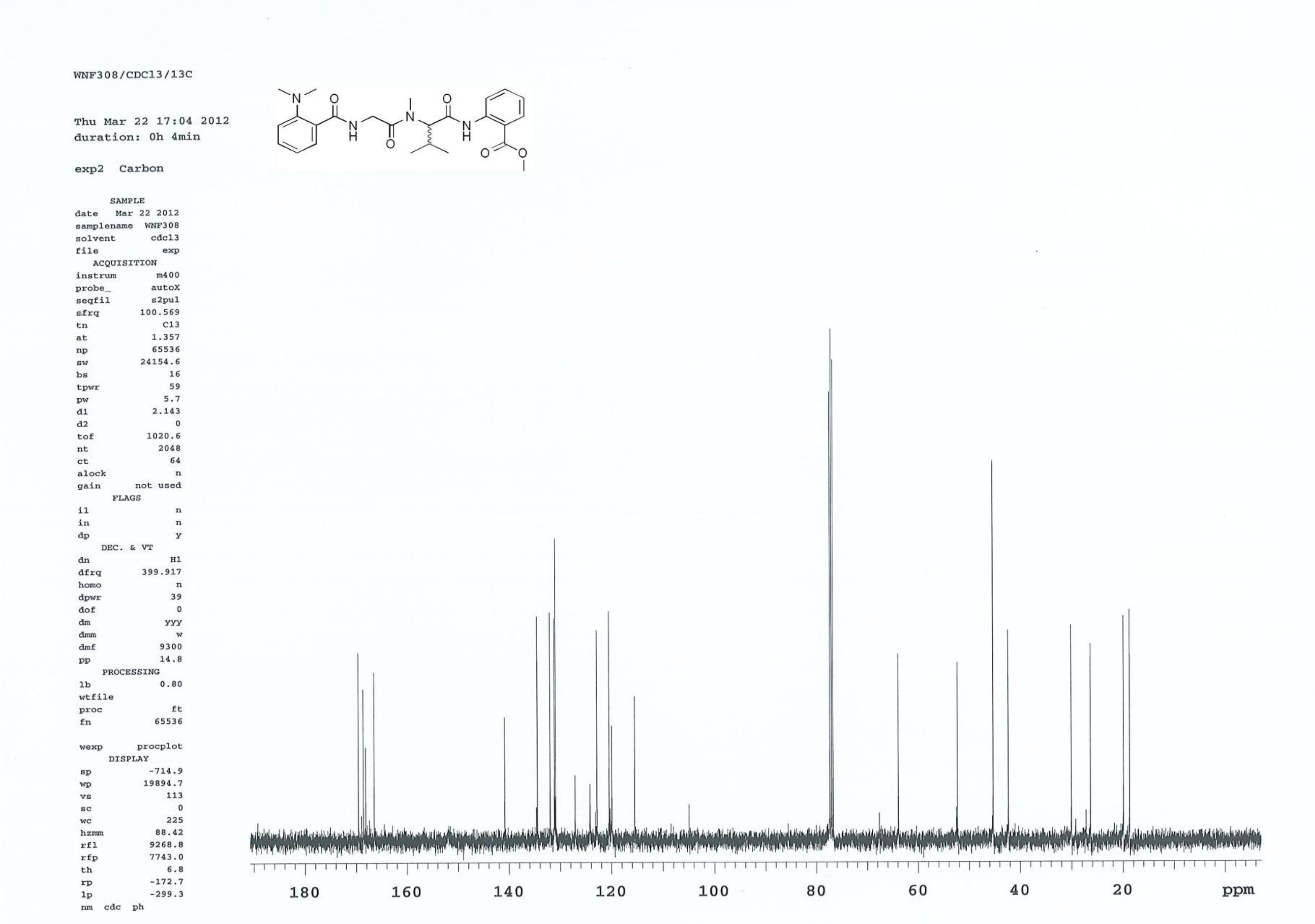

Figure S18: ^{13}C NMR spectrum of compound **13a**.

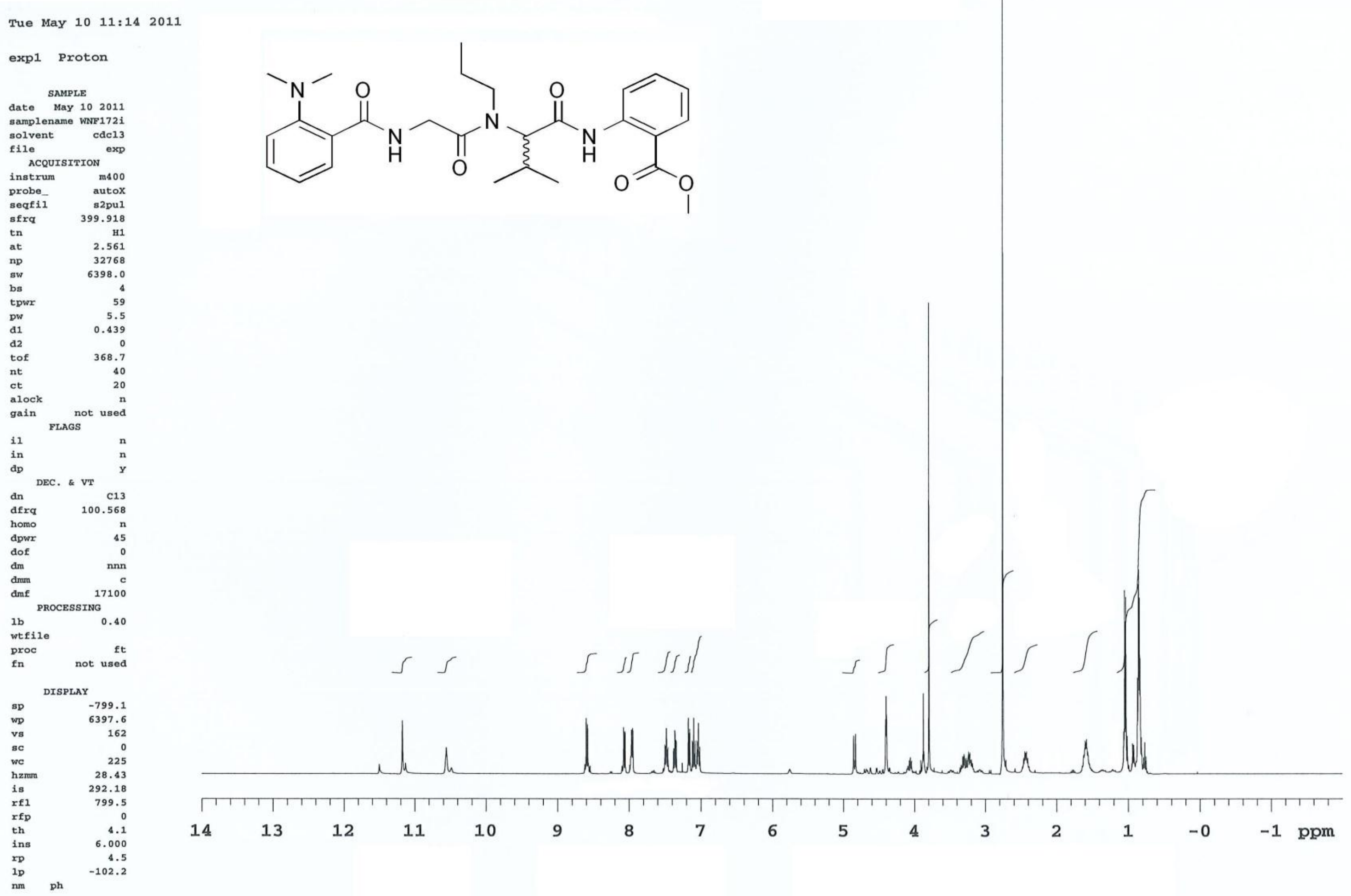

Figure S19: ^{1}H NMR spectrum of compound **13b**.

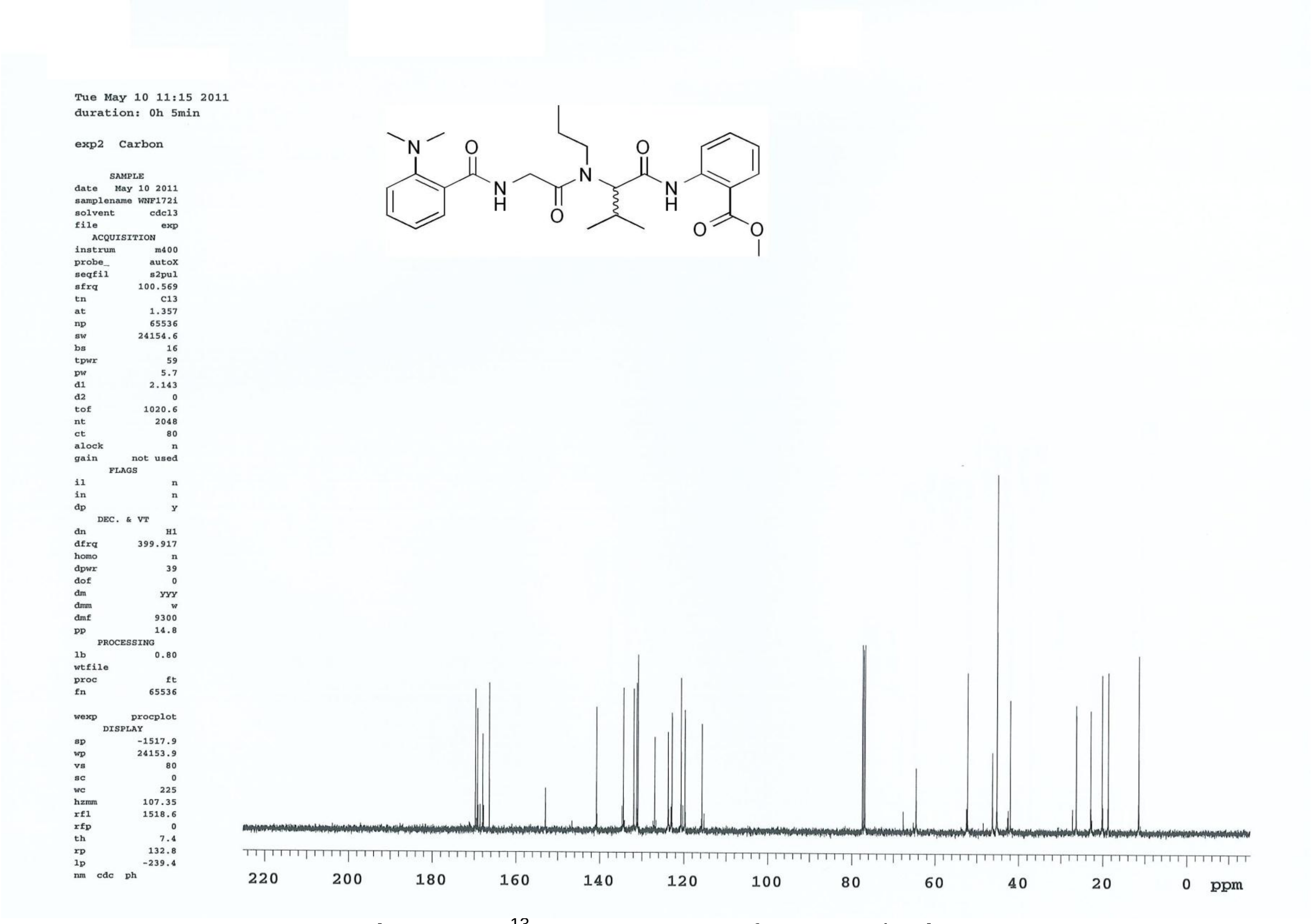

Figure S20: ^{13}C NMR spectrum of compound **13b**.

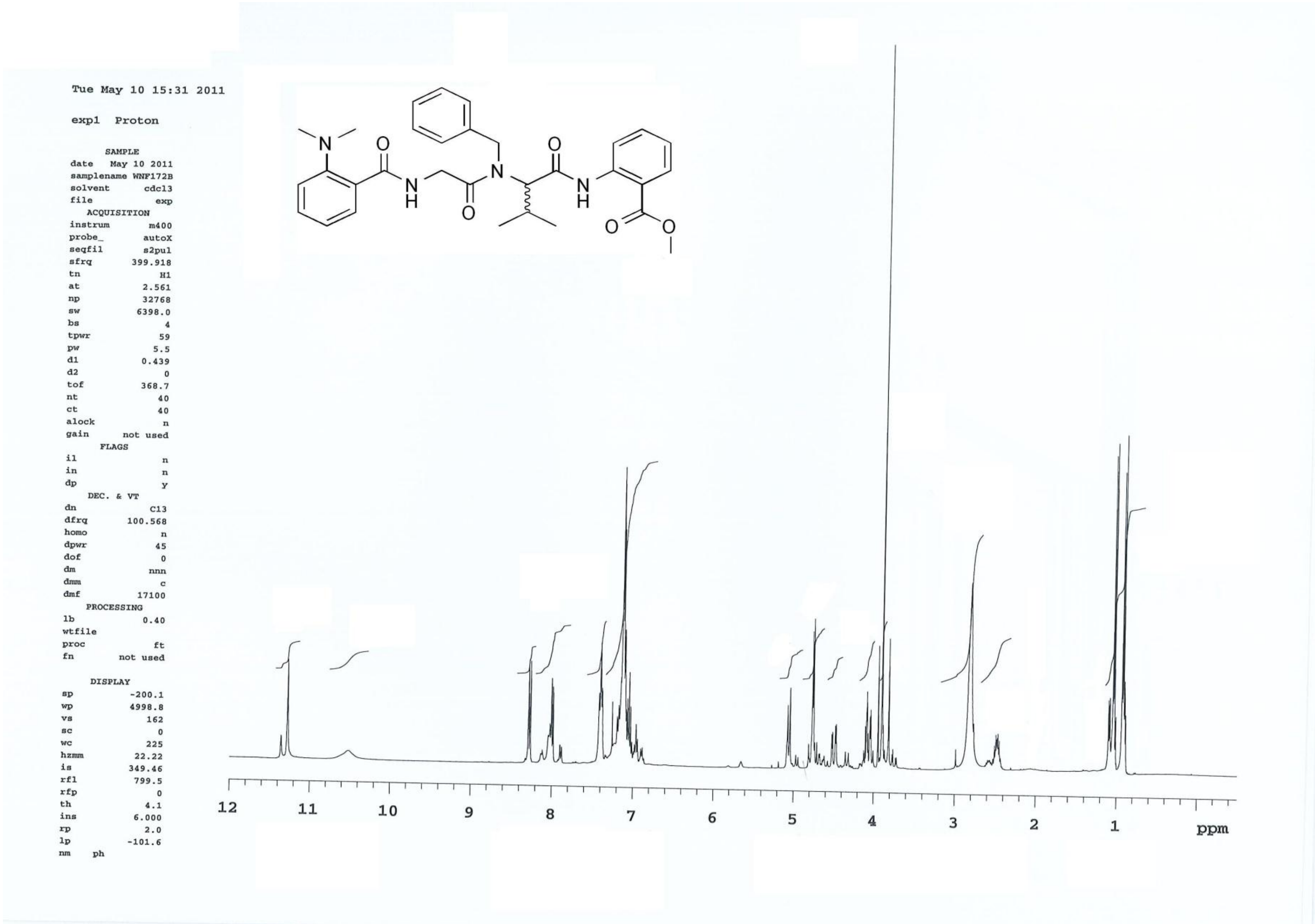

Figure S21: ^{1}H NMR spectrum of compound **13c**.

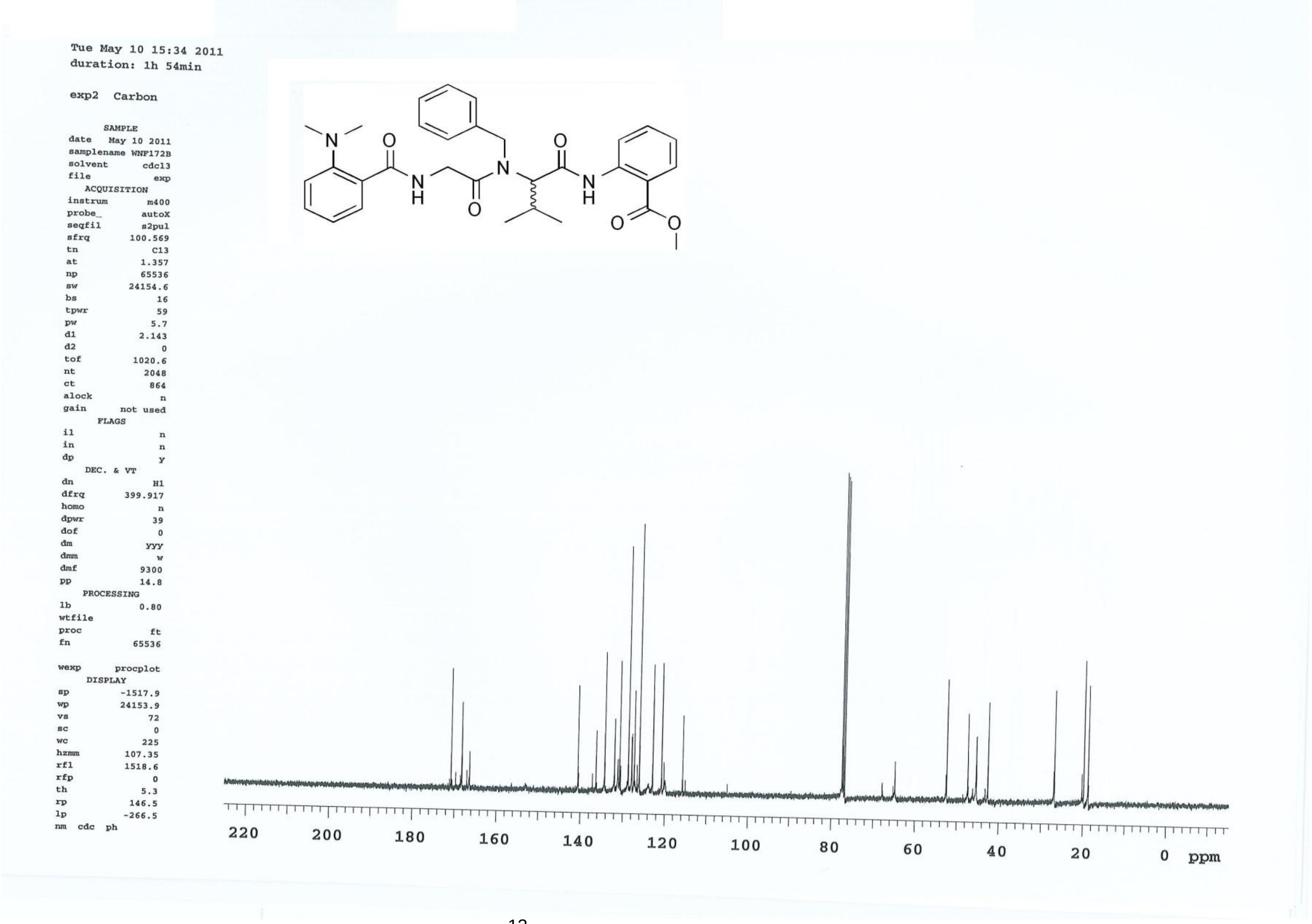

Figure S22: ^{13}C NMR spectrum of compound **13c**.

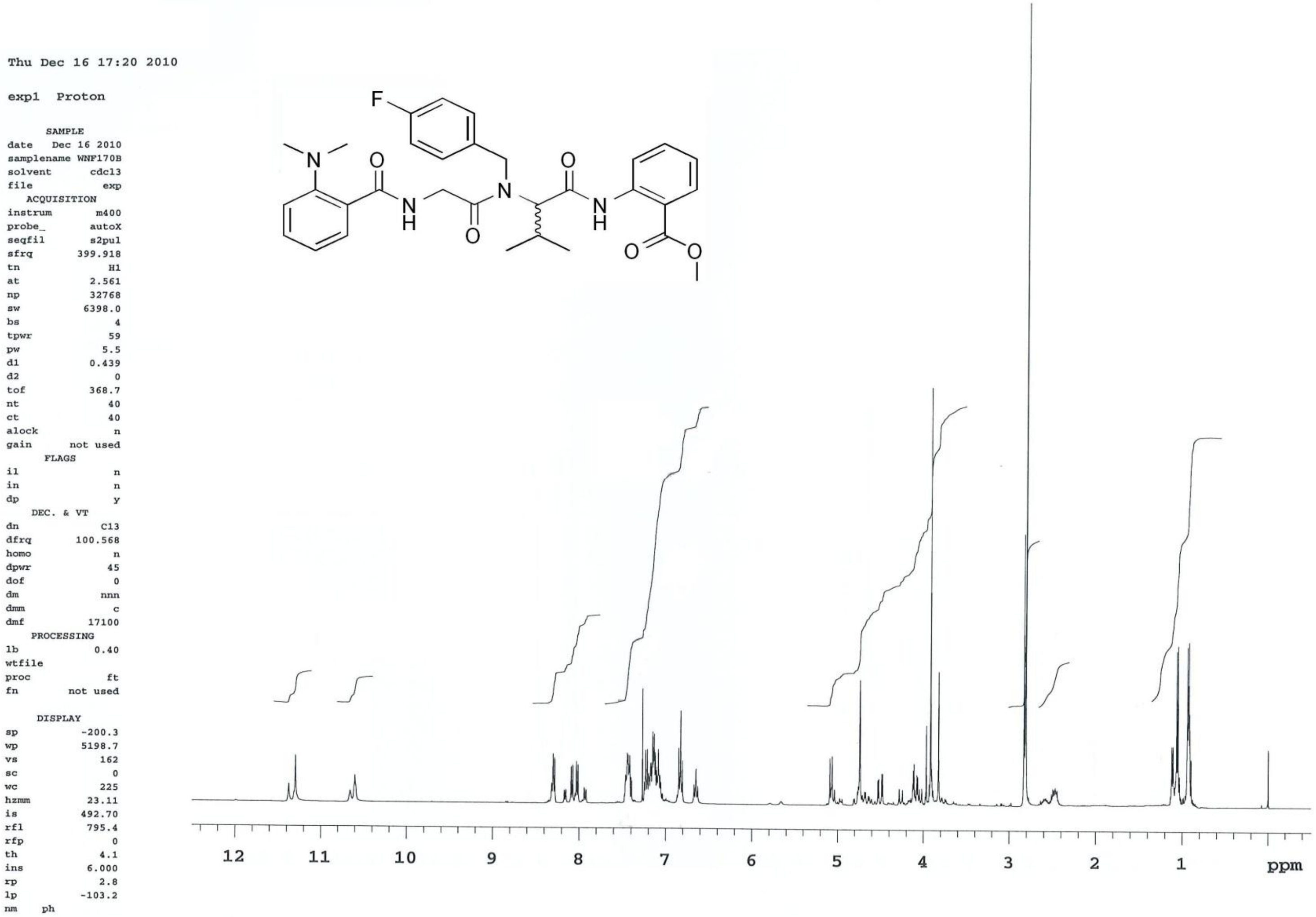

Figure S23: ^{1}H NMR spectrum of compound **13d**.

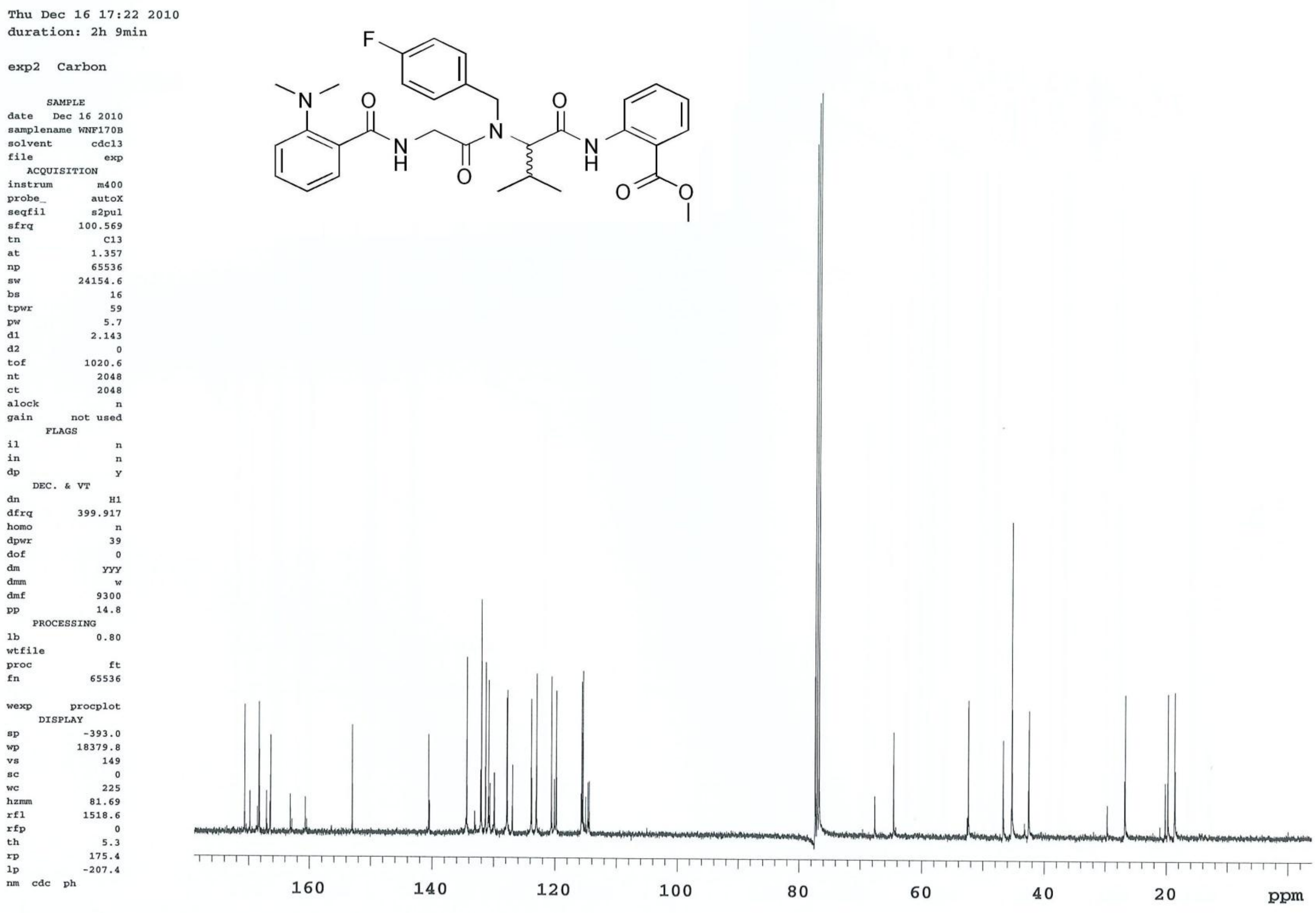

Figure S24: ^{13}C NMR spectrum of compound **13d**.

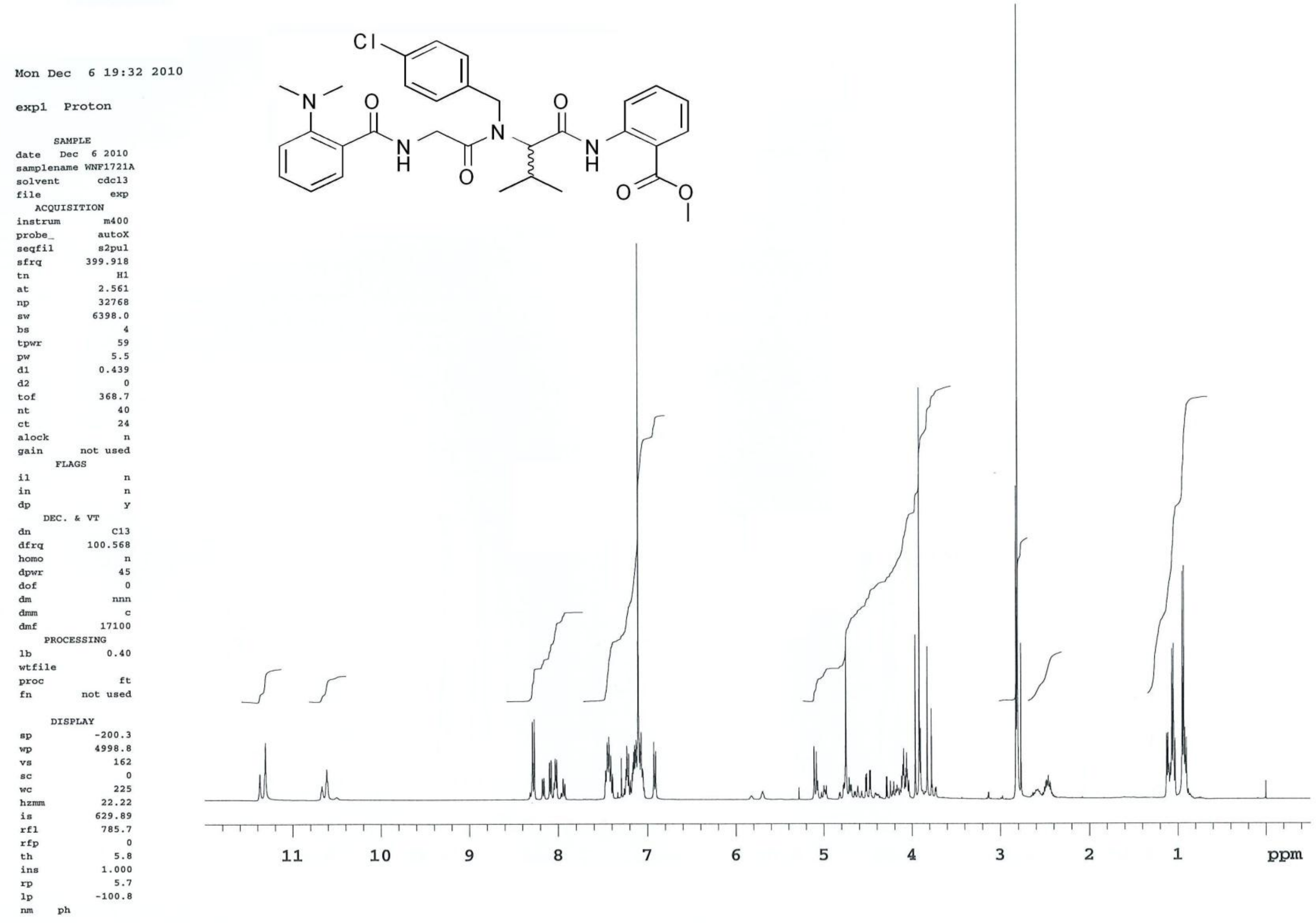

Figure S25: ^{1}H NMR spectrum of compound **13e**.

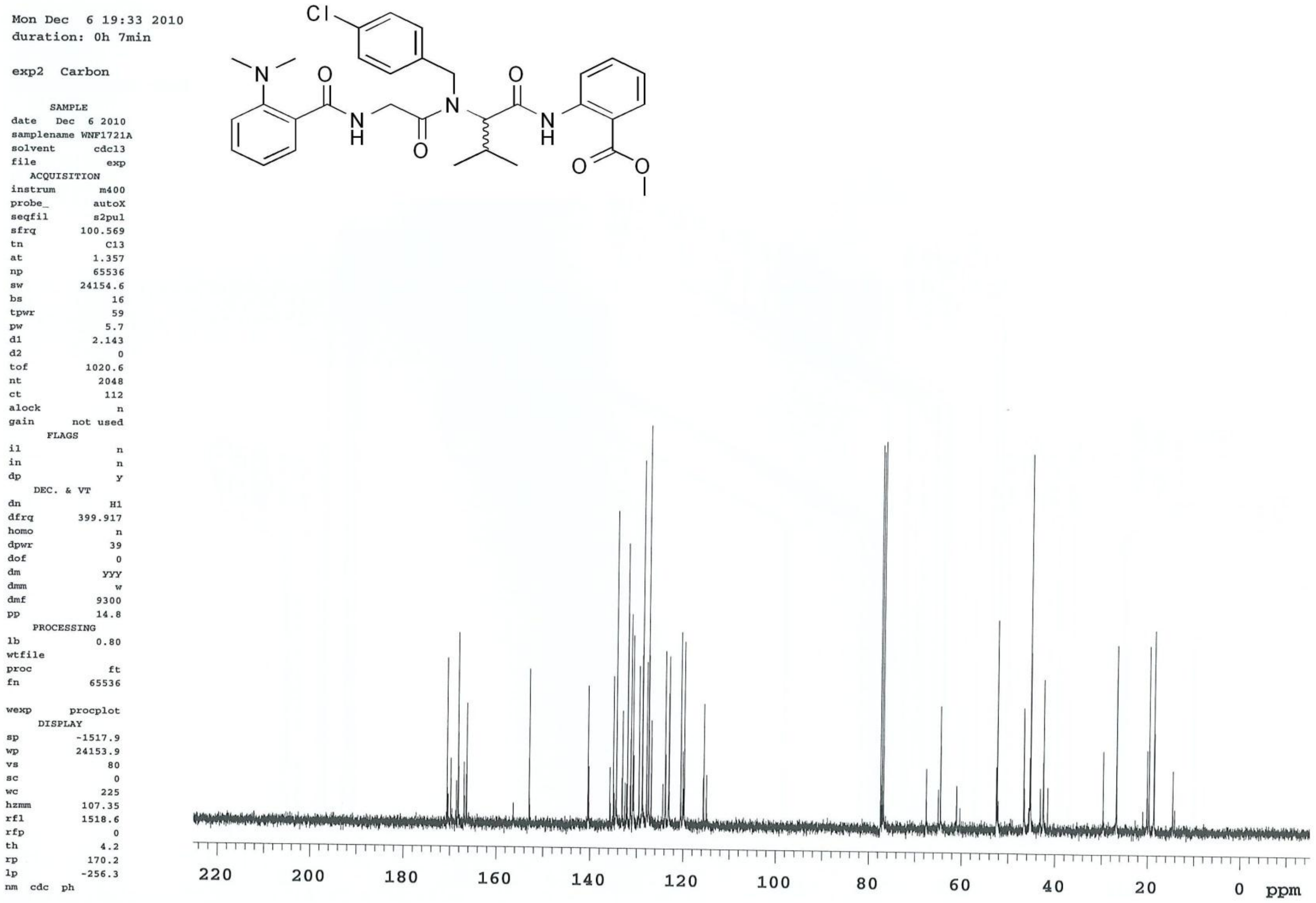

Figure S26: ^{13}C NMR spectrum of compound **13e**.

Tue May 10 16:29 2011

exp1 Proton

SAMPLE
date May 10 2011
samplename WNF172G
solvent cdcl3
file exp
ACQUISITION
instrum m400
probe_ autoX
seqfil s2pul
sfrq 399.918
tn H1
at 2.561
np 32768
sw 6398.0
bs 4
tpwr 59
pw 5.5
d1 0.439
d2 0
tof 368.7
nt 40
ct 20
alock n
gain not used
FLAGS
il n
in n
dp y
DEC. & VT
dn C13
dfrq 100.568
homo n
dpwr 45
dof 0
dm nnn
dmm c
dmf 17100
PROCESSING
lb 0.40
wtfile
proc ft
fn not used

DISPLAY
sp -0.1
wp 4998.8
vs 162
sc 0
wc 225
hzmm 22.22
is 489.02
rfl 799.5
rfp 0
th 4.1
ins 6.000
rp 2.6
lp -86.0
nm ph

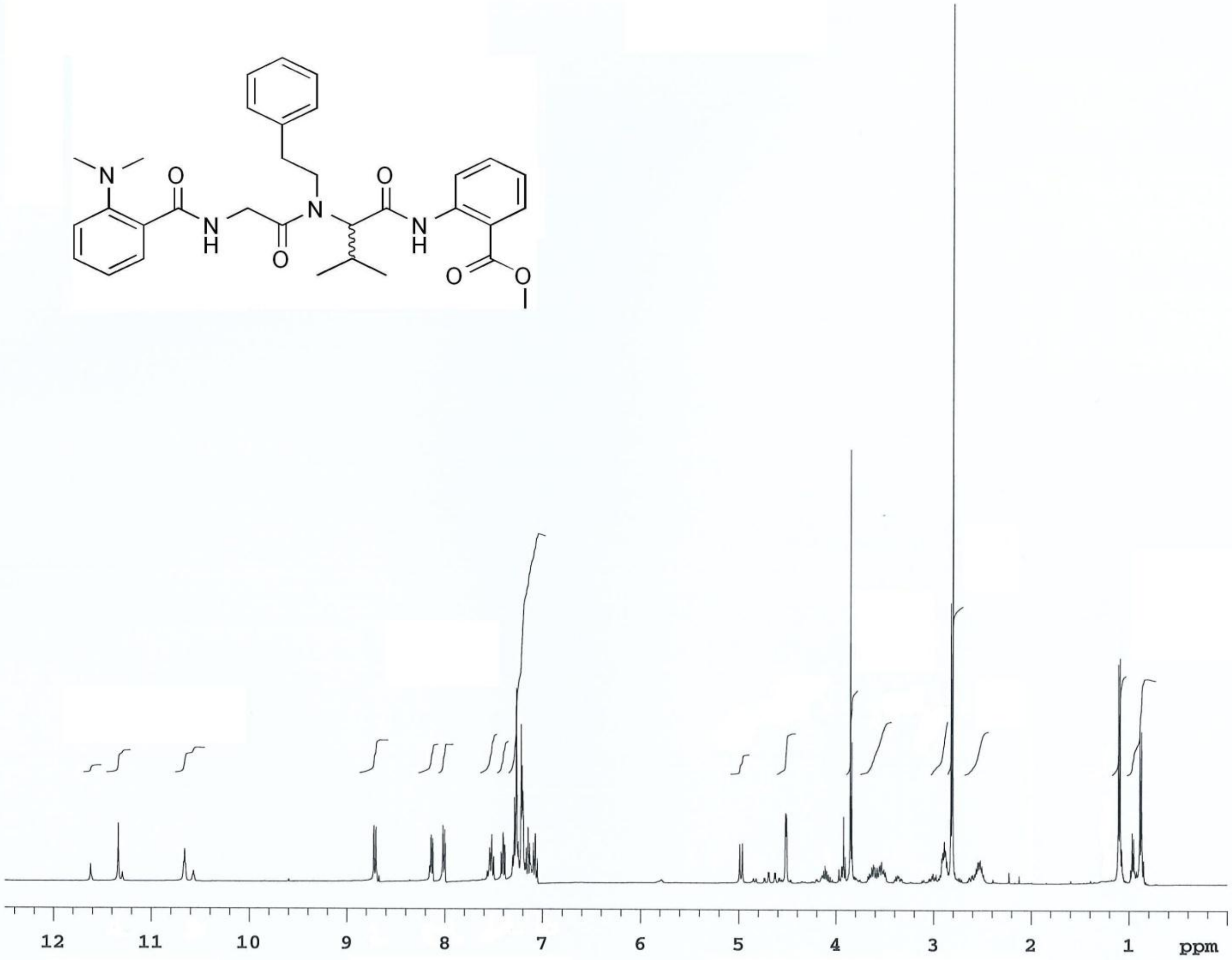

Figure S27: ^{1}H NMR spectrum of compound **13f**.

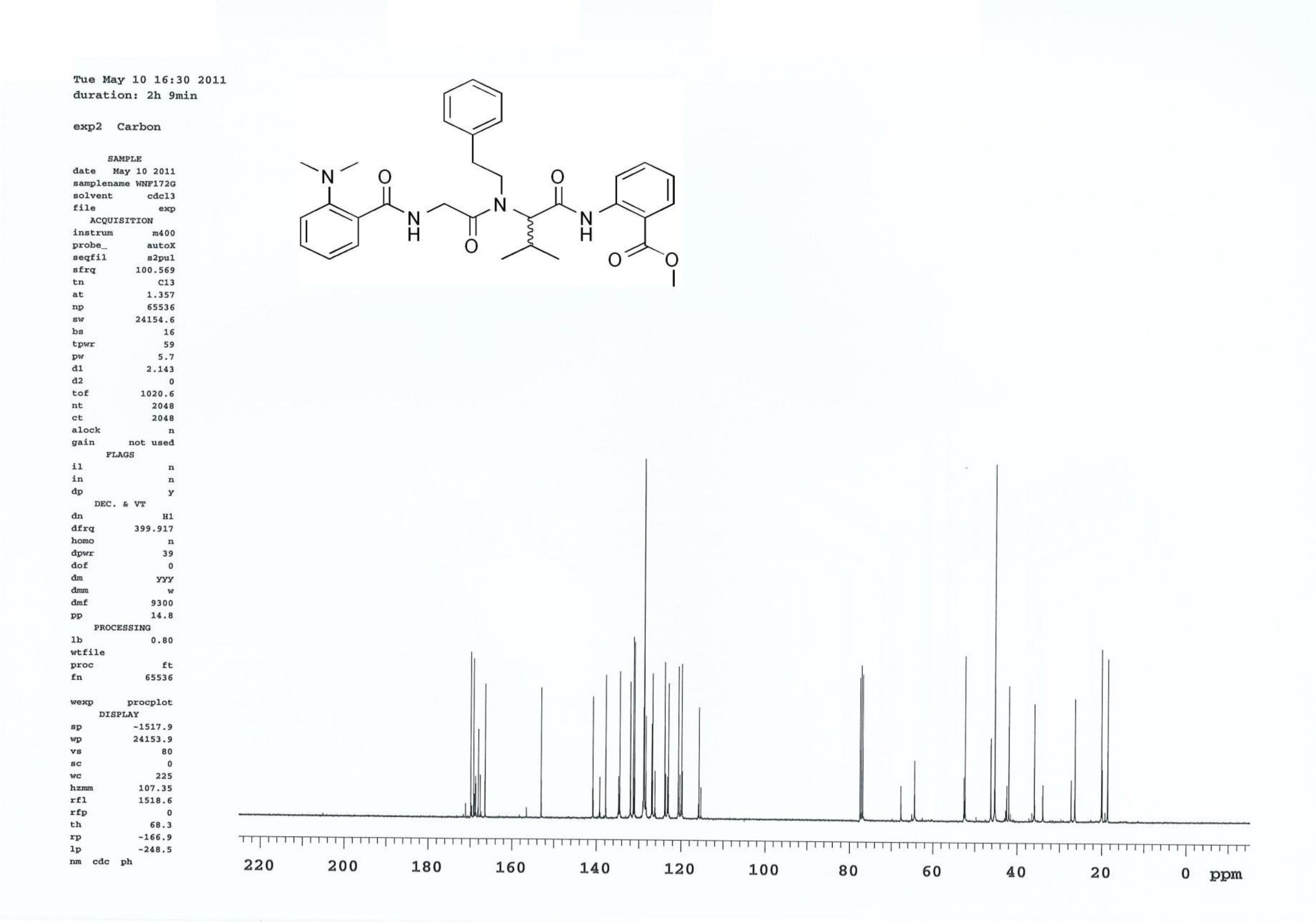

Figure S28: ^{13}C NMR spectrum of compound **13f**.

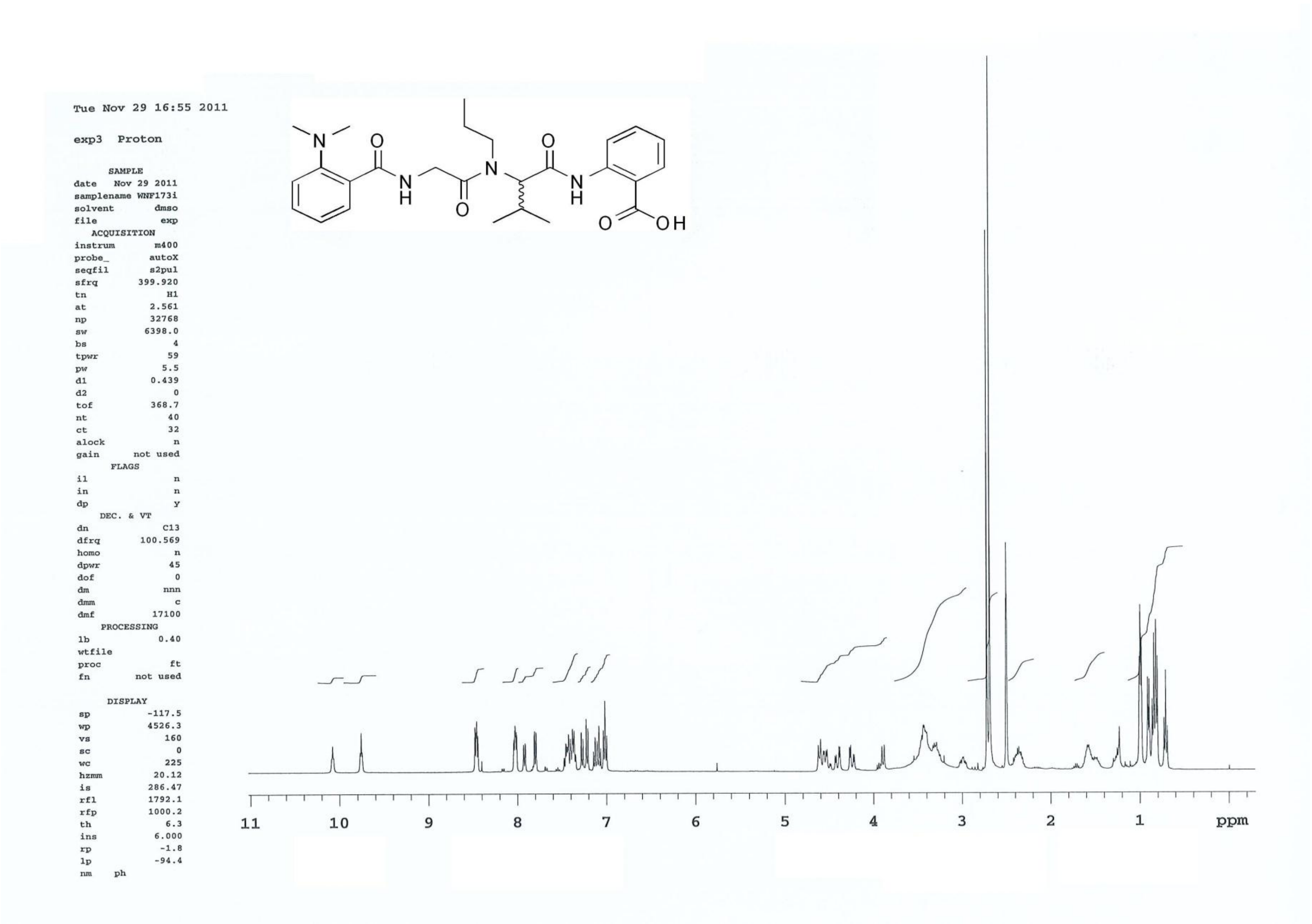

Figure S29: ^{1}H NMR spectrum of compound **14b**.

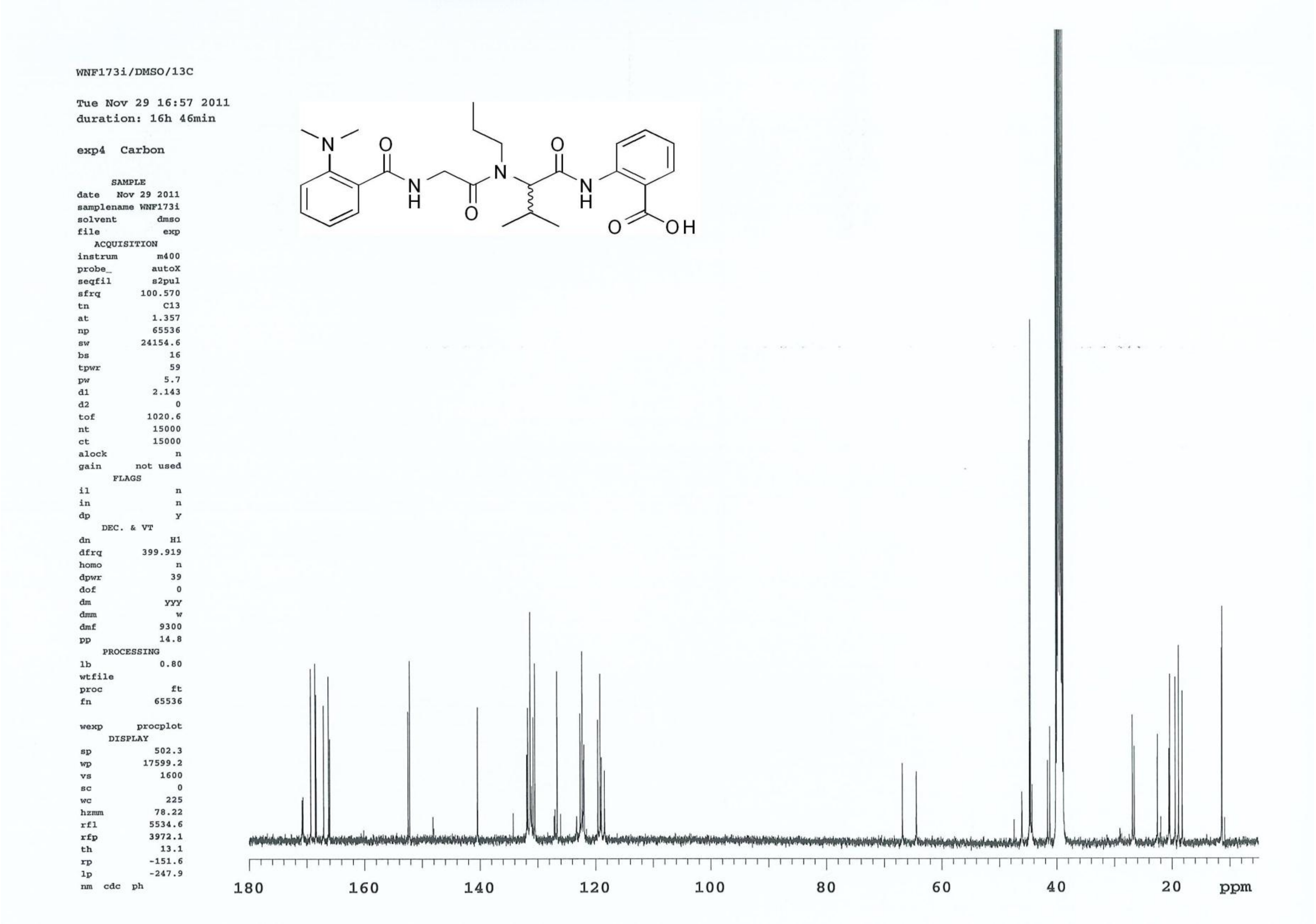

Figure S30: ^{13}C NMR spectrum of compound **14b**.

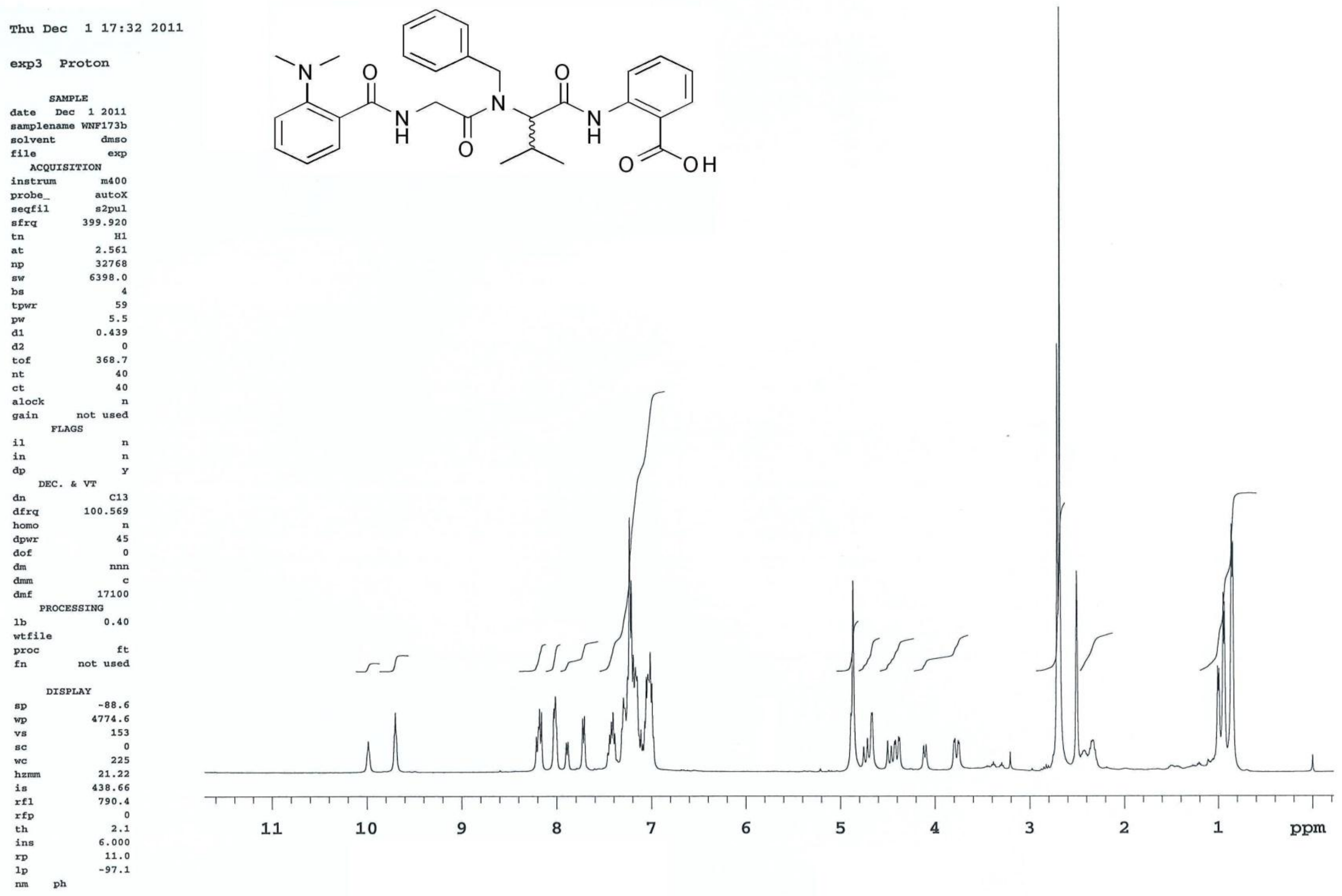

Figure S31: ^{1}H NMR spectrum of compound **14c**.

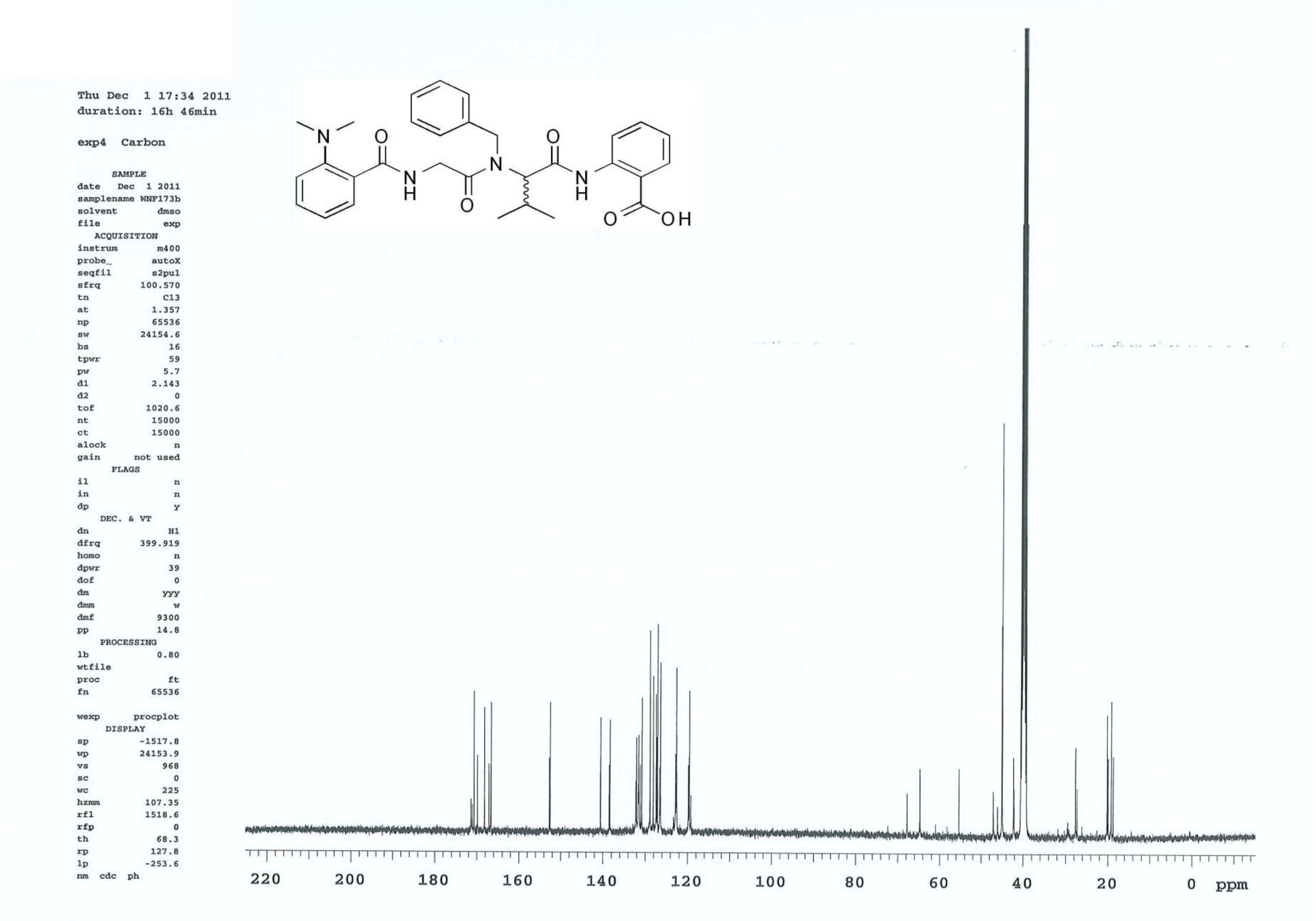

Figure S32: ^{13}C NMR spectrum of compound **14c**.

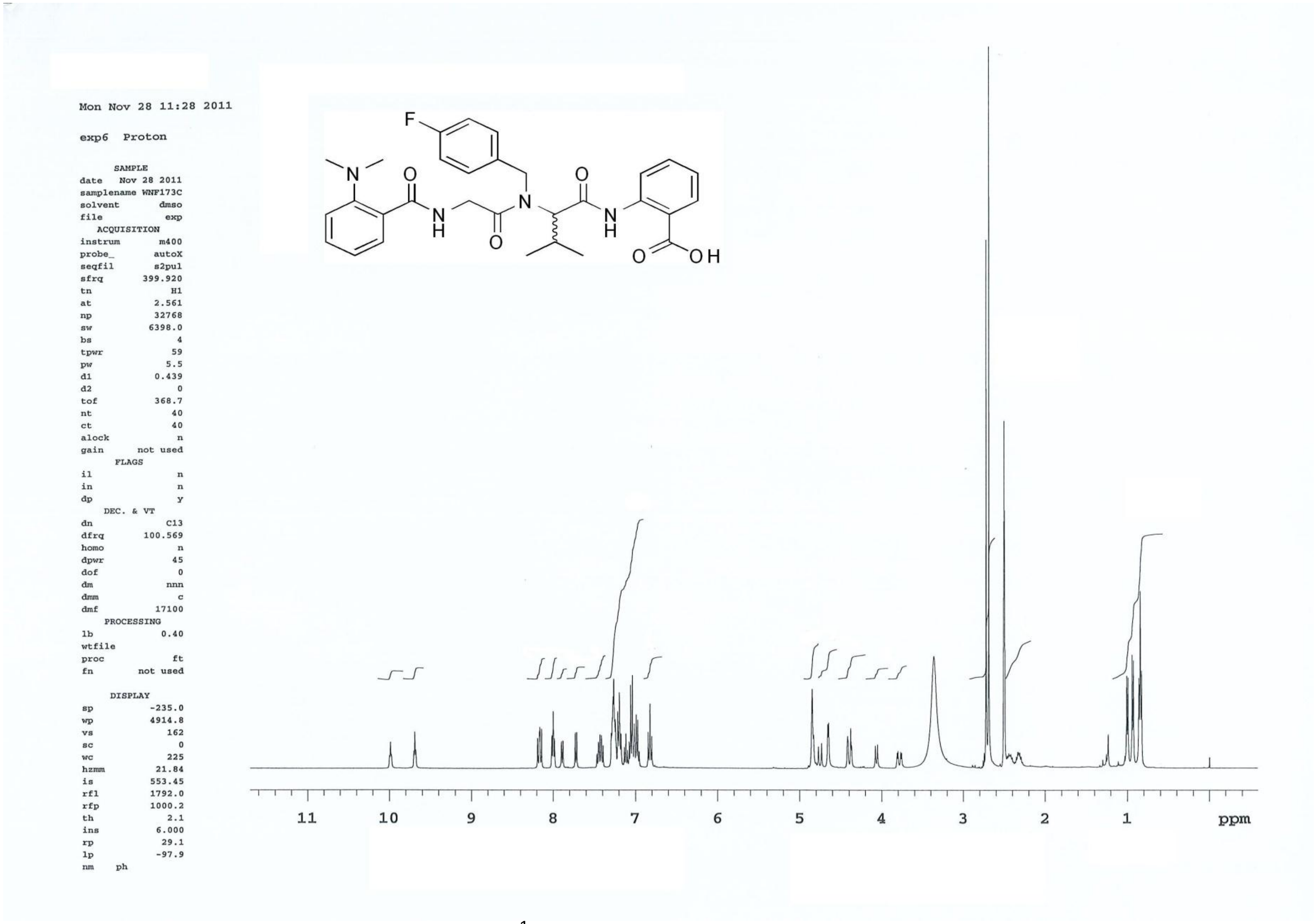

Figure S33: ^{1}H NMR spectrum of compound **14d**.

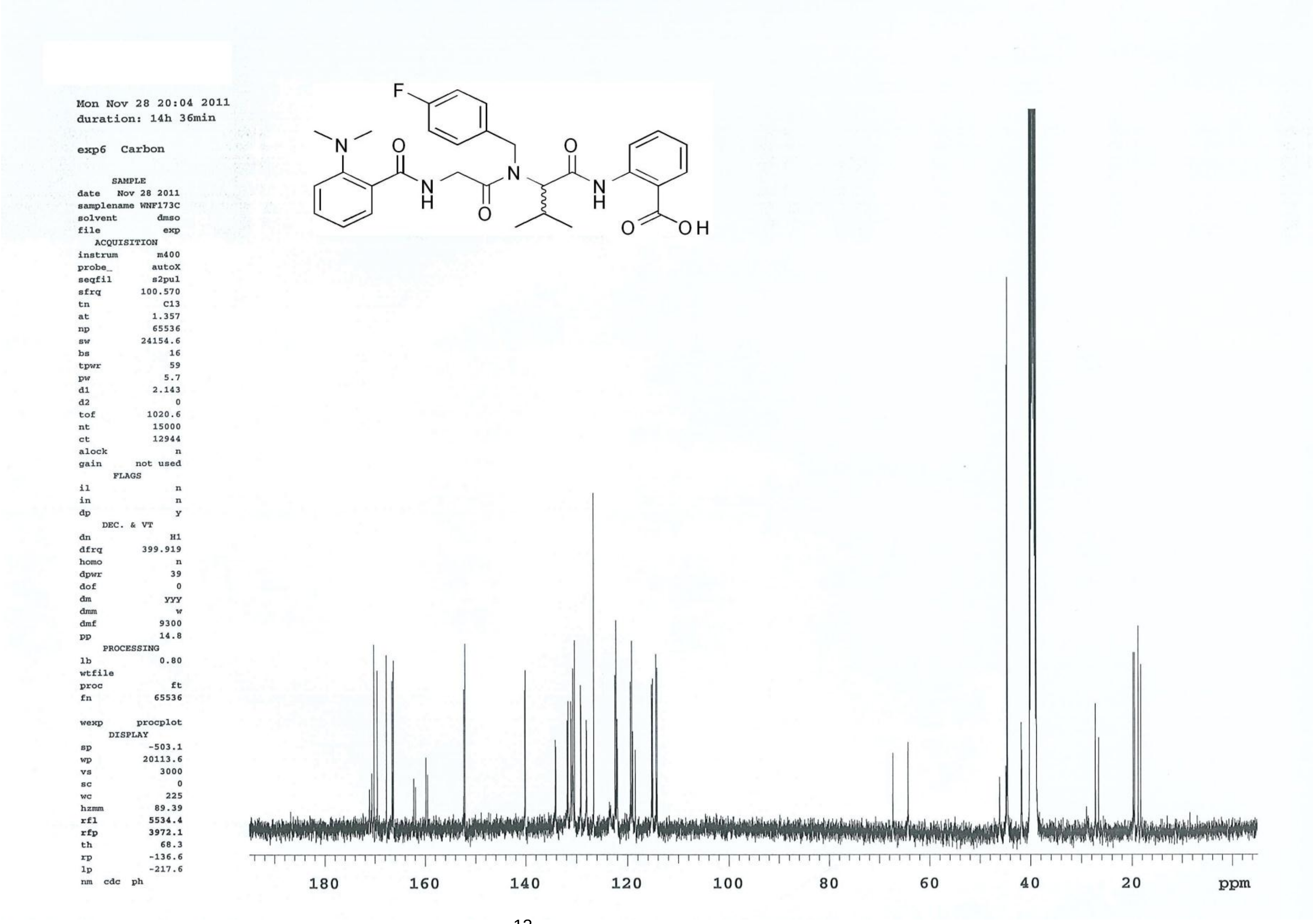

Figure S34: ^{13}C NMR spectrum of compound **14d**.

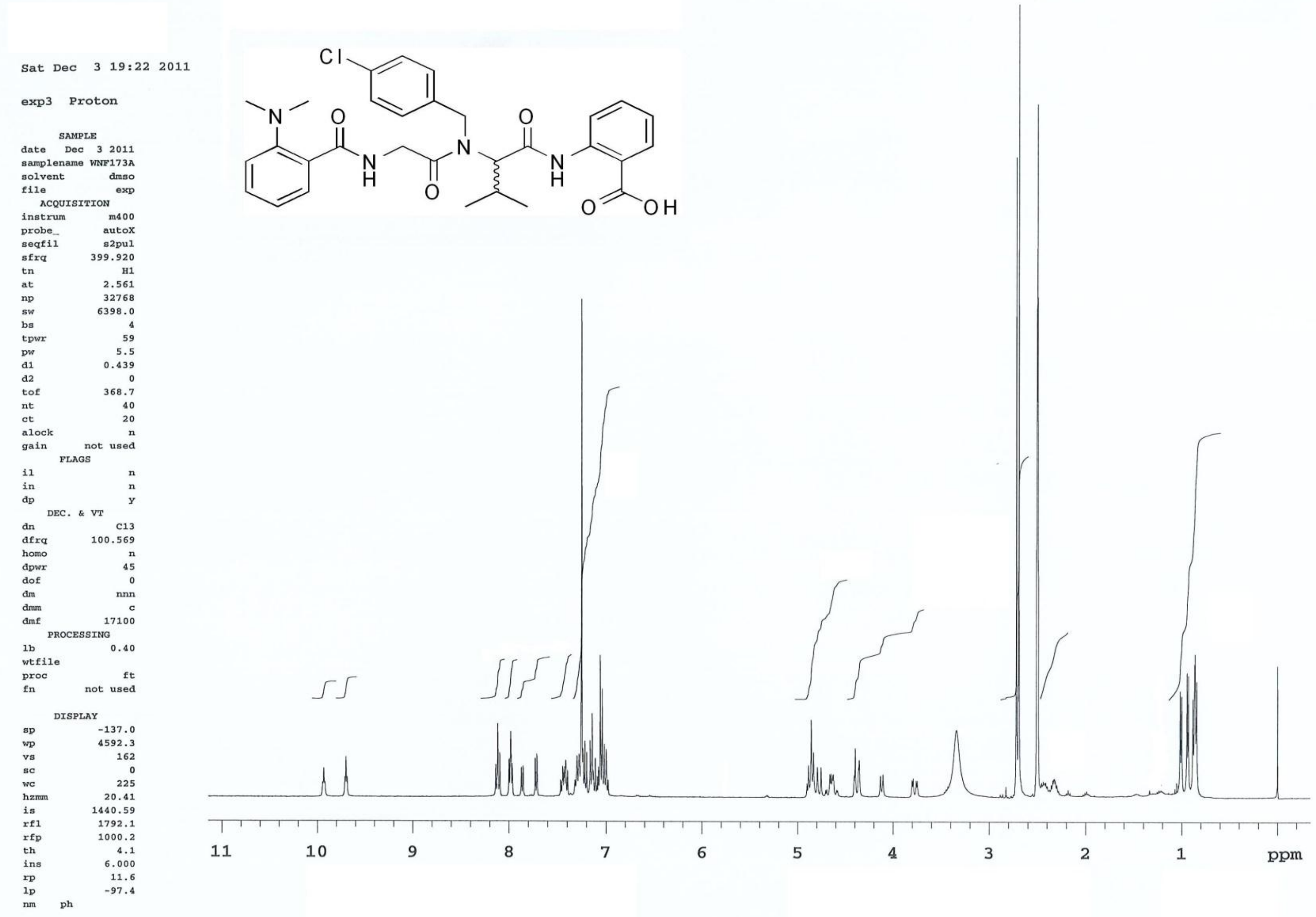

Figure S35: ^{1}H NMR spectrum of compound **14e**.

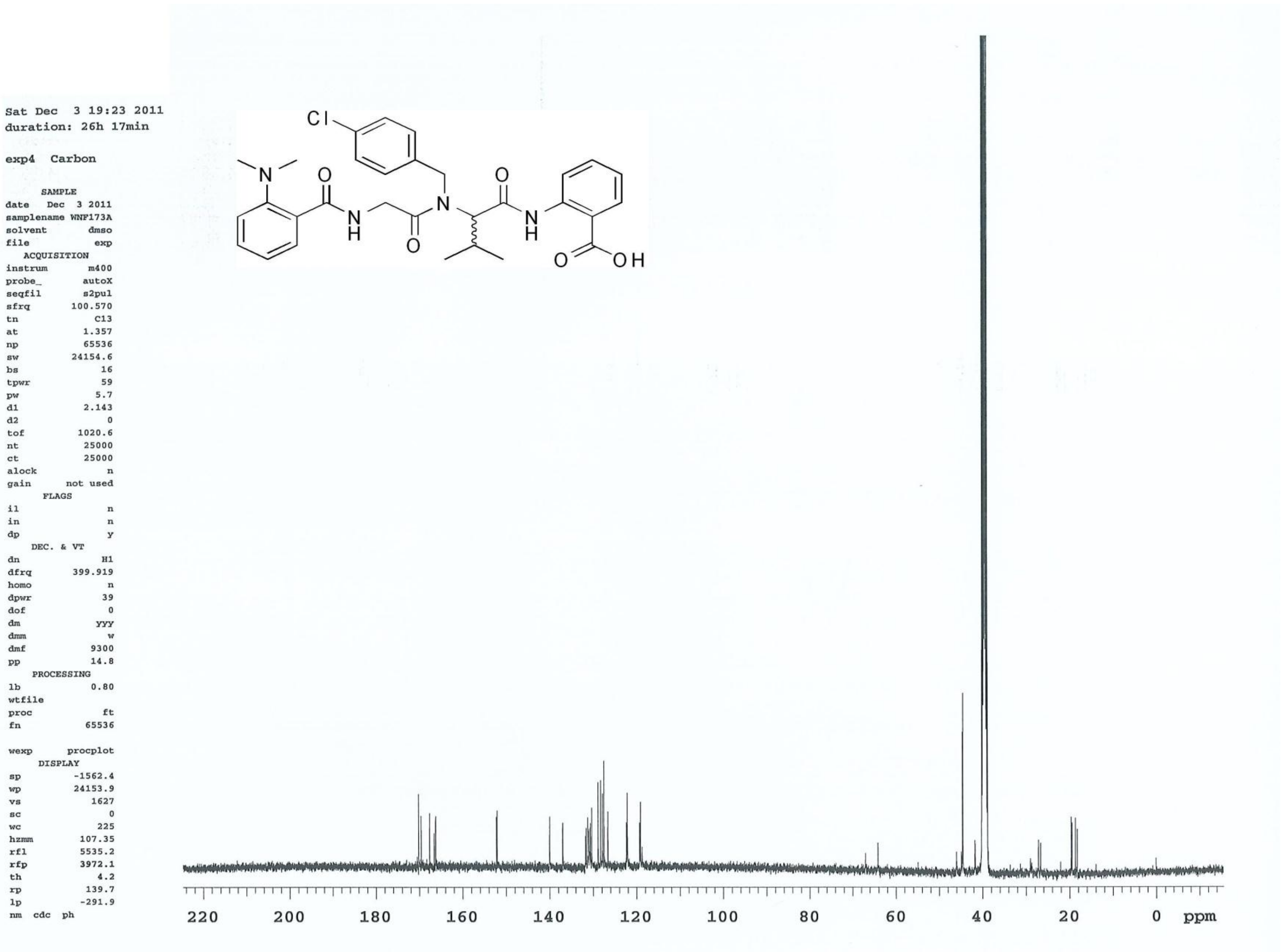

Figure S36: ^{13}C NMR spectrum of compound **14e**.

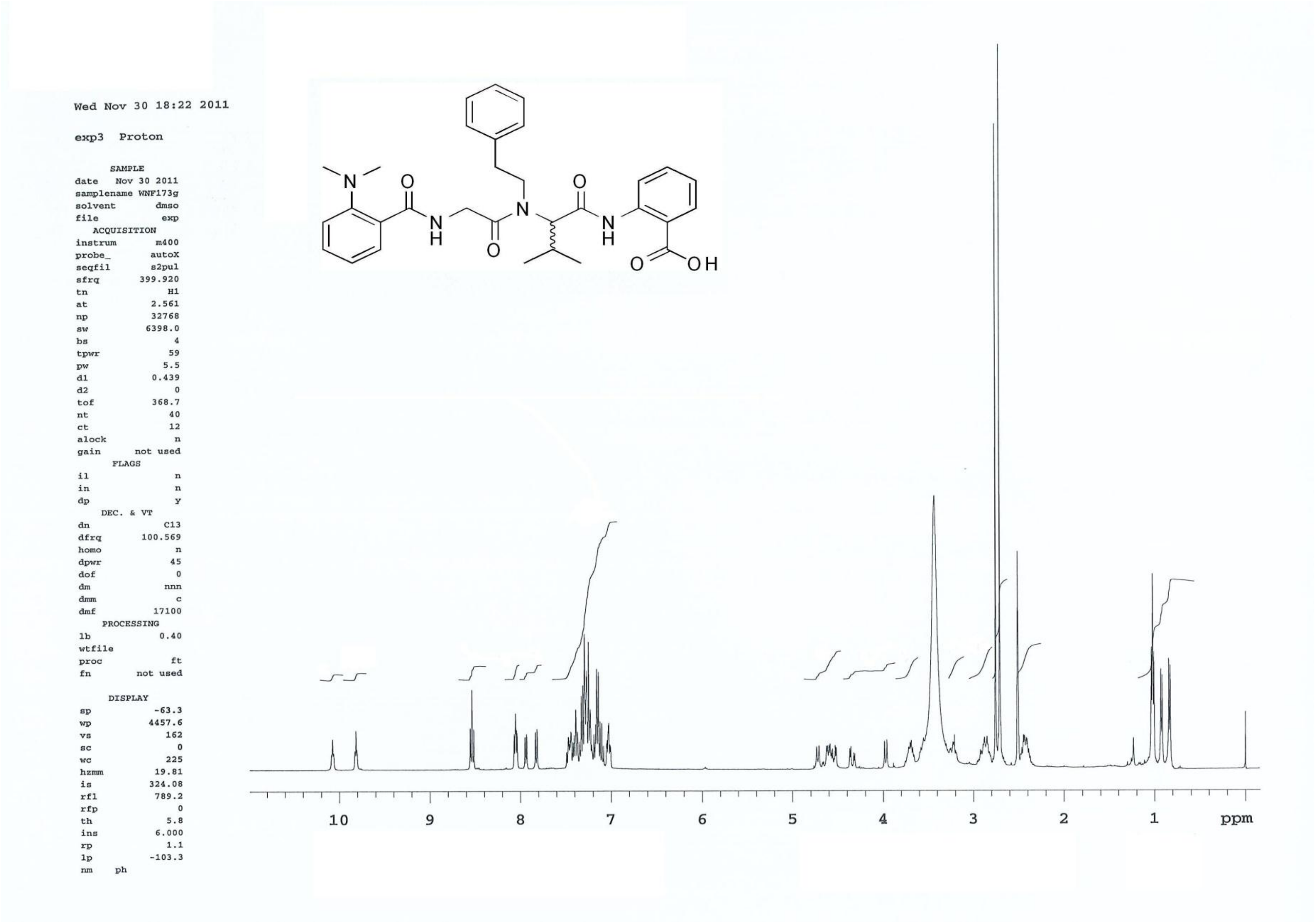

Figure S37: ^{1}H NMR spectrum of compound **14f**.

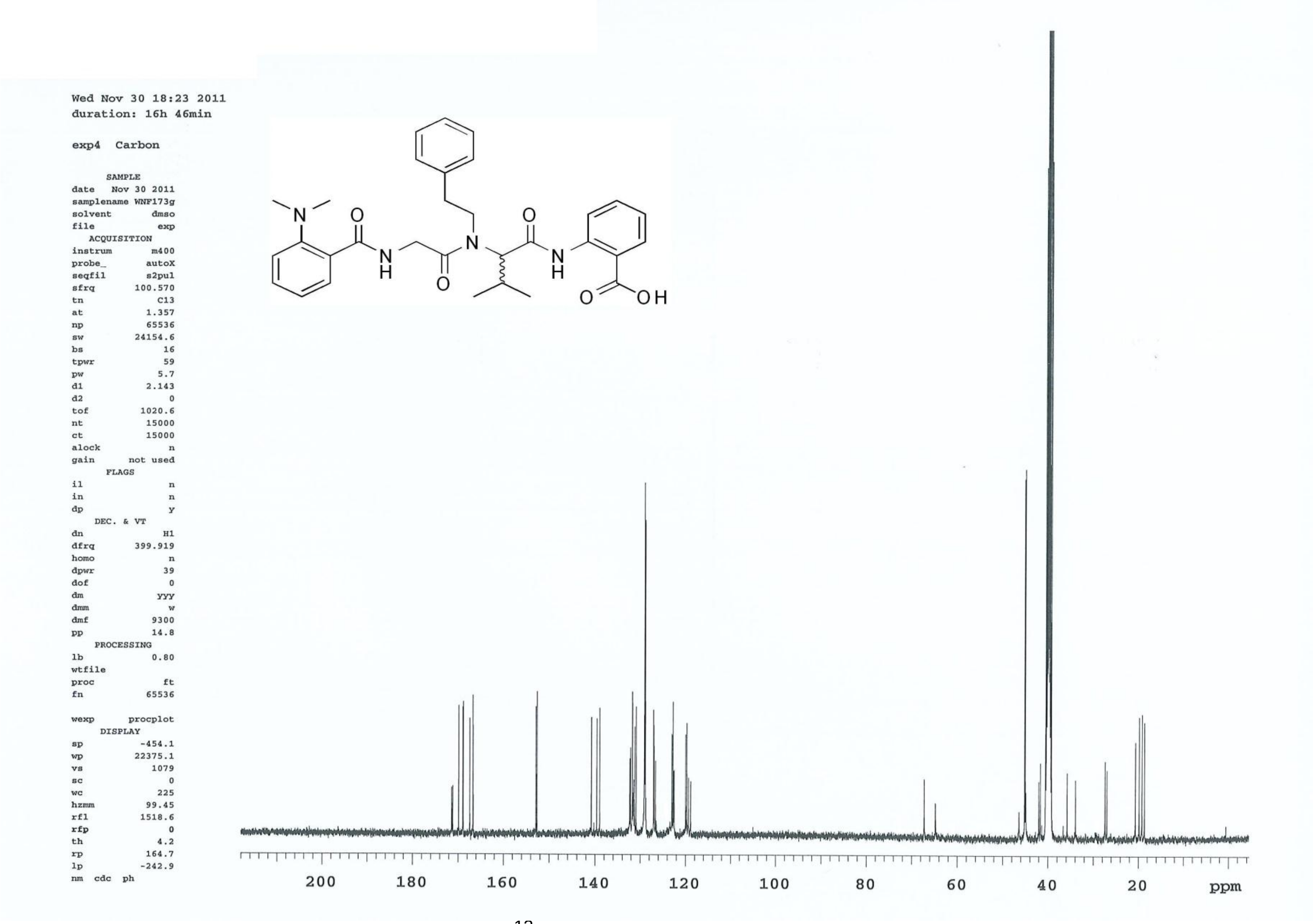

Figure S38: ^{13}C NMR spectrum of compound **14f**.

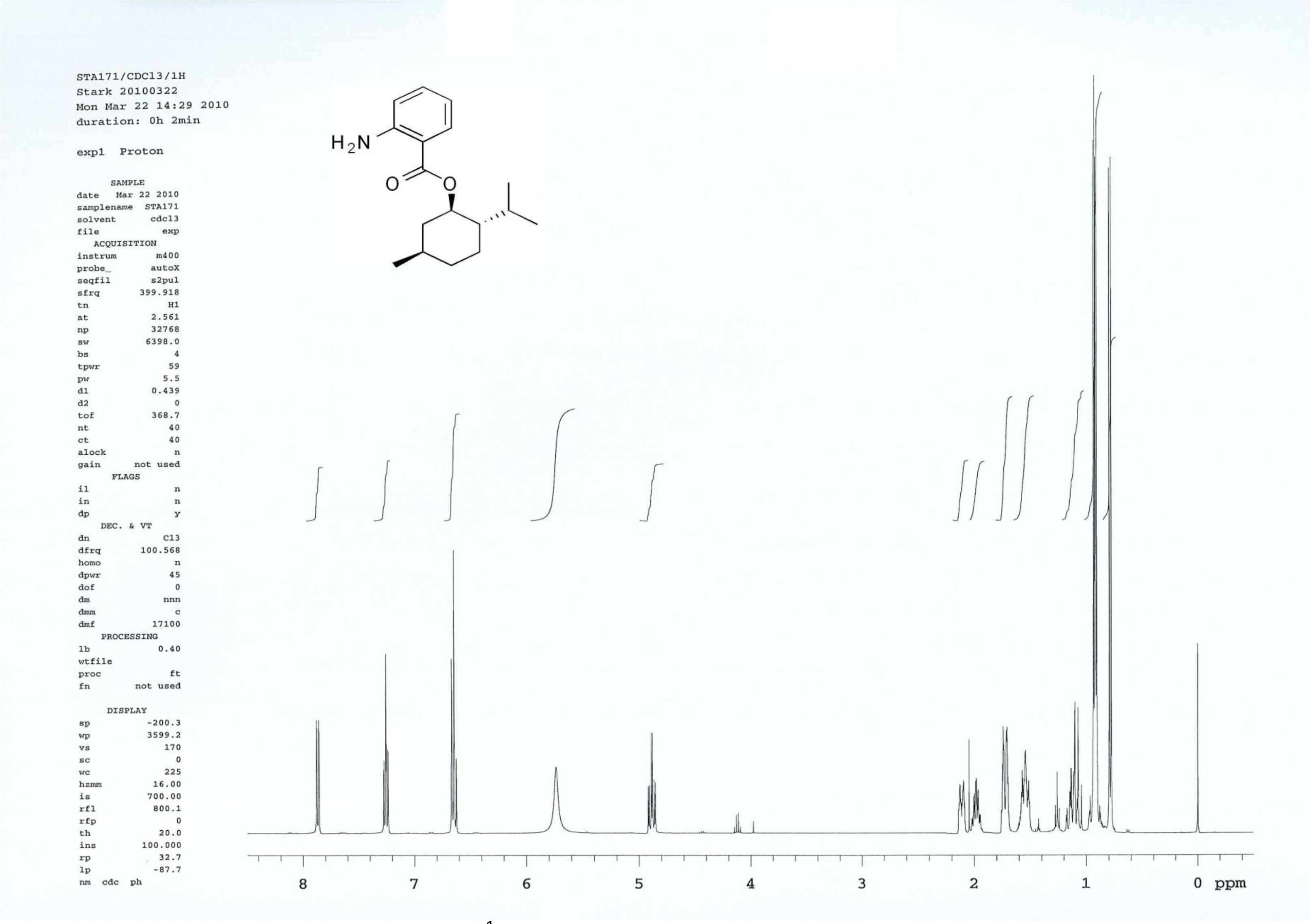

Figure S39: ^{1}H NMR spectrum of menthyl anthranilate.

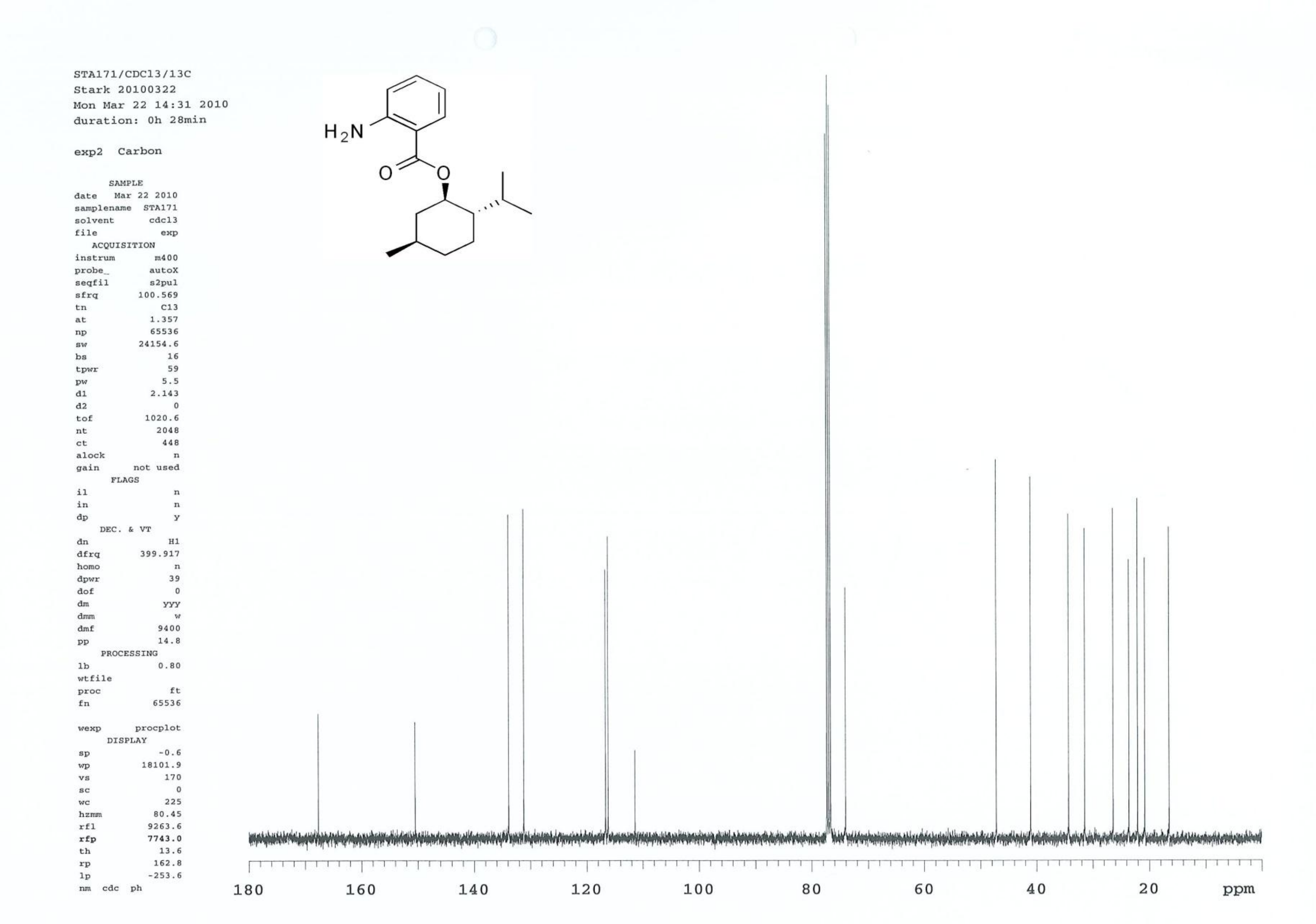

Figure S40: ^{13}C NMR spectrum of menthyl anthranilate.

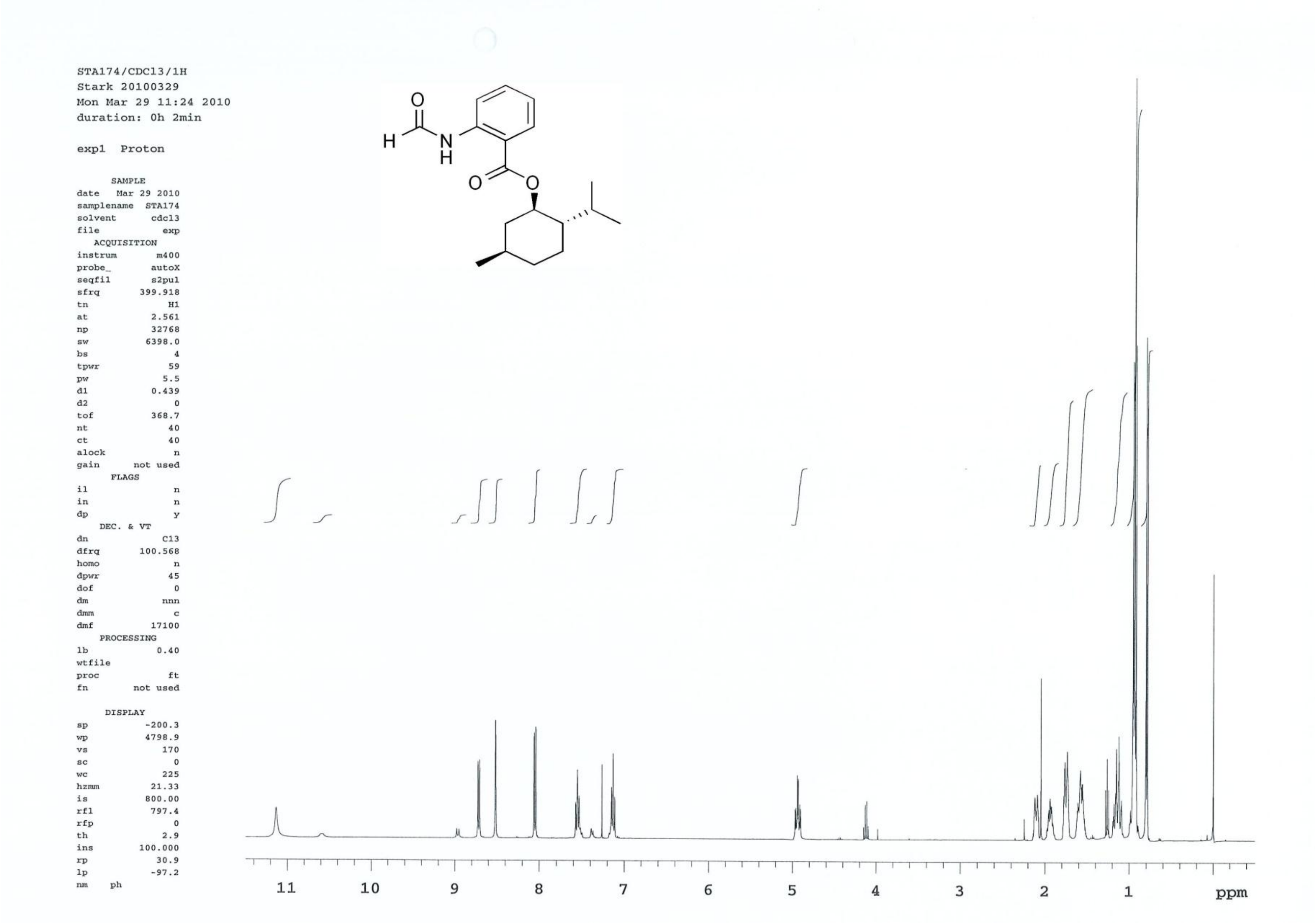

Figure S41: ^{1}H NMR spectrum of (-)-menthyl 2-formamidobenzoate.

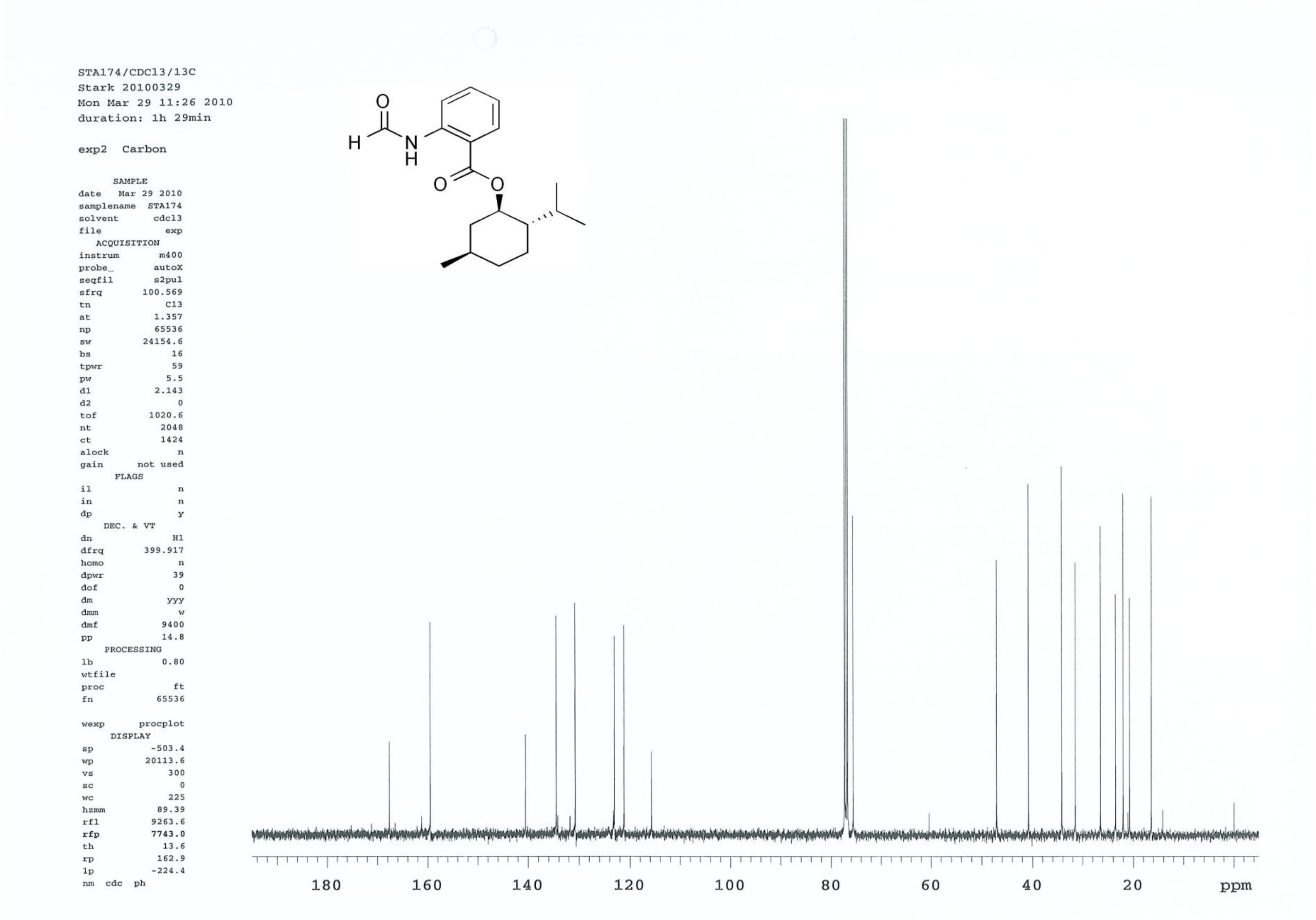

Figure S42: ^{13}C NMR spectrum of (-)-menthyl 2-formamidobenzoate.

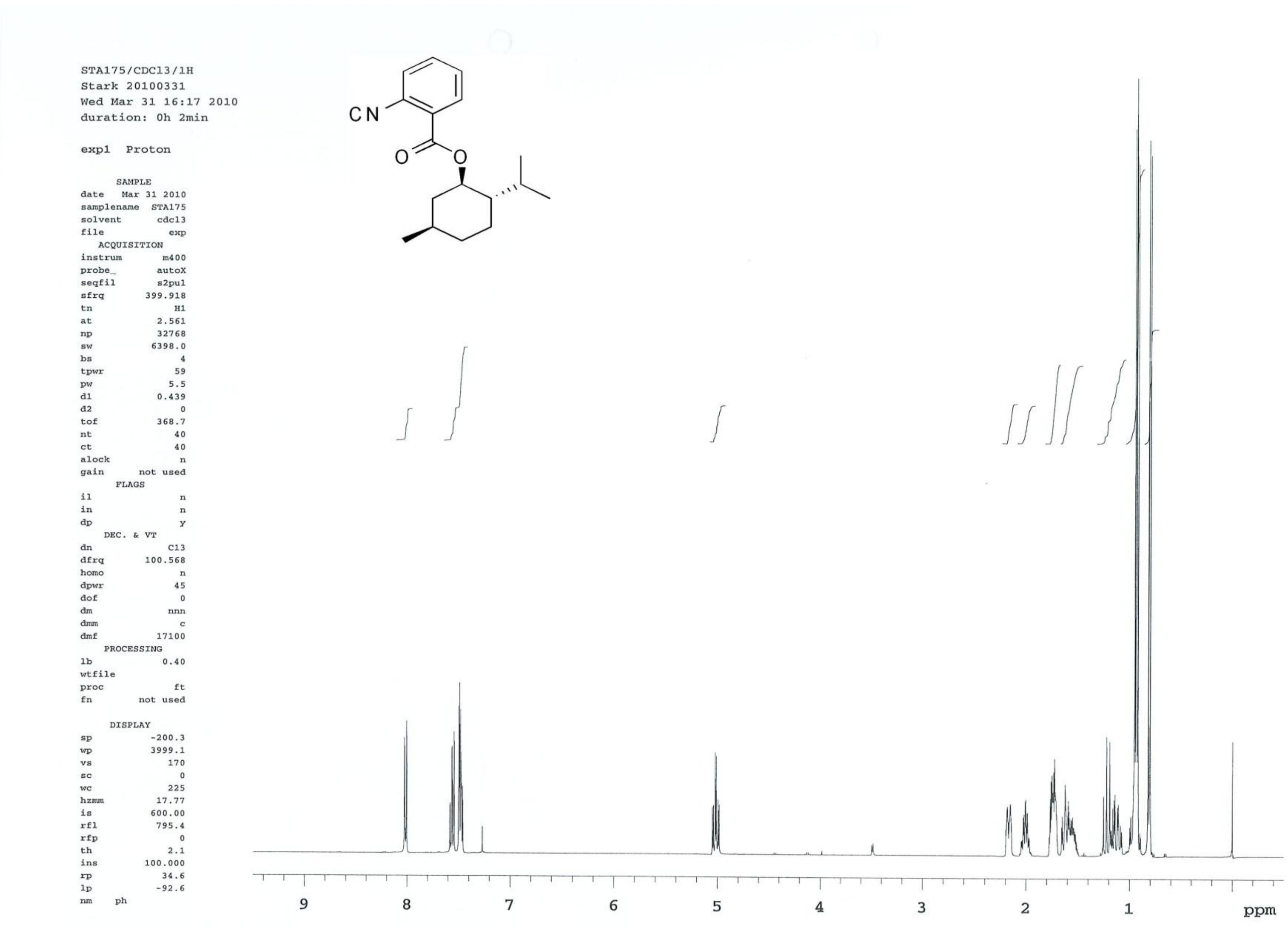

Figure S43: ^{1}H NMR spectrum of compound **4b**.

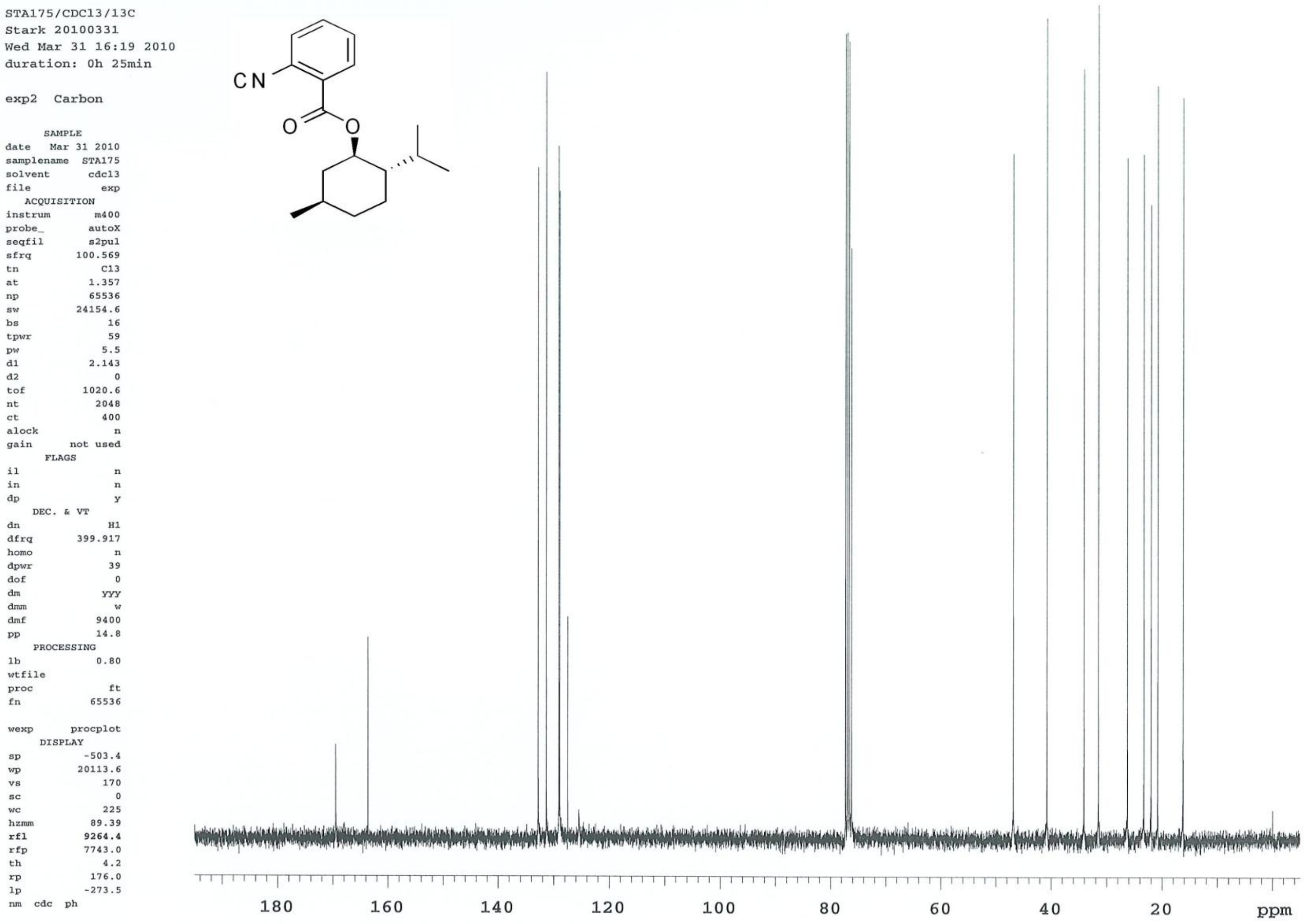

Figure S44: ^{13}C NMR spectrum of compound **4b**.

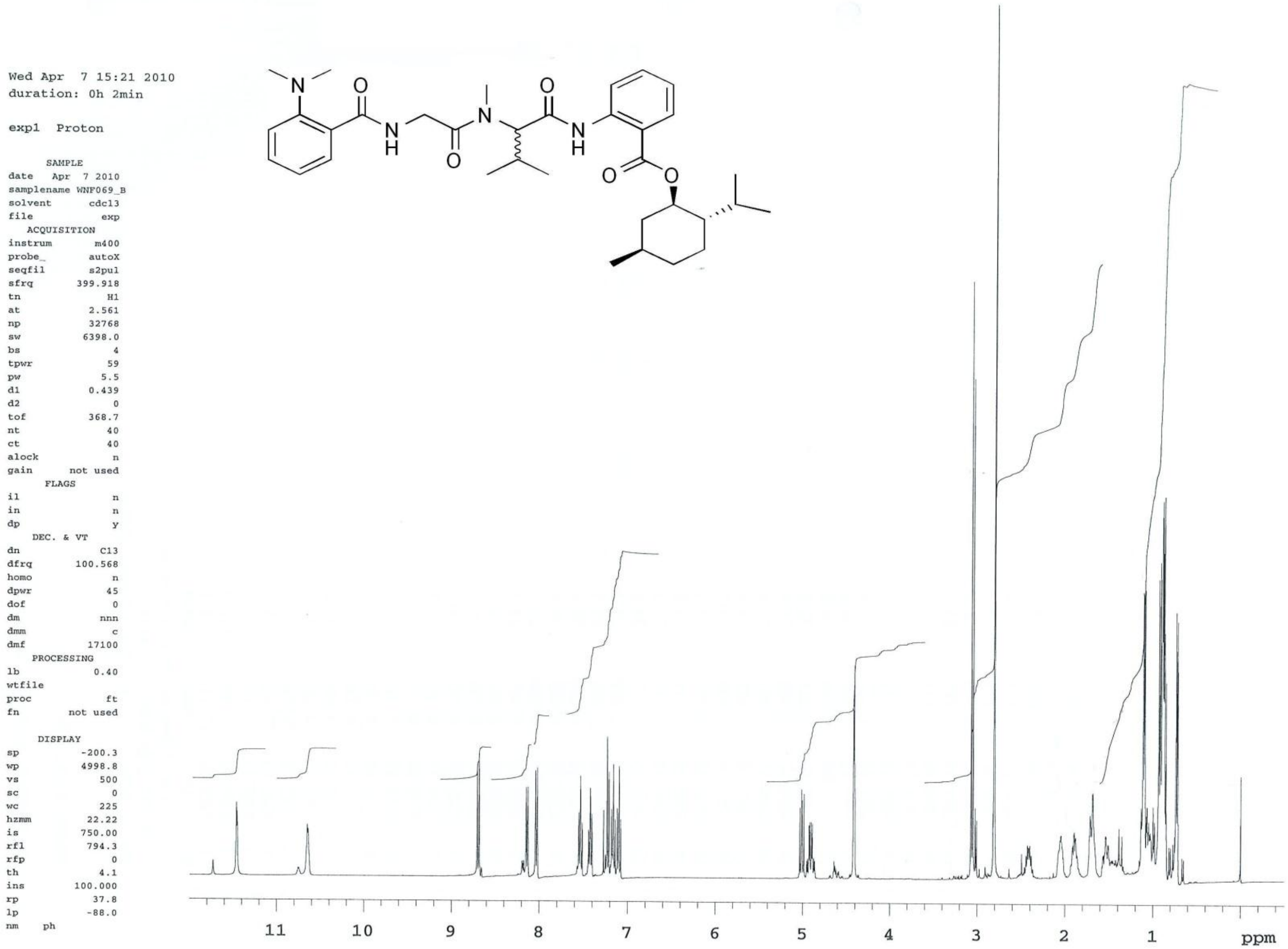

Figure S45: ^{1}H NMR spectrum of compound **13g**.

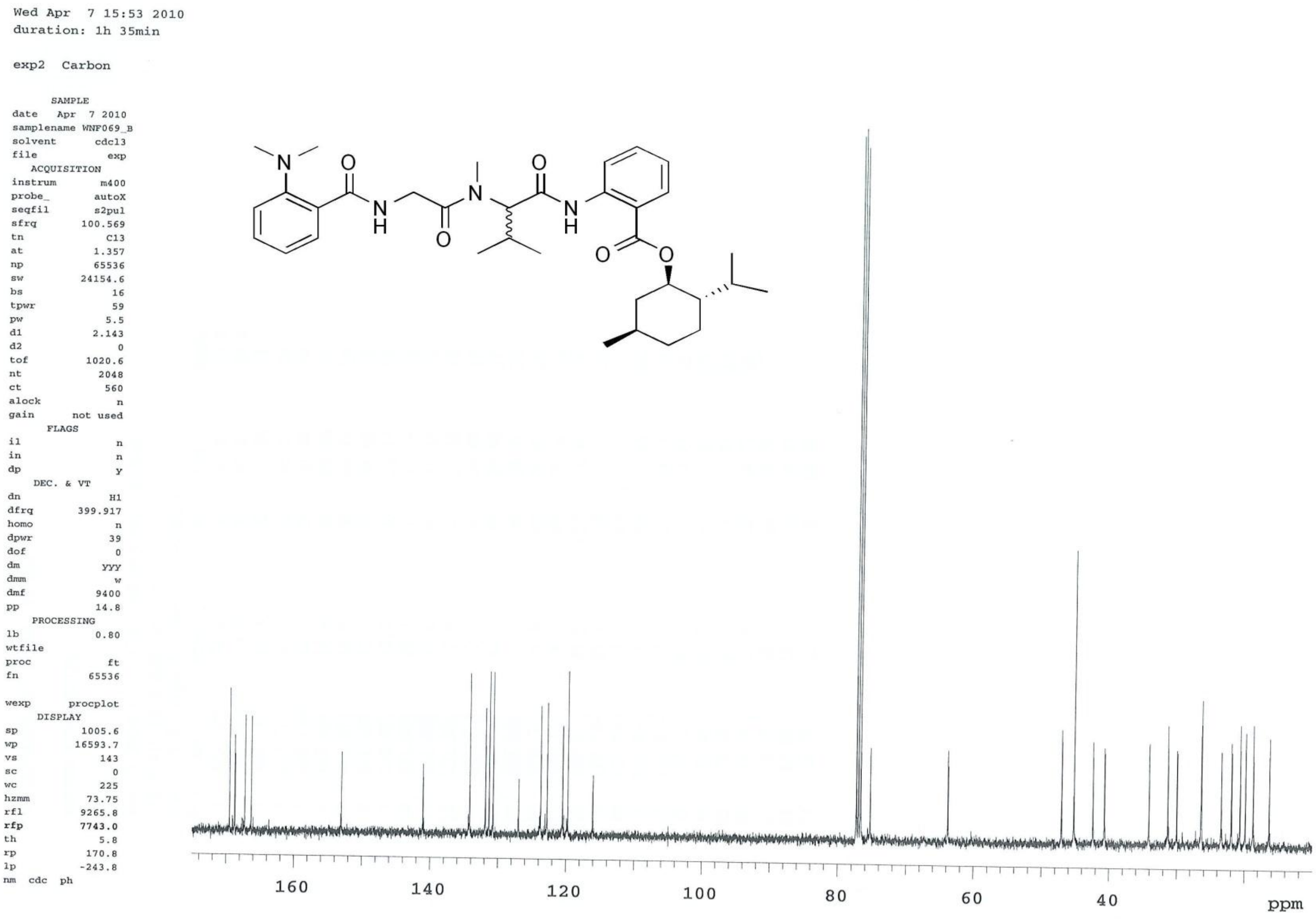

Figure S46: ^{13}C NMR spectrum of compound **13g**.
